# A novel method of literature mining to identify candidate COVID-19 drugs

**DOI:** 10.1093/bioadv/vbab013

**Published:** 2021-07-22

**Authors:** Tomonari Muramatsu, Masaru Tanokura

**Affiliations:** Research Center for Food Safety and Department of Applied Biological Chemistry, Graduate School of Agricultural and Life Sciences, The University of Tokyo, 1-1-1 Yayoi, Bunkyo-ku, Tokyo 113-8657, Japan

## Abstract

**Summary:**

COVID-19 is a serious infectious disease that has recently emerged and continues to spread worldwide. Its spreading rate is too high to expect that new specific drugs will be developed in sufficient time. As an alternative, drugs already developed for other diseases have been tested for use in the treatment of COVID-19 (drug repositioning). However, to select candidate drugs from a large number of compounds, numerous inhibition assays involving viral infection of cultured cells are required. For efficiency, it would be useful to narrow the list of candidates down using logical considerations prior to performing these assays. We have developed a powerful tool to predict candidate drugs for the treatment of COVID-19 and other diseases. This tool is based on the concatenation of events/substances, each of which is linked to a KEGG (Kyoto Encyclopedia of Genes and Genomes) code based on a relationship obtained from text mining of the vast literature in the PubMed database. By analyzing 21 589 326 records with abstracts from PubMed, 98 556 KEGG codes with NAME/DEFINITION fields were connected. Among them, 9799 KEGG drug codes were connected to COVID-19, of which 7492 codes had no direct connection to COVID-19. Although this report focuses on COVID-19, the program developed here can be applied to other infectious diseases and used to quickly identify drug candidates when new infectious diseases appear in the future.

**Availability and implementation:**

The programs and data underlying this article will be shared on reasonable request to the corresponding authors.

**Contact:**

atmuramatsu@g.ecc.u-tokyo.ac.jp, amtanok@mail.ecc.u-tokyo.ac.jp

**Supplementary information:**

Supplementary data are available at *Bioinformatics Advances* online.

## 1 Introduction

COVID-19 is an infectious disease that emerged in December 2019. As of May 2021, 159 million cases and 3.3 million deaths were reported, and COVID-19 continues to spread worldwide (WHO, 2021). For the effective and rapid selection of candidate drugs, preliminary screening may be required prior to cell culture and/or *in silico* screening because the number of drugs that can be tested using cultured cells or viral enzyme docking simulations is limited. Thus, we developed a novel and powerful system of text mining using PubMed (National Center of Biotechnology Information, 2021) to search for drug candidates. PubMed is a database with an extensive volume of literature in biomedical scientific fields, containing approximately 30 million papers and 20 million abstracts to date. To derive a relationship between two biomedical terms, we counted the number of papers in which these terms coexisted. Each of the search terms used was obtained from the KEGG (Kyoto Encyclopedia of Genes and Genomes; Kanehisa *et al.*, 2017) NAME and DEFINITION fields so that they could be linked to KEGG codes. The KEGG (Kanehisa *et al.*, 2017) is an excellent database of biomolecular materials, reactions, genes and organisms. Thus, our system aimed to deduce a relationship between KEGG codes using PubMed abstracts. A more important purpose of this system is to predict existing drugs that would be useful for underinvestigated diseases. Thus, the KEGG code–KEGG code relationships identified were not restricted to those between the disease code (H code) and drug code (D code) but included all usable codes, such as proteins (K code), metabolites (C code), enzymes (EC code), glycans (G code), reactions (R code) and drug groups (DG code). Then, we aimed to derive the relationship between disease and drug through the path created by these KEGG code–KEGG code relationships.

PubMed (National Center of Biotechnology Information, 2021) is the main source of medical biology literature information available to the scientific community. Our method derives relationships between pairs of terms in PubMed abstracts. In general, text mining (Xue *et al.*, 2018) uses all PubMed data as one corpus and does not distinguish between individual records (PubMed IDs). In contrast, our method links each relationship to the relevant PubMed IDs. We used the co-occurrences in the abstracts, not the relationships in the sentences or the narrower regions that are commonly used in text-mining research, to obtain sufficient information (Kwon *et al.*, 2017).

## 2 Methods

### 2.1 Databases

PubMed xml files were downloaded from the PubMed ftp site (https://www.nlm.nih.gov/databases/download/pubmed_medline.html).

As of April 25, 2021, PubMed contained 32 422 790 IDs, of which 21 589 326 had abstracts. To use the KEGG database, an FTP download license was purchased from Pathway Solutions, Inc. (Tokyo, Japan), and data (FTP release April 26, 2021) were downloaded from the site (ftp://ftp.kegg.net/kegg). A stemmer program was used to unify the expression of each word/phrase to treat multiple expressions of a word/phrase as the same unit. For this purpose, the SnowBall stemmer (Porter, 1980) was downloaded from https://snowballstem.org/, and we added some properties to create a new program through inheritance.

### 2.2 Selection of search terms

In the KEGG database, the NAME field and DEFINITION field were selected to produce search terms/phrases. The included search terms and phrases were formed by dividing phrases by punctuations and/or parentheses, removing very frequently emerging words in the KEGG NAME/DEFINITION fields, such as hydrochloride, hydrate and sodium, removing descriptions about configuration, and other transformations. This approach created search terms/phrases that were added to the terms/phrase list for each KEGG code instead of being replaced to ensure that the PubMed abstract search did not miss any relevant records.

### 2.3 Computer system

A DELL EMC PowerEdge R740 system with two Intel Xeon Silver 4114 processors with 384 GB RDIMM was used. The operating system and programming language were Ubuntu 18.04 LTS and Python 3.69, respectively.

### 2.4 Updating

Our database system is updated daily regarding PubMed data and every 2 weeks regarding KEGG data.

## 3 Results and discussion

### 3.1 Search terms and usage of the stemmer program

Depending on the context, English words have many forms of variation. Therefore, in text-mining procedures, it is common to perform word stemming by stemmer programs such as SnowBall Stemmer (Porter, 1980). This type of procedure converts all letters in a word to lower case and removes some letters that change from time to time at the end of the word. These steps treat multiple expressions of a word/phrase as the same unit. For example, the difference between these expressions is the singular and plural forms of the noun, the conjugation of the verb and the case of the first letter of the word depending on the location (first or center) of the sentence. Stemmers are used to unify the expression. Both the search term/phrase and the abstract were stemmed to determine the presence of the search term/phrase in the PubMed abstracts. However, in the biomedical field, many upper case abbreviations, such as ‘SARS’, are continuously introduced. We think conversion to lower case and stemming of these words would reduce their specificities. Moreover, the word endings of many technical terms, such as -ase, -ose, -ine and -sis, must not be deleted. Therefore, we created a new stemmer program through inheritance by adapting the SnowBall English Stemmer. Our adapted program converts the first character of a word to upper case, but neither upper case to lower case nor lower case to upper case conversions occur in other positions to ensure that upper case abbreviations are conserved. Excisions from the word ending are the same as Snowball English Stemmer with exceptions of uppercase abbreviations, ‘-ase’, ‘-ose’, ‘-ine’ and ‘-sis’, where excision occurs up to the end of these regions.

### 3.2 Distance between two KEGG codes

**Fig. 1.**
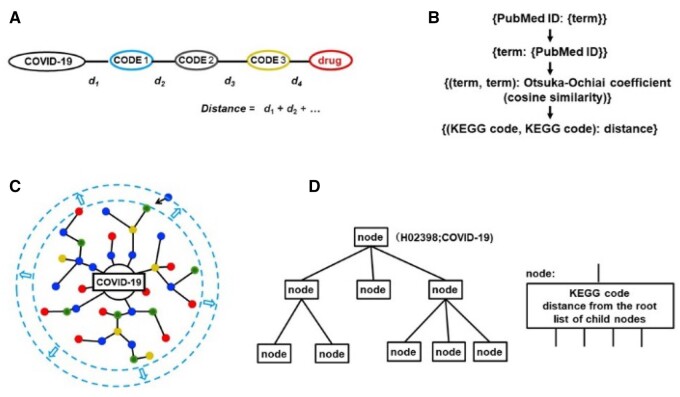
Program design. (**A**) The distance between concatenated codes was calculated by adding componential distances, each of which was defined as the distance between two KEGG codes described in (**B**). (B) The distance between two KEGG codes was defined by the number of papers (PubMed IDs) in which terms from the KEGG pair coexisted. (**C**) A new node (KEGG code) is added to a path from COVID-19 to create a tree structure (**D**)

We aimed to predict the relationship between unreported diseases and drugs by concatenating the relationships between KEGG codes (Fig. 1A). To achieve this, we needed a way to connect relationships. We have developed the following concepts. The probability of the existence of term B in an abstract under the condition that term A exists in the same abstract is defined as follows:

$$P_{A}\left(B\right)=\frac{P\left(A\cap B\right)}{P\left(A\right)}=\frac{f\left(A\cap B\right)}{f\left(A\right)},$$

where $P\left(A\right)$ and $P\left(A\cap B\right)$ are probabilities of the existence of A and co-occurrence of A and B, respectively, and $f\left(A\right)$ and $f\left(A\cap B\right)$ are the numbers of PubMed abstracts that contain term A and terms A and B (frequencies). Thus, the probability of A and B coexisting is expressed as follows:

$$P\left(A\cap B\right)=P\left(A\right)\times P_{A}\left(B\right).$$

Similarly, for the A-B-C connection, the following expression is used:

$$P\left(A\cap B\cap C\right)=P\left(A\cap B\right)\times P_{A\cap B}\left(C\right)=P\left(A\right)\times P_{A}\left(B\right)\times P_{A\cap B}\left(C\right).$$

Assuming that the relationship between B and C is independent of A, i.e. $P_{A\cap B}\left(C\right)=P_{B}\left(C\right)$, then,

$$P\left(A\cap B\cap C\right)=P\left(A\right)\times P_{A}\left(B\right)\times P_{B}\left(C\right).$$

Although this assumption is not strictly correct, most experimental biological studies dealing with multistep reactions use this assumption as the first approximation. With this assumption, the probability of this pathway can be estimated by successively multiplying each of the conditional probabilities. Instead of multiplying the probabilities, the distance between two terms, which is defined as $\mathrm{distance}\left(A,B\right)=-\ln\left(P_{A}\left(B\right)\right)$, can be added successively.

Then, terms were connected one by one as nodes to create a tree structure using COVID-19 as the root according to the calculated distances. However, each term had a varying specificity across the range to phenomena, and less specific terms tended to be selected as the term B of $P_{A}\left(B\right)$ and listed as candidate drugs. A less ideal tree model was obtained (data not shown) because the dominator in $P_{A}\left(B\right)$ was only related to term A. To increase specificity, we proposed that feedback from term B is required and tried two functions instead of conditional probability: Dice index (Dice, 1945; Sørensen, 1948), $\mathrm{Dice}\left(A,B\right)=\frac{2\times f\left(A\cap B\right)}{f\left(A\right)+f\left(B\right)}$, and Otsuka-Ochiai coefficient (Ochiai, 1957), $K\left(A,B\right)=\frac{f\left(A\cap B\right)}{\sqrt{f\left(A\right)\times f\left(B\right)}}$, where $f\left(A\right)$ and $f\left(B\right)$ indicate the frequency of existence of term A and term B in a PubMed abstract, respectively, and $f\left(A\cap B\right)$ represents the frequency of co-occurrence of term A and term B in the same abstract. From another point of view, the Otsuka-Ochiai coefficient is the same as the cosine similarity of each combination of words represented as a vector in the dimensional space of the number of PubMed IDs, where each value of the vector element in that space is 1 or 0 depending on its existence/nonexistence. Moreover, to enhance specificity and to prevent missing the connectivity of the phenomena, our system used the relationship of the KEGG code base (KEGG code–KEGG code relationship) instead of the term-based relationship. For KEGG codes X and Y, all dice index values/Otsuka-Ochiai coefficients between any combination of terms x and y belonging to codes X and Y, respectively, were calculated. Their maximum value was then used to indicate the degree of relationship between the two KEGG codes (X and Y), and then the distance between two KEGG codes was defined as follows with the assumption that distances are additive (Fig. 1B):

$$\mathrm{distance}\left(X,Y\right)=-\ln\left(\max\left(\mathrm{coeff}\left(x,y\right)\right)\right),$$

where *coeff*(x, y) is the Dice or Otsuka-Ochiai coefficient between terms x and y, which are related to KEGG codes X and Y, respectively.

Next, we developed a method to connect KEGG–KEGG interactions (Fig. 1C). These KEGG codes were deployed as nodes in a tree structure (Fig. 1D) according to the calculated distance from the COVID-19 code (H02398) at the root; the codes were connected one by one to an appropriate node that was already connected. Then, candidate drugs were listed according to decreasing distance from the disease. To assess the additivity of the distances and validity of the tree models constructed using the Dice index and Otsuka-Ochiai coefficient (Ochiai, 1957), the list of the direct distances from the direct relationships and the indirect distances calculated by adding the distances through the path in the tree obtained by omitting the direct connection was merged in Table 1 (first 50 items) and Supplementary Table S1 (first 1000 items) for comparison. Distances using the Otsuka-Ochiai coefficient demonstrated good agreement between direct and indirect connections. However, agreement using the Dice index method and that using the conditional probability method was not as good (the Otsuka-Ochiai coefficient method > the Dice index method ≫ the conditional probability method) (Supplementary Figs S1 and S2 and Tables S2 and S3). To compare the Otsuka-Ochiai coefficient method and the Dice method, we calculated the statistics of the difference between the direct connection and the indirect connection ((indirect connection distance-direct connection distance)/standard deviation of the direct connection distance) for the first 100 items, which should be the least inaccurate data presented in the table. Both the mean value (Otsuka-Ochiai, 0.237; Dice, 0.446) and the standard deviation (Otsuka-Ochiai, 1.031: Dice 1.167) indicated the superiority of the Otsuka-Ochiai method.

**Table 1.** Distances between COVID-19 and each drug calculated by the Otsuka-Ochiai coefficient (cosine similarity) method (first 50 items)

Code	D_id(depth)	D_d	Name
D11472	2.939(2)	2.318	Veklury (TN), Remdesivir (JAN/USAN)
D08050	3.618(2)	2.607	Hydroxychloroquine (INN), Polirreumin (TN)
D09537	3.802(2)	3.137	Favipiravir (JAN/USAN/INN), Avigan (TN)
D01425	4.060(2)	3.191	Lopinavir (JAN/USP/INN)
D02498	4.060(2)	3.191	Lopinavir and ritonavir, Kaletra (TN)
D02596	4.451(2)	3.242	Tocilizumab (genetical recombination) (JAN), Tocilizumab (USAN/INN),
D03043	4.766(2)	3.307	Air (TN), Air, medical (USP)
D05864	4.820(2)	3.339	Sodium monofluorophosphate (USP), Aim (TN)
D00427	4.492(2)	3.595	Ritonavir (JAN/USP/INN), Norvir (TN)
D10582	4.492(2)	3.595	Viekira pak (TN), Dasabuvir, ombitasvir, paritaprevir and ritonavir
D10745	4.492(2)	3.595	Technivie (TN), Ombitasvir, paritaprevir and ritonavir
D10558	4.511(2)	3.681	Umifenovir (INN)
D06458	4.195(2)	3.69	Gamma globulin (TN), Bay gam (TN), Human normal immunoglobulin (JP17
D08266	4.776(2)	3.741	Pizensy (TN), Lactitol (NF/INN), Importal (TN)
D03344	4.636(2)	3.774	Silver protein, mild, Silver protein (TN), Silver protein (JP17)
D10211	5.337(2)	3.857	Rotavirus vaccine, live, oral, pentavalent, Rotateq (TN)
D02134	4.898(2)	3.889	Azithromycin dihydrate, Zmax (TN), Azimycin (TN), Zithromac (TN), Az
D06390	4.898(2)	3.889	Azithromycin (USP), Zmax (TN)
D07486	4.898(2)	3.889	Azasite (TN), Azithromycin (TN), Azithromycin (INN)
D02366	4.804(2)	3.906	Chloroquine (USP/INN)
D03469	4.804(2)	3.906	Chloroquine hydrochloride (USP), Aralen hydrochloride (TN)
D12027	4.035(2)	5.796	Civet
D07422	4.506(2)	4.113	Fibrinolysin, human, Fibrinolysin (human) (INN), Fibrogammin (TN)
D03045	5.038(2)	4.132	Attapulgite, activated (USP), Parepectolin (TN)
D03251	5.038(2)	4.132	Medicinal carbon (JP17), Charcoal, activated (USP), Medicinal carbon
D10156	5.338(2)	4.196	Romosozumab (USAN), Evenity (TN), Romosozumab-aqqg, Romosozumab (gen
D10161	5.175(2)	4.217	Sarilumab (USAN), Kevzara (TN), Sarilumab (genetical recombination)
D08150	5.596(2)	4.39	Lufenuron (USP/INN), Program [veterinary] (TN)
D00003	5.621(2)	4.399	Oxygen (JP17/USP)
D03841	5.621(2)	4.399	Anesoxyn (TN), Nitrous oxide and oxygen
D04482	4.432(2)	5.803	Ethinylestradiol and levonorgestrel, Trivora (TN), Ange (TN), Tripha
D11936	5.042(2)	4.437	Bamlanivimab (USAN)
D09153	5.649(2)	4.44	Elder
D10308	5.125(2)	4.46	Baricitinib (JAN/USAN/INN), Olumiant (TN)
D02114	4.480(2)	5.47	Hydroxychloroquine sulfate (JAN/USP), Plaquenil (TN)
D04550	5.599(2)	4.486	Semilente iletin (TN), Insulin zinc, prompt (USP)
D01766	4.848(2)	4.497	Camostat monomethanesulfonate, Camostat mesilate (JP17), Camostat me
D05439	5.516(2)	4.57	Imagent (TN), Perflubron (USP/INN)
D02916	5.532(2)	4.572	Ammonia solution, strong (NF), Ammonia
D05765	5.532(2)	4.572	Rose water, strong (NF), Rose water, stronger (NF)
D07943	5.302(2)	4.594	Fendiline hydrochloride, Sensit (TN)
D07606	4.787(2)	4.6	Camostat (INN)
D03237	5.382(2)	4.635	Aluminum silicate, natural (JAN), Natural aluminum silicate (JP17),
D10553	4.639(3)	7.906	Dasabuvir (USAN/INN)
D10581	4.639(3)	7.906	Exviera (TN), Dasabuvir sodium hydrate, Dasabuvir sodium monohydrate
D03143	5.296(3)	4.643	Blood, whole (USP)
D11938	5.573(3)	4.647	Casirivimab (USAN)
D11939	5.573(3)	4.647	Imdevimab (USAN)
D02934	5.658(2)	4.658	Kineret (TN), Anakinra (USAN/INN)
D00366	4.662(2)	—	Lypressin (USAN/INN), Diapid (TN)

*Note*: Code: KEGG code, D_id: indirect (predicted) distance, D_d: direct (coexisting) distance.

**Fig. 2.**
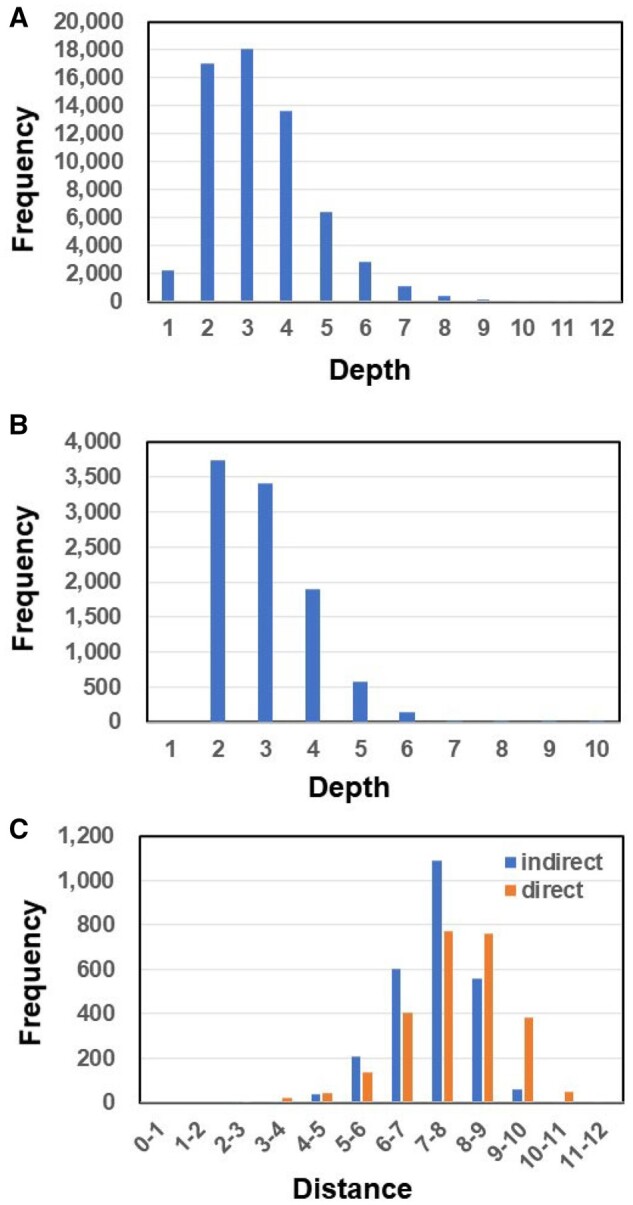
Statistics of the predicted distances of drugs by the Otsuka-Ochiai coefficient method. (**A**) The number of KEGG codes (nodes) connecting to COVID-19 at each depth (linkage number) from COVID-19 in the tree; in total, 61 997 nodes. (**B**) The number of drug codes (nodes) connecting to COVID-19 at each depth from COVID-19 in the tree; in total, 9799 nodes. (**C**) The number of drugs indirectly connected (concatenated by several KEGG codes) to COVID-19 in the tree and the number of drugs directly connected to COVID-19 at each distance; in total, 2558 nodes had both direct and indirect connections to COVID-19.

From the 98 556 KEGG codes with NAME/DEFINITION fields, 61 997 were connected to the tree as nodes at a depth of less than 12 (linkage numbers) (Fig. 2A). Of these, 9799 codes were drug codes (D codes) (Fig. 2B). In Table 1 and Supplementary Table S1, each KEGG code is listed according to its shorter distance to COVID-19 between the indirect and direct connections. Famous drug candidates (Singh *et al.*, 2020; Yousefi *et al.*, 2021) against COVID-19, such as remdesivir (KEGG: D11472), hydroxychloroquine (KEGG: D08050), favipiravir (KEGG: D09537), lopinavir (KEGG: D01425), umifenovir (KEGG: D10558), ritonavir (KEGG: D00427), and chloroquine (KEGG: D02366), were in the top part of the lists. Several codes were not directly connected to COVID-19. That is, there were no reports dealing with specific COVID-19 relationships, but some of them may be drug candidates for COVID-19. For the first 50 codes in this category, we checked all the connections in the path from COVID-19 to the respective drug. Considering that the internal KEGG–KEGG connections based on only one study are quite uncertain, we read the abstract and main text of all the literature existing in this situation and found five improper connections. Drugs identified by such improper connections are labeled by an asterisk in Supplementary Table S1 (they do not emerge in Table 1). However, the remaining drugs were selected through connecting codes concerning closely related disease and virus (SARS and SARS-CoV) proteins (enzymes), such as angiotensin-converting enzyme 2 and the main protease, and drugs that were presumably identified from enumerated descriptions of drugs in abstracts. We are currently testing the antiviral activities of promising drugs.

Some additional features of our prediction system should be noted. (i) Only approved drugs with KEGG D codes appear in this table, but compounds, such as intracellular metabolites (C code) and natural medicine (E code), also exist in the tree. These compounds can also be extracted as drug candidates. (ii) This method is based on word/phrase co-occurrence, so improper identification of relevance cannot be excluded. This improper identification can be propagated to later relationship inference steps. This inaccuracy can be compensated for by linking the output to the KEGG code and PubMed ID. One can assess the nature of substance/biological processes through KEGG codes and assess the plausibility of the prediction by accessing related reports through the PubMed IDs. As shown in Fig. 1B, our system connects each PubMed ID to KEGG codes/terms and each KEGG code/term to PubMed IDs. Based on these connections, relationships between KEGG codes/terms, such as similarity or connectivity, and relationships among PubMed IDs, such as similarity in dealing materials or phenomena, could be derived by using the dimensionality reduction method used in principal component analysis. Our system is planning to implement this approach.

Moreover, although this report focuses on COVID-19, the program developed here can be applied to other infectious diseases and to quickly identify drug candidates when new infectious diseases appear in the future.

## 4 Conclusion

We have never felt the speed and power of the propagation of a virus like we are currently experiencing with COVID-19. The development of new drugs takes time; therefore, many agree on the need for drug repositioning. Given that many possible actions that suppress the virus can be hypothesized, various assay methods must be used for drug screening. Many interactions between biomolecules have been reported in the human body. We can search for candidates among these biomolecules or connect information from multiple papers to infer candidates. This report is meant for that purpose.

We must stop the spread of SARS-CoV-2 now not only to reduce the current number of unfortunate deaths and critically ill patients. SARS-CoV-2 is currently accumulating genomic mutations. Since the outbreak of SARS-CoV-2 in December 2019, the most mutated strain has accumulated 39 mutations within 13 months, as seen on the Nextstrain (Hadfield *et al.*, 2018) web page (https://nextstrain.org/). Although the mutation rate, i.e. the accumulation rate, is constant, the number of simultaneous mutation experiments by the virus at once is dependent on the number of infections. Currently, 643 091 infections worldwide occur every 24 h (WHO, 2021) (as of May 13, 2021). The greater this number is, the more frequently mutation will occur that result in growth and infection advantages for the virus. Once the virus has gained this advantage, the mutation will become widespread, as noted in the case of the D614G mutation in the S protein, which occurred in January 2020, giving the virus an advantage in infectivity and greater life cycle processes (Hou *et al.*, 2020).

## Supplementary Material

vbab013_Supplementary_Data
