# Supplementary material for: A novel method of literature mining to identify candidate COVID-19 drugs
Source: Bioinform Adv. 2021 Jul 22;1(1):vbab013. doi: 10.1093/bioadv/vbab013 (PMC9710631; doi:10.1093/bioadv/vbab013)
Supplement: vbab013_Supplementary_Data [file vbab013_supplementary_data.zip › FigS1S2_muramatsu.docx]

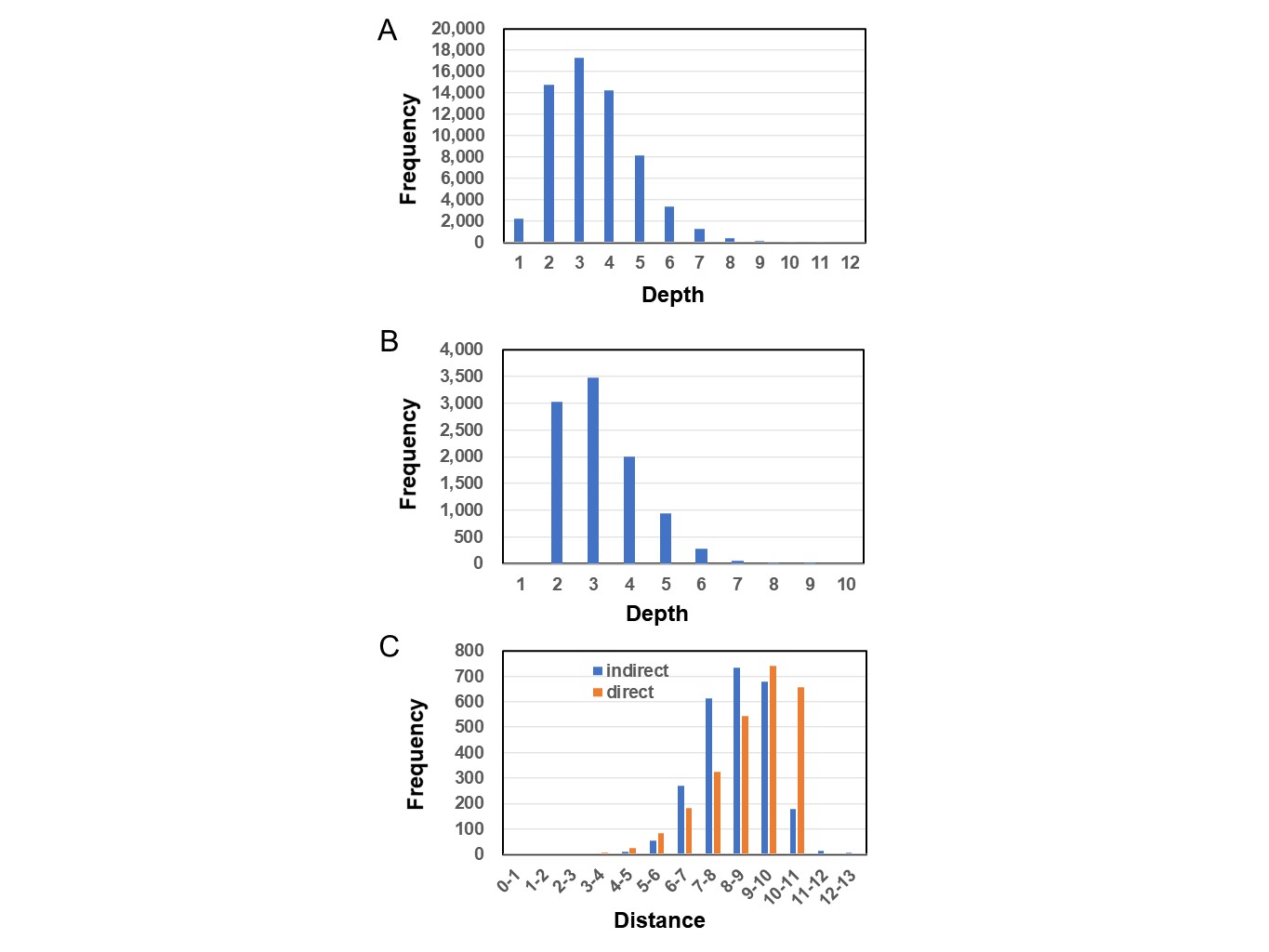


Fig. S1. Statistics of the predicted distances of drugs by the Dice index method. (A) The number of KEGG codes (nodes) connecting to COVID-19 at each depth (link-age number) from COVID-19 in the tree; in total, 61,997 nodes. (B) The number of drug codes (nodes) connecting to COVID-19 at each depth from COVID-19 in the tree; in total, 9,799 nodes. (C) The number of drugs indirectly connected (concatenated by sever-al KEGG codes) to COVID-19 in the tree and the number of drugs directly connected to COVID-19 at each distance; in total, 2,558 nodes had both direct and indirect connections to COVID-19.


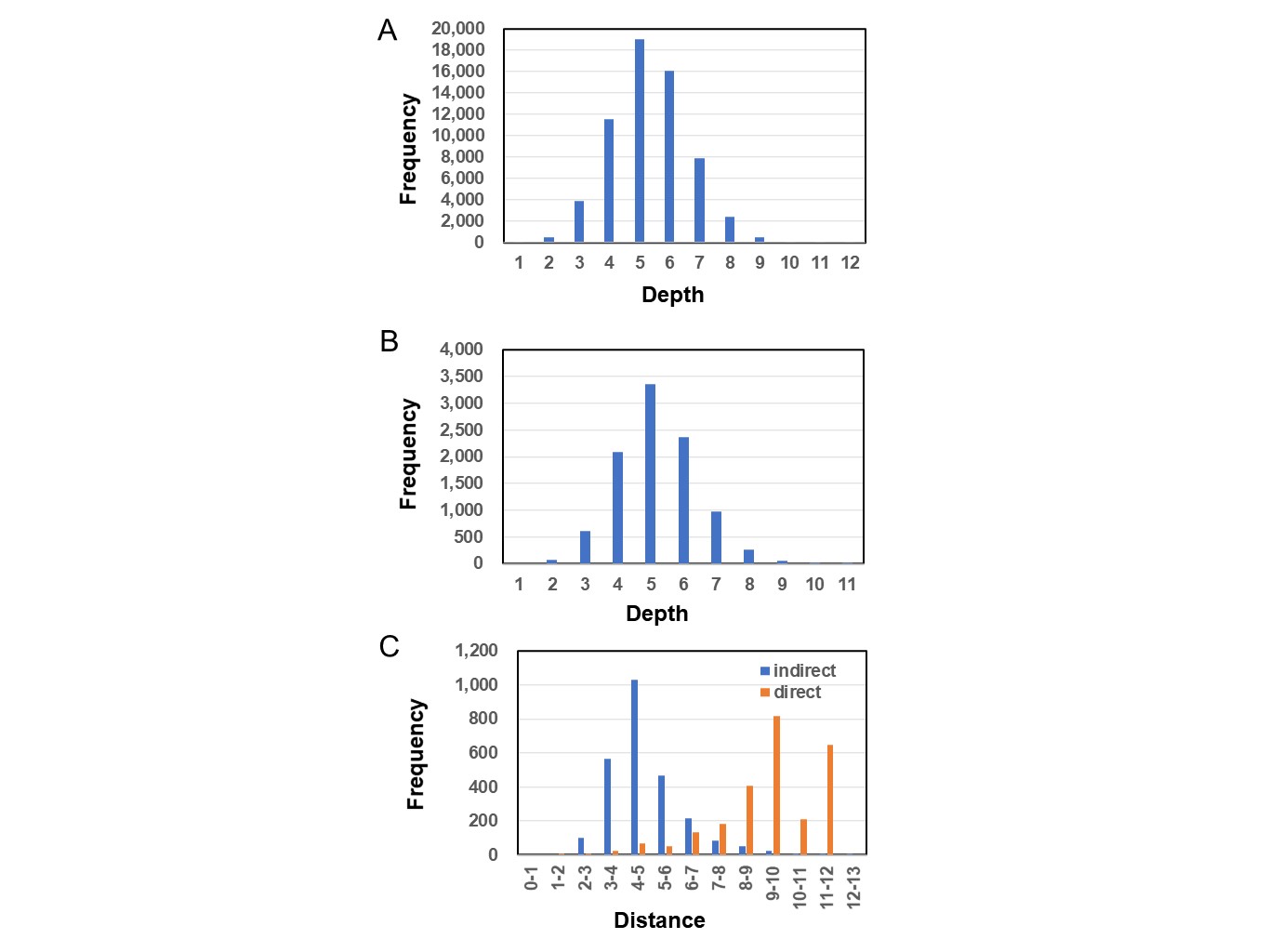


Fig. S2. Statistics of the predicted distances of drugs by the conditional probability method. (A) The number of KEGG codes (nodes) connecting to COVID-19 at each depth (link-age number) from COVID-19 in the tree; in total, 61,997 nodes. (B) The number of drug codes (nodes) connecting to COVID-19 at each depth from COVID-19 in the tree; in total, 9,799 nodes. (C) The number of drugs indirectly connected (concatenated by sever-al KEGG codes) to COVID-19 in the tree and the number of drugs directly connected to COVID-19 at each distance; in total, 2,558 nodes had both direct and indirect connections to COVID-19.
