# Supplementary material for: A novel method of literature mining to identify candidate COVID-19 drugs
Source: Bioinform Adv. 2021 Jul 22;1(1):vbab013. doi: 10.1093/bioadv/vbab013 (PMC9710631; doi:10.1093/bioadv/vbab013)
Supplement: vbab013_Supplementary_Data [file vbab013_supplementary_data.zip › TableS1_muramatsu.rtf]

Table S1. Distances between COVID-19 and each drug calculated by the Otsuka-Ochiai coefficient (cosine similarity) method (first 1000 items).
Code: KEGG code, D_id: indirect (predicted) distance, D_d: direct (co-existing) distance
(The five KEGG codes with an asterisk were determined to have been caused by an improper connection. See text.) 

Code    D_id(Dpt)  D_d   Name
---------------------------------------------------------------------------------------------------------------
D11472  2.939(2)  2.318  Veklury (TN), Remdesivir (JAN/USAN)
D08050  3.618(2)  2.607  Hydroxychloroquine (INN), Polirreumin (TN)
D09537  3.802(2)  3.137  Favipiravir (JAN/USAN/INN), Avigan (TN)
D01425  4.060(2)  3.191  Lopinavir (JAN/USP/INN)
D02498  4.060(2)  3.191  Lopinavir and ritonavir, Kaletra (TN)
D02596  4.451(2)  3.242  Tocilizumab (genetical recombination) (JAN), Tocilizumab (USAN/INN), Actemra (TN)
D03043  4.766(2)  3.307  Air (TN), Air, medical (USP)
D05864  4.820(2)  3.339  Sodium monofluorophosphate (USP), Aim (TN)
D00427  4.492(2)  3.595  Ritonavir (JAN/USP/INN), Norvir (TN)
D10582  4.492(2)  3.595  Viekira pak (TN), Dasabuvir, ombitasvir, paritaprevir and ritonavir
D10745  4.492(2)  3.595  Technivie (TN), Ombitasvir, paritaprevir and ritonavir
D10558  4.511(2)  3.681  Umifenovir (INN)
D06458  4.195(2)  3.690  Gamma globulin (TN), Bay gam (TN), Human normal immunoglobulin (JP17), Hyqvia (TN), Globulin, immune (USP)
D08266  4.776(2)  3.741  Pizensy (TN), Lactitol (NF/INN), Importal (TN)
D03344  4.636(2)  3.774  Silver protein, mild, Silver protein (TN), Silver protein (JP17)
D10211  5.337(2)  3.857  Rotavirus vaccine, live, oral, pentavalent, Rotateq (TN)
D02134  4.898(2)  3.889  Azithromycin dihydrate, Zmax (TN), Azimycin (TN), Zithromac (TN), Azithromycin hydrate (JP17)
D06390  4.898(2)  3.889  Azithromycin (USP), Zmax (TN)
D07486  4.898(2)  3.889  Azasite (TN), Azithromycin (TN), Azithromycin (INN)
D02366  4.804(2)  3.906  Chloroquine (USP/INN)
D03469  4.804(2)  3.906  Chloroquine hydrochloride (USP), Aralen hydrochloride (TN)
D12027  4.035(2)  5.796  Civet
D07422  4.506(2)  4.113  Fibrinolysin, human, Fibrinolysin (human) (INN), Fibrogammin (TN)
D03045  5.038(2)  4.132  Attapulgite, activated (USP), Parepectolin (TN)
D03251  5.038(2)  4.132  Medicinal carbon (JP17), Charcoal, activated (USP), Medicinal carbon (TN)
D10156  5.338(2)  4.196  Romosozumab (USAN), Evenity (TN), Romosozumab-aqqg, Romosozumab (genetical recombination) (JAN)
D10161  5.175(2)  4.217  Sarilumab (USAN), Kevzara (TN), Sarilumab (genetical receombination) (JAN)
D08150  5.596(2)  4.390  Lufenuron (USP/INN), Program [veterinary] (TN)
D00003  5.621(2)  4.399  Oxygen (JP17/USP)
D03841  5.621(2)  4.399  Anesoxyn (TN), Nitrous oxide and oxygen
D04482  4.432(2)  5.803  Ethinylestradiol and levonorgestrel, Trivora (TN), Ange (TN), Triphasil-21 (TN), Levora (TN), Seasonique (TN), Lo/ovral (TN)
D11936  5.042(2)  4.437  Bamlanivimab (USAN)
D09153  5.649(2)  4.440  Elder
D10308  5.125(2)  4.460  Baricitinib (JAN/USAN/INN), Olumiant (TN)
D02114  4.480(2)  5.470  Hydroxychloroquine sulfate (JAN/USP), Plaquenil (TN)
D04550  5.599(2)  4.486  Semilente iletin (TN), Insulin zinc, prompt (USP)
D01766  4.848(2)  4.497  Camostat monomethanesulfonate, Camostat mesilate (JP17), Camostat mesylate, Foipan (TN)
D05439  5.516(2)  4.570  Imagent (TN), Perflubron (USP/INN)
D02916  5.532(2)  4.572  Ammonia solution, strong (NF), Ammonia
D05765  5.532(2)  4.572  Rose water, strong (NF), Rose water , stronger (NF)
D07943  5.302(2)  4.594  Fendiline hydrochloride, Sensit (TN)
D07606  4.787(2)  4.600  Camostat (INN)
D03237  5.382(2)  4.635  Aluminum silicate, natural (JAN), Natural aluminum silicate (JP17), Adsorbin (TN)
D10553  4.639(3)  7.906  Dasabuvir (USAN/INN)
D10581  4.639(3)  7.906  Exviera (TN), Dasabuvir sodium hydrate, Dasabuvir sodium monohydrate
D03143  5.296(3)  4.643  Blood, whole (USP)
D11938  5.573(3)  4.647  Casirivimab (USAN)
D11939  5.573(3)  4.647  Imdevimab (USAN)
D02934  5.658(2)  4.658  Kineret (TN), Anakinra (USAN/INN)
D00366  4.662(2)    -    Lypressin (USAN/INN), Diapid (TN)
D06505  6.263(2)  4.733  Starch, topical (USP)
D11121  5.479(2)  4.765  Gimsilumab (USAN/INN)
D02844  5.905(2)  4.776  Basaljel (TN), Aluminum carbonate, basic (USAN)
D03678  5.905(2)  4.776  Fuchsin, basic (USP)
D11236  5.902(2)  4.783  Lenzilumab (USAN/INN)
D10598  4.786(4)    -    Ombitasvir heminonahydrate, Ombitasvir hydrate (JAN)
D10576  4.786(4)    -    Ombitasvir (USAN)
D07471  4.786(2)  6.087  Atazanavir (INN), Reyataz (TN)
D10753  4.786(2)  5.434  Atazanavir and cobicistat, Evotaz (TN)
D04548  4.808(2)  4.805  Insulin, neutral (USAN), Neutral insulin injection (INN)
D04319  5.999(2)  4.806  Glaze, pharmaceutical (NF)
D00429  4.831(2)  6.560  Fortovase (TN), Saquinavir (JAN/USP/INN)
D10580  4.836(4)  6.842  Paritaprevir (USAN/INN)
D10597  4.836(4)  6.842  Paritaprevir hydrate (JAN), Paritaprevir dihydrate
D03656  4.865(2)  5.218  Darunavir (USAN/INN)
D10832  4.865(2)  5.218  Prezcobix (TN), Darunavir and cobicistat, Rezolsta (TN)
D11382  4.865(2)  5.218  Symtuza (TN), Darunavir, cobicistat, emtricitabine and tenofovir alafenamide
D08802  4.868(3)  5.379  Human blood-coagulation factor XIII fraction, dried, Fibrogammin P (TN)
D02685  5.623(2)  4.909  Seperidol hydrochloride (USAN)
D05848  5.623(2)  4.909  Eleutheroside A, Sitogluside (USAN/INN), Daucosterol, beta-Sitosterol glucoside
D11944  5.623(2)  4.909  Etesevimab (USAN)
D04969  6.133(3)  4.923  Wax, white (NF), White beeswax (JP17), White wax (TN)
D05239  6.133(3)  4.923  White ointment (JP17), Ointment, white (USP)
D05304  6.133(3)  4.923  White petrolatum (JP17), Petrolatum, white (USP), Moroline (TN)
D09669  5.818(2)  4.925  Sylvant (TN), Siltuximab (USAN/INN)
D02125  5.894(2)  4.964  Aralen (TN), Chloroquine phosphate (USP)
D00548  6.958(2)  4.966  Etomidate (USP/INN), Amidate (TN)
D04664  5.892(2)  4.968  Lanolin (TN), Wool wax, Lanolin, modified (USP), Hydrous lanolin (JP17)
D08240  5.325(3)  4.989  Nafamostat (INN)
D03833  5.022(2)  7.066  Indinavir (USAN), Indinavir hydrate
D08306  5.745(2)  5.037  Oseltamivir (INN), Agucort (TN)
D05258  5.433(3)  5.045  Anteron (TN), Serum gonadotrophin (JAN), Gonadotrophin, serum (JAN/INN)
D05043  6.067(2)  5.055  Mineral oil, light (NF), Light liquid paraffin (JP17)
D04513  5.062(2)    -    Imidurea (NF)
D00150  5.063(2)  5.397  Angiotensin II (INN)
D02014  5.063(2)  5.397  Angiotensin II (human type) (JAN), Angiotensin II (USAN), Delivert (TN)
D06544  5.941(2)  5.087  Corticotropin, repository (USP), Cortigel (TN)
D00357  5.089(2)  7.061  Losartan potassium (JP17/USP), Cozaar (TN)
D07895  5.089(2)  7.061  Hyzaar (TN), Losartan potassium and hydrochlorothiazide (JP17), Hydrochlorothiazide and losartan potassium, Losarhyd (TN)
D08146  5.089(2)  7.061  Losartic (TN), Losartan (INN)
D11399  5.736(2)  5.093  Leronlimab (USAN/INN)
D01670  5.157(2)  5.102  Nafamostat mesylate (USAN), Ronastat (TN), Nafamostat mesilate (JP17)
D10226  5.112(2)  7.499  Sacubitril mixture with valsartan, Sacubitril valsartan sodium hydrate (JAN), Entresto (TN)
D00111  5.281(2)  5.112  Lactate (TN), Lactic acid (JP17/USP)
D00936  5.281(2)  5.112  Prequist powder (TN), Calcium lactate (USP)
D02183  5.281(2)  5.112  Sodium lactate (JAN/USP), Mediject L (TN)
D02254  5.281(2)  5.112  Calcium lactate (TN), Calcium lactate pentahydrate, Calcium lactate hydrate (JP17)
D00448  5.112(2)  7.160  Sulfasalazine (USP/INN), Salazosulfapyridine (JP17), Azulfidine (TN)
D04543  6.066(2)  5.115  Insulin human zinc, extended (USP)
D04549  6.066(2)  5.115  Ultralente iletin (TN), Insulin zinc, extended (USP)
D11581  5.133(3)  7.593  Perindopril and amlodipine, Prestalia (TN)
D03343  5.149(3)  7.393  MDS (TN), Dextran sulfate sodium sulfur 5 (JP17), Dextran sulfate sodium sulfur 18 (JP17)
D10528  5.154(2)  5.411  Aldosterone (INN)
D07508  5.722(2)  5.155  Mediator (TN), Benfluorex hydrochloride
D03138  6.481(2)  5.160  Blood cells, red (USP)
D03063  6.097(2)  5.176  BCG vaccine (USP), Tice BCG (TN)
D08259  5.191(2)  5.958  Viracept (TN), Nelfinavir (INN)
D02486  5.647(2)  5.194  Alinia (TN), Nitazoxanide (USAN/INN)
D11838  6.186(2)  5.206  Vilobelimab (USAN)
D02112  5.968(2)  5.210  Heparin sodium (JP17/USP/INN), Parnaparin sodium (INN), Liquemin sodium (TN)
D04427  5.968(2)  5.210  Calciparin, Calciparine (TN), Heparin calcium (JP17), Nadroparin calcium (INN)
D07510  5.968(2)  5.210  Heparin (TN), Heparin (BAN), Adomiparin (USAN), Semuloparin (USAN), Nadroparin, Enoxaparin, Bemiparin, Parnaparin
D02050  5.211(2)  7.492  Potassium phosphate, monobasic (JAN/NF)
D04400  5.211(2)  7.492  Sodium phosphate, monobasic (USP), Anhydrous monobasic sodium phosphate
D08741  5.211(2)  6.007  Karmi CA (TN), Adenine, sodium citrate hydrate, citric acid hydrate, glucose and potassium phosphate, monobasic, CPDA solution
D08742  5.211(2)  6.007  CPD solution, Sodium citrate hydrate, citric acid hydrate, glucose and potassium phosphate, monobasic, Karmi C (TN)
D08743  5.211(2)  6.007  MAP solution, D-Mannitol, adenine, potassium phosphate, monobasic, sodium citrate hydrate, citric acid hydrate, glucose and sodium chloride
D00584  5.214(4) 10.076  Fluoroplex (TN), 5-FU (TN), Adrucil (TN), Carac (TN), Fluorouracil (JP17/USP/INN)
D04371  6.626(2)  5.230  Soap, green (USP), Green soap
D00407  6.000(2)  5.241  Medrol (TN), Methylprednisolone (JP17/USP/INN)
D04250  6.000(2)  5.241  Fradiomycin sulfate and methylprednisolone, Neo medrol EE (TN)
D09959  6.401(2)  5.253  Ruxolitinib (USAN/INN)
D02974  5.969(2)  5.255  Aranotin (USAN/INN)
D09353  5.493(2)  5.255  L-5-Methyltetrahydrofolic acid, Metafolin (TN), Levomefolinic acid, Levomefolic acid (USAN/INN)
D01703  6.196(2)  5.266  Omnaris (TN), Zetonna (TN), Alvesco (TN), Ciclesonide (JAN/USAN/INN)
D05355  5.268(2)  5.720  Papain (USP), Caroid (TN)
D00292  6.237(2)  5.271  Maxidex (TN), Decadron (TN), Dexamethasone (JP17/USP/INN)
D02273  6.237(2)  5.271  Isoproterenol sulfate, dexamethasone and atropine methybromide, Stmerin D (TN)
D08733  6.237(2)  5.271  Glymesason (TN), Dexamethasone and glyteer
D10296  6.237(2)  5.271  Ciprodex (TN), Ciprofloxacin hydrochloride and dexamethasone
D11177  6.237(2)  5.271  Dexamethasone, neomycin sulfate and polymyxin B sulfate, Dexasporin (TN), Maxitrol (TN)
D11178  6.237(2)  5.271  Tobramycin and dexamethasone, Tobradex (TN)
D08682  5.721(2)  5.273  Choice (TN), Warfarin (INN), Warfarine
D10193  5.882(3)  5.285  Rotarix (TN), Live attenuated human rota virus vaccine, oral
D04182  6.471(2)  5.304  Fibrinogen (125I) (INN), Ibrin (TN), Fibrinogen I 125 (USAN)
D00804  5.899(2)  5.317  Ivermectin (JAN/USP/INN), Stromectol (TN), Soolantra (TN), Sklice (TN)
D08095�– 5.336(3)    -    Corlanor (TN), Coralan (TN), Ivabrandine hydrochloride, Corlentor (TN), Ivabradine hydrochloride (JAN/USAN)
D00423  5.387(2)  5.338  Ribavirin (JP17/USP/INN), Copegus (TN), Virazole (TN), Ribasphere (TN), Rebetol (TN)
D11032  5.347(2)  6.287  Plitidepsin (INN)
D05218  6.103(2)  5.364  Ocrelizumab (genetical recombination) (JAN), Ocrevus (TN), Ocrelizumab (USAN)
D01732  6.183(2)  5.379  Anhydous sodium sulfate (JP17), Sodium sulfate, dried (JAN), Sodium sulfate, dried (TN)
D02416  6.183(2)  5.379  ALterna GEL (TN), Dialume (TN), Aluminum hydroxide, dried (USP), Dried aluminum hydroxide gel (JP17), Aluminum hydroxide (USP)
D04172  6.183(2)  5.379  Fero-gradumet (TN), Tetucur (TN), Ferrous sulfate, dried (USP), Ferrous sulfate hydrate
D04364  6.010(2)  5.379  Kolantyl (TN), Dicyclomine hydrochloride, aluminium hydroxide, dried and magnesium oxide
D04380  6.183(2)  5.379  Ebios (TN), Dried yeast (JP17), Yeast, dried
D05283  6.183(2)  5.379  Sodium carbonate, dried, Dried sodium carbonate (JP17), Sodium carbonate (NF)
D08708  6.183(2)  5.379  AM (TN), NS (TN), Licase (alpha Amylase), aluminum hydroxide, dried, magnesium carbonate, sodium bicarbonate and precipitated calcium carbonate
D08793  6.183(2)  5.379  Human blood-coagulation factor eight inhibitor bypassing activity complex, dried, Feiba (TN)
D08794  6.183(2)  5.379  PPSB-HT (TN), Human blood-coagulation factor IX complex, dried
D08816  6.183(2)  5.379  Niflec (TN), Potassium chloride, sodium chloride, sodium bicarbonate and sodium sulfate, dried
D00251  5.380(2)  8.090  Apopril (TN), Capoten (TN), Captopril (JP17/USP/INN)
D10276  5.380(2)  8.090  Captopril and hydrochlorothiazide, Capozide (TN)
D00894  5.381(2)  6.873  Agenerase (TN), Amprenavir (JAN/USAN/INN)
D04296  6.684(2)  5.384  Diprophylline, methoxyphenamine hydrochloride, noscapine and chlorpheniramine maleate, Asthma (TN)
D07227  5.407(2)    -    Ornipressin (INN), POR 8 Sandoz (TN)
D11971 11.415(2)  5.409  Tozinameran (JAN), Comirnaty (TN)
D01202  6.380(2)  5.431  Artes (TN), Melinamide (JAN)
D09881  5.842(2)  5.434  Tybost (TN), Cobicistat (JAN/USAN/INN)
D10755  5.624(2)  5.434  Genvoya (TN), Elvitegravir, cobicistat, emtricitabine and tenofovir alafenamide
D10756  5.568(3)  5.434  Stribild (TN), Elvitegravir, cobicistat, emtricitabine and tenofovir disoproxil
D00952  8.042(2)  5.453  Megestrol acetate (USP), Megace (TN)
D00002  5.696(2)  5.458  Nicotinamide adenine dinucleotide, Nadide (JAN/USAN/INN)
D06719  6.389(2)  5.458  Powdered cyperus rhizome (JP17), Cyperus rhizome (JP17), Xianghu (TN)
D10166    -       5.458  Sovaprevir (USAN)
D06074  5.463(3)  6.457  Tenofovir (USAN), Tenofovir hydrate
D06298  6.315(3)  5.488  Armes (TN), Vidarabine (JAN), Vidarabine anhydrous, ARA-A
D06677  5.492(3)    -    GS-9137, Elvitegravir (JAN/USAN), Vitekta (TN)
D00896  5.508(2)  8.312  Efavirenz (JAN/USP/INN), Sustiva (TN)
D10851  5.508(2)  7.135  Efavirenz, emtricitabine and tenofovir disoproxil, Atripla (TN)
D11392  5.508(2)  7.572  Symfi (TN), Efavirenz, lamivudine and tenofovir disoproxil fumarate
D00085  5.509(3)  6.541  Insulin (JAN/USP)
D04546  5.509(3)  6.541  Insulin, dalanated (USAN)
D04547  5.509(3)  6.541  Isophane insulin human (genetical recombination) injectable aqueous suspension (JP17), Insulin, isophane (USP), Isophane insulin (aqueous suspension) (JAN), Humalog PEN (TN)
D00028  5.706(2)  5.510  Concentrated glycerin (JP17), Glycerin, concentrated (JAN), Glycerol (INN), Glycerin (JP17/USP)
D08780  5.706(2)  5.510  Berinert P (TN), Human C1 inactivator, freeze-dried concentrated
D08795  5.706(2)  5.510  Neuart (TN), Human anti-thrombin III, freeze-dried concentrated
D08796  5.706(2)  5.510  Anact C (TN), Human activated protein C, freeze-dried concentrated
D10884  5.706(2)  5.510  Human prothrombin complex, freeze-dried concentrated
D11965  5.706(2)  5.510  Human alpha 1-Proteinase inibitor, freeze-dried concentrated, Lynspad (TN), alpha 1-Antitrypsin
D00534  6.725(2)  5.512  Ovide (TN), Malathion (USP)
D02305  5.516(2)  7.150  Roxadyl (TN), Rosoxacin (USAN/INN), ROS
D00142  5.528(2)  7.035  Xatmep (TN), Otrexup (TN), Methotrexate (JP17/USP/INN)
D02115  5.528(2)  7.035  Methotrexate sodium, Rasuvo (TN), Trexall (TN)
D03674  6.531(2)  5.536  Lovenox (TN), Enoxaparin sodium (JAN/USP/INN)
D01494  6.006(2)  5.553  Hyperband (TN), Paraformaldehyde (JP17)
D02598  6.747(2)  5.553  Avsola (TN), Inflectra (TN), Infliximab (USAN/INN), Renflexis (TN), Infliximab-dyyb, Infliximab-axxq, Infliximab-abda, Infliximab (genetical recombination) [Infliximab biosimilar 1] (JAN), Infliximab (genetical recombination) [Infliximab biosimilar 3] (JAN), Infliximab (genetical recombination) (JAN), Infliximab (genetical recombination) [Infliximab biosimilar 2] (JAN), Remicade (TN)
D06986  5.623(2)  5.553  Shosaikotokakikyosekko
D11187  5.839(2)  5.553  Dociparstat sodium (USAN/INN)
D11837  5.623(2)  5.553  Zotatifin (USAN/INN)
D04133  6.024(3)  5.556  Fat, hard (NF)
D00068  6.248(3)  5.557  Alcohol (USP), Dehydrated ethanol (TN), Anhydrous ethanol (JP17), Dehydrated ethanol, Ethyl alcohol
D02799  6.248(3)  5.557  Alcolo (TN), Alcohol, rubbing (USP)
D04732  6.248(3)  5.557  Isopropanol and methylated alcohol, Alcohol (TN)
D10301  6.248(3)  5.557  Prevantics (TN), Chlorhexidine gluconate and alcohol
D01982  5.568(3)  7.572  Viread (TN), Tenofovir disoproxil fumarate (JAN/USAN)
D02297  5.568(3)  7.135  Emtricitabine and tenofovir disoproxil, Truvada (TN)
D07892  5.578(2)  8.816  Enalapril (TN), Enalapril (INN)
D03600  6.057(2)  5.587  Creatinine (NF)
D09578  5.595(2)  6.018  Albumin human, recombinant (NF)
D05344    -       5.602  Pamaqueside (USAN/INN)
D03843  5.603(2)  7.404  Tipranavir disodium (USAN)
D08605  5.603(2)  7.404  Tipranavir (INN), Aptivus (TN)
D11438  5.892(2)  5.605  Bemcentinib (USAN/INN)
D01728  7.289(2)  5.609  Gypsum fibrosum (TN), Gypsum (JP17)
D06773  6.365(2)  5.622  Lonicera leaf and stem (JP17)
D04725  5.623(2)    -    Lexithromycin (USAN/INN)
D01199  5.624(2)  7.135  Emtricitabine (JAN/USAN/INN), Emtriva (TN)
D10571  5.624(2)  7.135  Rilpivirine hydrochloride, tenofovir disoproxil fumarate and emtricitabine, Complera (TN), Eviplera (TN)
D10835  5.624(2)  6.670  Emtricitabine and tenofovir alafenamide, Descovy (TN)
D10836  5.624(2)  6.670  Odefsey (TN), Emtricitabine, rilpivirine and tenofovir alafenamide
D11039  5.624(2)  6.670  Bictegravir, emtricitabine and tenofovir alafenamide, Biktarvy (TN)
D11395  5.629(4)  7.345  Lamivudine and tenofovir disoproxil, Temixys (TN), Cimduo (TN)
D11396  5.629(4)  7.345  Delstrigo (TN), Doravirine, lamivudine and tenofovir disoproxil
D00831  5.631(2)  6.839  Mefloquine hydrochloride (JP17/USP), Lariam (TN)
D04895  5.631(2)  6.839  Mefloquine (USAN/INN)
D10600  5.640(3)  7.395  Dolutegravir, abacavir and lamivudine, Triumeq (TN)
D00016  5.799(2)  5.649  L-Serine (JP17), Serine (USP)
D00488  5.663(2)  8.646  Pyrimethamine (JAN/USP/INN), Daraprim (TN)
D02448  5.663(2)  8.646  Pyrimethamine and sulfadoxine, Fansidar (TN)
D00276  5.666(2)  7.036  Biaxin (TN), Clarithromycin (JP17/USP/INN)
D08774  5.666(2)  7.036  Amoxicillin hydrate, clarithromycin and lansoprazole, Lansap (TN), Prevpac (TN)
D10246  5.666(2)  7.036  Omeclamox-pak (TN), Omeprazole, clarithromycin and amoxicillin
D10519  5.666(2)  7.036  Rabeprazole, amoxicillin and clarithromycin, Rabecure (TN)
D10775  5.666(2)  7.036  Vonoprazan, amoxicillin and clarithromycin, Vonosap (TN)
D02026  5.888(2)  5.672  Magnesium aspartate, Magnesium L-aspartate (JAN)
D04948  5.888(2)  5.672  Potassium aspartate, Potassium L-aspartate (JAN), L-Aspartate potassium, Aspara K (TN)
D04952  5.888(2)  5.672  Aspara (TN), Potassium aspartate and magnesium aspartate (JAN/USAN)
D02497  5.682(2)  7.123  Fosamprenavir (INN)
D02867  5.682(2)  7.123  Fosamprenavir calcium hydrate (JAN), Lexiva (TN)
D03835  5.682(2)  7.123  Fosamprenavir calcium (USAN), Lexiva (TN), Telzir (TN)
D03837  5.682(2)  7.123  Fosamprenavir sodium (USAN)
D10779  5.682(3)  9.405  Zavicefta (TN), Ceftazidime and avibactam, Avycaz (TN)
D00749  5.686(2)  6.943  Arava (TN), Leflunomide (JAN/USP/INN)
D01505  5.854(3)  5.698  Domin (TN), Talipexole hydrochloride (JAN)
D08446  5.854(3)  5.698  Dominal (TN), Prothipendyl hydrochloride
D00897  5.708(3)    -    Indinavir sulfate (USP), Crixivan (TN)
D02861  5.708(3)    -    Crixivan (TN), Indinavir sulfate ethanolate (JAN)
D09971  5.734(2)  5.714  Tegobuvir (USAN/INN)
D00564  5.721(2)  7.012  Warfarin sodium (USP), Coumadin (TN), Jantoven (TN)
D01280  5.721(2)  7.012  Warfarin potassium (JP17), Athrombin-K (TN)
D00012  5.890(2)  5.723  Alanine (USP), L-Alanine (JP17)
D02769  5.740(5)  9.998  Adenyl (TN), Adenosine phosphate (USAN/INN)
D00701  5.743(2)  7.797  Luminal sodium (TN), Phenobarbital sodium (JAN/USP/INN)
D00522  5.746(2)  7.529  Candesartan (USAN/INN)
D02679  6.042(2)  5.755  Piperacetazine (USAN/INN), Quide (TN)
D09138  6.141(2)  5.755  Aster root and rhizome, Aster root (Non-JPS), Asteris radix
D00036  5.761(2)  8.241  Niacinamide (USP), Nicotinamide (JP17/INN)
D04252  5.761(2)  8.241  Stomin A (TN), Nicotinamide and papaverine hydrochloride
D04915  5.761(2)  8.241  Pancal (TN), Calcium pantothenate, riboflavin, pyridoxine hydrochloride and nicotinamide
D07851  5.761(2)  7.075  C para (TN), Ascorbic acid, thiamine chloride hydrochloride, pyridoxine hydrochloride, riboflavin sodium phosphate, nicotinamide and panthenol
D07859  5.761(2)  6.969  Wasser-V (TN), Ascorbic acid, thiamine nitrate, nicotinamide, calcium pantothenate, pyridoxine hydrochloride and riboflavin
D08830  5.761(2)  6.969  Retinol palmitate, thiamine nitrate, riboflavin, pyridoxine hydrochloride, cyanocobalamin, ascorbic acid, ergocalciferol, tocopherol acetate, calcium pantothenate, nicotinamide and folic acid, Panvitan (TN)
D01778  6.266(2)  5.766  Bisolvon (TN), Bromhexine hydrochloride (JP17/USAN)
D06676  5.774(2)  6.959  MK-0518, Raltegravir (INN)
D07133  5.774(2)  6.959  Raltegravir potassium (JAN/USAN), Isentress (TN)
D10754  5.774(2)  6.959  Lamivudine and raltegravir, Dutrebis (TN), Lamivudine and raltegravir potassium
D10105  5.775(3)  6.441  Daclatasvir dihydrochloride (USAN), Daclatasvir hydrochloride (JAN), Daklinza (TN)
D02577  5.775(3)    -    Flibanserin (USAN/INN), Addyi (TN)
D07680  6.517(2)  5.776  Chloroquine sulfate, Nivaquine (TN)
D00009  5.778(3)  6.007  D-Glucose, Glucose (JP17), Purified glucose (JP17)
D02019  5.778(3)  6.007  Magnesol (TN), Magnesium sulfate hydrate and glucose
D02325  5.778(3)  6.007  Dextrose monohydrate, Dextrose (USP), alpha-D-Glucose monohydrate, Glucose hydrate (JP17), Cartose (TN)
D04109  5.778(3)  6.007  Glucose and inorganic salt, Opeguard MA (TN)
D04337  5.778(3)  6.007  Glucose-40 (TN), Glucose, liquid (JAN/NF), Liquid glucose
D04963  5.778(3)  6.007  Dextran 40 and glucose, Dextron (TN)
D04978  5.778(3)  6.007  ACD-A (TN), Acid -Citrate -Dextrose solution, Sodium citrate hydrate, citric acid hydrate and glucose, ACD-A solution
D01160  5.783(3)  6.592  Saquinavir mesylate (USP), Saquinavir mesilate (JAN), Invirase (TN)
D11857  5.796(3)  7.687  Duaklir pressair (TN), Aclidinium bromide and formoterol fumarate
D04674  8.831(3)  5.805  Forsythia fruit (JP17), Powdered forsythia fruit (Non-JPS), Forsythia fruit (TN)
D10658  6.519(2)  5.805  Ralimetinib (USAN/INN)
D02466  5.809(2)  7.435  Camoquin hydrochloride (TN), Amodiaquine hydrochloride (USP)
D02922  5.809(2)  7.435  Amodiaquine (USP/INN)
D09025  5.810(3)  7.673  Triglycerides, medium-chain (NF)
D10578  5.812(3)  5.931  Harvoni (TN), Ledipasvir and sofosbuvir
D09930  7.487(2)  5.814  Mavrilimumab (USAN/INN)
D00580  5.822(2)  9.352  Sulfadoxine (JAN/USP/INN)
D00040  5.825(3)  7.103  Cholesterol (TN), Cholesterol (JP17/NF)
D05424  6.622(3)  5.826  Human serum albumin, Albumin (TN), Human serum albumin (genetical recombination) (JAN)
D00238  5.828(2)  7.274  Azathioprine (JP17/USP/INN), Azasan (TN), Imuran (TN)
D03033  5.828(2)  7.274  Azathioprine sodium (USP), Imuran (TN)
D00753  5.832(4)  7.027  Rapamycin (TN), Sirolimus (JAN/USAN/INN), Rapamune (TN)
D02830  6.063(2)  5.833  Alovudine (USAN/INN)
D01738  6.758(2)  5.839  Definity (TN), Perflutren (JAN/USAN/INN)
D09002  5.840(2)    -    Sodium sulfite (NF), Dried sodium sulfite (JP17)
D07974  5.846(4) 10.076  Fluorouracil (TN), Fluorouracil sodium salt
D00400  5.862(2)  7.847  Diovan (TN), Valsartan (JP17/USP/INN)
D09197  5.862(2)  7.847  Valsartan and hydrochlorothiazide (JP17), Co-dio (TN)
D09745  5.862(2)  7.847  Exforge (TN), Valsartan and amlodipine besilate
D10286  5.862(2)  7.593  Exforge hct (TN), Amlodipine, valsartan and hydrochlorothiazide
D10287  5.862(2)  7.847  Valturna (TN), Aliskiren hemifumarate and valsartan
D10525  5.862(2)  7.847  Atedio (TN), Valsartan and cilnidipine
D11388  5.862(2)  7.847  Nebivolol and valsartan, Byvalson (TN)
D03304  6.407(2)  5.864  Feron (TN), Interferon beta (JAN)
D00517  5.865(2)  6.336  Aspiral (TN), Vaporole (TN), Amyl nitrite (JP17/USP)
D08914  5.866(2)    -    Eribulin mesilate (JAN), Eribulin mesylate (USAN), Halaven (TN)
D01035  6.393(2)  5.868  Cepharanthine (JAN), Cepharanthine (TN)
D01038  5.873(2)  6.480  Hesperidin (JAN)
D10657  5.877(3)    -    Pradigastat sodium (USAN)
D10664  5.877(3)    -    Pradigastat (USAN)
D06906  6.812(2)  5.882  Cicadae periostracum, Zentai, Cicada larva exuvia, Cicada slough (Non-JPS)
D08547  6.269(3)  5.885  Sulodexide (INN), Vessel (TN)
D09546*  5.885(3)    -    Edoxaban tosilate hydrate (JAN), Edoxaban tosylate monohydrate, Lixiana (TN), Savaysa (TN)
D10165  5.892(2)  6.011  Setrobuvir (USAN)
D07542  6.266(2)  5.892  Fluibron (TN), Bromhexine (INN)
D00353  5.896(2)  7.960  Lamivudine (JAN/USP/INN), Epivir (TN)
D07507  5.896(2)  6.789  Zidovudine and lamivudine, Combivir (TN), Lamivudine zidovudine (TN)
D08775  5.896(2)  7.960  Abacavir sulfate and lamivudine, Epzicom (TN)
D10838  5.896(2)  7.954  Trizivir (TN), Abacavir, lamivudine and zidovudine
D11521  5.896(2)  7.960  Lamivudine, nevirapine and zidovudine
D11522  5.896(2)  7.395  Dovato (TN), Dolutegravir and lamivudine
D10442  5.898(3)  7.567  Ledipasvir (USAN)
D00501  7.845(2)  5.899  Pentoxifylline (JAN/USP/INN), Trental (TN), Pentoxil (TN)
D11873  8.824(2)  5.899  Rilzabrutinib (USAN/INN)
D11943  5.969(2)  5.899  Molnupiravir (USAN)
D02744  5.902(2)    -    Infergen (TN), Advaferon (TN), Interferon alfacon-1 (USAN/INN), Interferon alfacon-1 (genetical recombination) (JAN)
D00024  6.782(3)  5.908  Sastid (TN), Sulfur, sublimed (USP), Bensulfoid (TN), Sulfur (JP17), Sulfur, precipitated (USP)
D00932  6.273(2)  5.908  Calcium carbonate, precipitated (JAN), Cal-sup (TN), Calcium carbonate (USP), Precipitated calcium carbonate (JP17)
D10469  5.912(3)  6.108  Sovriad (TN), Simeprevir sodium (JAN), Olysio (TN)
D00362  5.914(3)  8.102  Lisinopril (USP), Lisinopril hydrate (JP17), Lisinopril dihydrate, Prinivil (TN), Zestril (TN)
D08131  5.914(3)  8.102  Lisinopril (INN), Zestril (TN)
D10268  5.914(3)  8.102  Hydrochlorothiazide and lisinopril, Zestoretic (TN), Prinzide (TN)
D01144  6.911(2)  5.916  Prulifloxacin (JAN/INN), Sword (TN)
D02697  6.595(2)  5.919  Valrubicin (USP/INN), Valstar (TN)
D10605  5.925(3)    -    Tenofovir alafenamide fumarate (USAN/JAN), Vemlidy (TN)
D10655* 5.930(3)    -    Plazomicin sulfate (USAN), Zemdri (TN)
D10366  6.075(3)  5.931  Sofosbuvir (JAN/USAN), Sovaldi (TN)
D10827  6.075(3)  5.931  Epclusa (TN), Sofosbuvir and velpatasvir
D10900  6.075(3)  5.931  Sofosbuvir, velpatasvir and voxilaprevir, Vosevi (TN)
D02248  5.932(3)    -    Levomepromazine maleate (JP17/USAN), Hirnamin (TN)
D00570  6.828(2)  5.933  Colchicine (TN), Colchicine (JP17/USP)
D11574  6.828(2)  5.933  Col-probenecid (TN), Probenecid and colchicine
D00902  5.936(2)  7.265  Relenza (TN), Zanamivir (USP/INN)
D01937  5.936(2)  7.265  Zanamivir hydrate (JAN), Relenza (TN)
D03203  5.938(3)  7.866  Abatacept (USAN/INN), Abatacept (genetical recombination) (JAN), Orencia (TN)
D01118  5.954(4)    -    Ethopropazine hydrochloride, Parsidol (TN), Parkin (TN), Profenamine hydrochloride (JAN)
D03276  5.954(4)    -    Profenamine hibenzate (JAN), Parkin (TN)
D00672  5.956(2)  7.317  Atarax (TN), Hydroxyzine hydrochloride (JP17/USP), Vistaril (TN), Hydroxyzine dihydrochloride
D08054  5.956(2)  7.317  Marex (TN), Hydroxyzine (INN)
D00001  5.958(2)  6.008  Water for injection (JP17), Purified water in containers (JP17), Water (JP17/USP), Sterile water (TN), Purified water (JP17), Water for injection in containers (JP17), Sterile purified water in containers (JP17), Water, purified (USP)
D06249  5.958(2)  6.008  Tritiotope (TN), Tritiated water, Water, tritiated (USAN)
D02293  5.958(3)  7.820  Calcium ascorbate (USP)
D05853  5.958(3)  7.820  Sodium ascorbate (USP/INN), Cevalin (TN)
D07575  5.958(3)  7.820  Calcium Ascorbate (TN), Ascorbic acid calcium salt
D00435  5.969(2)  8.973  Nevirapine (JAN/USP/INN), Viramune (TN)
D09296  5.969(2)    -    Sophorae subprostratae radix, Sophora subprostrata root (Non-JPS)
D07057  5.971(2)  7.954  Abacavir (INN)
D10893  6.550(2)  5.975  Calquence (TN), Acalabrutinib (JAN/USAN/INN)
D00140  5.979(2)  6.325  Akne-mycin (TN), Erythromycin (JP17/USP/INN), T-stat (TN), Erygel (TN), Pce (TN), Staticin (TN), Eryc (TN)
D11646  5.979(2)  8.163  Benzoyl peroxide and erythromycin, Aktipak (TN)
D07776  6.488(2)  5.984  DM, Daunorubicin (INN), DaunoXome (TN)
D00421  6.000(3)  7.636  Ramipril (USP/INN), Altace (TN)
D05200  7.040(2)  6.001  Merital (TN), Nomifensine maleate (USAN)
D08772  7.770(4)  6.007  Sulperazon (TN), Cefoperazone sodium and sulbactam sodium (JP17)
D11216  6.319(2)  6.007  Presatovir (USAN/INN)
D02126  6.008(2)  8.227  Primaquine (TN), Primaquine phosphate (JAN/USP)
D08420  6.008(2)  8.227  Kanaprim (TN), Primaquine (INN)
D01167  6.010(2)  6.219  Magnesium oxide (JP17/USP), Magmitt (TN)
D01679  6.010(2)  6.219  Calcium oxide (JP17), Lime (USP)
D03436  6.010(2)  6.219  Cellulose, oxidized (JAN/USP), Surgicel (TN)
D08696  6.010(2)  6.219  Asgen (TN), Acetaminophen, ephedra herb, scopolia extract, caffeine and sodium benzoate and magnesium oxide
D08711  6.010(2)  6.219  Coptis rhizome, senna leaf, rhubarb, magnesium oxide and magnesium sulfate hydrate, Cetilo (TN)
D10793  6.010(2)  6.219  Prepopik (TN), Picoprep (TN), Sodium picosulfate hydrate, magnesium oxide and citric acid, anhydrous, Clenpiq (TN)
D06457  6.016(4)  8.423  HCG (TN), A.P.L. (TN), Gonadotropin, chorionic (USP), Human chorionic gonadotrophin (JP17)
D02573  6.029(3)    -    Reboxetine mesylate (USAN), Reboxetine mesilate, Vestra (TN)
D04472  6.040(3)  6.542  Metharmon-F (TN), Pregnenolone, androstenedione, testosterone, estrone and dried thyroide
D06482  6.040(3)  6.542  Thyroid (USP), Thyradin (TN)
D00899  6.042(3)  7.031  Viracept (TN), Nelfinavir mesylate (USAN/INN), Nelfinavir mesilate (JAN)
D11278  6.046(2)  6.199  Baloxavir (USAN)
D06177  7.174(2)  6.046  Orinase diagnostic (TN), Tolbutamide sodium, sterile
D11054  7.027(2)  6.047  Ravulizumab (genetical recombination) (JAN), Ultomiris (TN), Ravulizumab (USAN)
D04620  6.050(3)    -    Iseganan hydrochloride (USAN)
D07725  6.050(3)    -    Heminevrin (TN), Clomethiazole edisilate
D06886  6.052(2)  6.357  Tysabri (TN), Natalizumab (genetical recombination) (JAN), Natalizumab (USAN/INN)
D09884  6.828(2)  6.053  Danoprevir (USAN/INN)
D09885  6.828(2)  6.053  Danoprevir sodium (USAN)
D00072  6.055(4) 10.877  Cytorest (TN), Cytochrome c (JAN)
D10335* 6.060(3)    -    Alirocumab (USAN), Praluent (TN)
D09244  8.573(2)  6.060  Myristicae semen, Powdered nutmeg (Non-JPS), Nutmeg (JP17)
D09719  6.774(2)  6.060  Prochymal (TN), Remestemcel-L (USAN)
D10493  6.991(2)  6.060  Trabodenoson (USAN/INN)
D11154  6.774(2)  6.060  Diroximel fumarate (USAN/INN), Vumerity (TN)
D03484  6.060(2)  6.337  Catarase (TN), Chymotrypsin (JAN/USP/INN)
D00836  7.950(3)  6.063  Buprenorphine hydrochloride (JP17/USP), Buprenex (TN)
D07132  7.950(3)  6.063  Buprenorphine (JAN/INN), Temgesic (TN)
D10250  7.708(3)  6.063  Buprenorphine and naloxone, Bunavail (TN), Suboxone (TN)
D04187  7.032(2)  6.068  Gilenya (TN), Fingolimod hydrochloride (JAN/USAN)
D10001  7.032(2)  6.068  Gilenya (TN), Fingolimod (INN)
D10081  6.083(3)  6.169  Simeprevir (JAN/USAN)
D02261  6.090(2)  7.589  Quinine hydrochloride dihydrate, Quinine hydrochloride (TN), Quinine hydrochloride hydrate (JP17)
D08460  6.090(2)  7.589  Quinine (BAN), Kinder Quinina (TN)
D08461  6.090(2)  7.589  Quinine (TN), Quinine dihydrochloride
D08810  6.090(2)  7.319  Quinine hydrochloride, sodium chloride, tartaric acid and sucrose
D08834  6.740(2)  6.090  Stomachic and digestive, Ohara (TN)
D02367  6.091(3)    -    Desogestrel (USAN/INN)
D00027  6.095(2)  8.208  Uracil (JAN/USAN)
D02131  6.095(2)  8.208  Uftoral (TN), Tegafur and uracil
D09776  6.095(2)  8.208  Uracil (2-13C) (JAN)
D00627  6.098(2)  7.593  Micardis (TN), Telmisartan (JP17/USP/INN)
D09219  6.098(2)  7.593  Telmisartan and hydrochlorothiazide (JP17), Micombi (TN), Micardis hct (TN)
D09743  6.098(2)  7.593  Twynsta (TN), Telmisartan and amlodipine besilate, Telmisartan and amlodipine besylate, Micamlo (TN)
D10805  6.098(2)  7.593  Telmisartan, amlodipine and hydrochlorothiazide, Micatrio (TN)
D04112  6.102(2)  8.722  Etravirine (JAN/USAN/INN), Intelence (TN)
D05916  6.172(2)  6.102  Herperal (TN), Stallimycin hydrochloride (USAN)
D08751  6.555(2)  6.102  Jeoase (TN), Semi-alkaline proteinase
D09003  6.389(2)  6.102  Sonedenoson (USAN)
D10185  6.389(2)  6.102  Vortioxetine hydrobromide (JAN/USAN), Brintellix (TN), Trintellix (TN)
D11633  6.172(2)  6.102  Venglustat (USAN/INN)
D02626  6.822(3)  6.108  Bromperidol decanoate (USAN)
D09354  6.345(2)  6.108  Metafolin (TN), Levomefolinate calcium, Levomefolate calcium (USAN)
D10772  6.822(2)  6.108  Lasvic (TN), Lascufloxacin hydrochloride (JAN)
D00157  6.443(2)  6.113  Glycyrrhizin (JAN), Glycyrrhizic acid
D00473  6.113(2)  6.513  Prednisone (USP), Deltasone (TN), Rayos (TN), Prednisone monohydrate, Meticorten (TN)
D00863  6.334(2)  6.116  Povidone-iodine (JP17/USP), Betadine (TN)
D05519  6.117(3)  6.328  Pituitrin (TN), Pituitary, posterior
D00523  6.118(2)  8.550  Irbesartan (JP17/USP/INN), Avapro (TN)
D10243  6.118(2)  8.550  Aimix (TN), Irbesartan and amlodipine besilate (JP17)
D10249  6.118(2)  8.550  Irbesartan and hydrochlorothiazide, Ifirmacombi (TN), Avalide (TN)
D10480  6.118(2)  8.550  Irbesartan and trichlormethiazide, Irtra (TN)
D00408  7.954(2)  6.122  Methyltestosterone (JP17/USP/INN), Testred (TN), Android (TN)
D03975  6.849(2)  6.128  Apronalide, Apronal, Allylisopropylacetylurea
D10428  6.130(3)  6.670  Tenofovir alafenamide (USAN/INN)
D10087  6.135(2)  6.228  Alisporivir (USAN)
D01612  6.135(4)    -    Laxoberal (TN), Sodium picosulfate hydrate (JP17), Laxoberon (TN)
D10172  7.128(2)  6.136  Teriflunomide (USAN), Aubagio (TN)
D00318  7.285(2)  6.139  Pepcid (TN), Fluxid (TN), Famotidine (JP17/USP/INN)
D10259  6.273(2)  6.139  Famotidine, calcium carbonate and magnesium hydroxide, Pepcid complete (TN)
D11575  7.285(2)  6.139  Duexis (TN), Ibuprofen and famotidine
D01710  6.139(2)    -    Rulide (TN), Roxithromycin (JP17/USAN/INN)
D03753  6.141(3)    -    Perindopril (USAN/INN)
D10845  7.122(2)  6.142  Ruconest (TN), Conestat alfa (INN)
D11932  6.143(4)    -    Halobetasol and tazarotene, Ulobetsol and tazarotene, Duobrii (TN)
D07086  6.148(2)  6.839  Rivaroxaban (JAN/USAN/INN), Xarelto (TN)
D00414  7.082(2)  6.151  Zileuton (USP/INN), Zyflo (TN)
D09233  7.082(2)  6.151  Prunus mume fruit, Mume fructus, Processed mume (Non-JPS)
D00018  6.155(4)  7.728  Ascorbic acid (JP17/USP/INN), Ascoltin (TN), Ascorbicap (TN), ASCOR (TN)
D03969  6.155(4)  6.737  E.A.C (TN), Aspirin and ascorbic acid
D04899  6.155(4)  7.075  Thiamine chloride hydrochloride, riboflavin sodium phosphate and ascorbic acid
D04909  6.155(4)  7.728  Cinal (TN), Ascorbic acid and calcium pantothenate (JP17)
D04967  6.155(4)  7.236  Carbazochrome, phytonadione and ascorbic acid, Ophthalm K (TN)
D08739  6.155(4)  7.728  L-Cysteine and ascorbic acid, Crystfan (TN)
D07413  6.156(3)    -    Pantothenic acid (BAN), Pantothen Pharmaselect (TN)
D00313  6.157(2)  8.531  Ethacrynic acid (USP), Etacrynic acid (JP17/INN)
D08124  6.159(2)    -    Levalbuterol, Levosalbutamol (INN)
D00034  6.159(2)  8.535  Adenine (JAN/USP), Leucon (TN)
D10574  6.161(3)  8.537  Pembrolizumab (USAN), Pembrolizumab (genetical recombination) (JAN), Keytruda (TN)
D00413  6.167(2)  8.250  Retrovir (TN), Zidovudine (JP17/USP/INN)
D00445  6.167(2)  8.707  Stavudine (USAN/INN), Sanilvudine (JAN), Zerit (TN)
D04079  6.170(3)  8.544  Ethacrynate sodium (USP)
D00041  6.170(2)  8.755  L-Threonine (JP17), Threonine (USP)
D09707  6.174(2)  8.106  Dabigatran (USAN/INN)
D10065  6.176(3)  6.441  Daclatasvir (USAN)
D10882  6.176(3)  6.346  Daclatasvir, asunaprevir and beclabuvir, Ximency (TN)
D02481  6.179(2)  6.940  Artemisinin (INN), Qing Hau Sau
D00746  6.801(2)  6.181  Betaseron (TN), Interferon beta-1b (genetical recombination) (JAN), Extavia (TN), Interferon beta-1b (USAN/INN)
D00023  6.382(3)  6.185  Pastaron (TN), Urea (JP17/USP), Carbamide
D01749  7.139(2)  6.185  Helicosol (TN), Urea C13 (USP), Urea (13C) (JAN)
D10590  6.187(3)  9.623  Mononessa (TN), Norgestimate and ethinyl estradiol, Trinessa (TN)
D10839  6.187(3)  9.623  Norelgestromin and ethinyl estradiol, Evra (TN), Xulane (TN)
D10557* 6.188(2)    -    Repatha (TN), Evolocumab (USAN/INN), Evolocumab (genetical recombination) (JAN)
D03068  6.190(2)  8.711  Belimumab (genetical recombination) (JAN), Benlysta (TN), LymphoStat-B (TN), Belimumab (USAN)
D09139  9.097(4)  6.191  Myrobalan, Myrobalan fruit (Non-JPS), Chebulae fructus
D10078  7.122(2)  6.191  Peginterferon lambda-1a (USAN)
D05095  6.310(2)  6.201  Myfortic (TN), Mycophenolate sodium (USP)
D08092  6.201(3)  6.426  Dilator (TN), Isoxsuprine (INN)
D09967  6.203(3)  6.983  Cosentyx (TN), Secukinumab (USAN/INN), Secukinumab (genetical recombination) (JAN)
D04365  6.905(2)  6.205  Glycyrrhiza (JP17), Liquorice, Prepared glycyrrhiza (JP17), Glycyrrhizae radix, Glycyrrhiza (TN), Licorice (NF), Glycyrrhizae radix praeparata, Powdered glycyrrhiza (JP17)
D09315  7.177(3)  6.205  Ilaris (TN), Canakinumab (USAN/INN), Canakinumab (genetical recombination) (JAN)
D08710  6.905(2)  6.207  Sulfur, fennel, glycyrrhiza and senna leaf, Compound glycyrrhiza (TN)
D00025  6.207(3)  6.922  Sugar spheres (NF), Sucrose, purified, Sugar, compressible (NF), Sucrose (TN), Sugar, confectioner's (NF), Sucrose (JP17/NF), White soft sugar (JP17)
D10288  6.209(3)  7.708  Tribenzor (TN), Olmesartan medoxomil, amlodipine besylate and hydrochlorothiazide
D00022  6.213(4)  7.515  L-Tyrosine (JP17), Tyrosine (USP/INN)
D10794  6.977(2)  6.214  Onpattro (TN), Patisiran sodium (JAN)
D00848  6.215(3)    -    Benylin DM (TN), Dextromethorphan hydrobromide (USP), Dextromethorphan hydrobromide hydrate (JP17)
D03257  6.217(2)  9.012  Trastuzumab-pkrb, Trastuzumab-qyyp, Trastuzumab-anns, Trastuzumab-dkst, Trastuzumab-dttb, Trastuzumab (genetical recombination) (JAN), Herceptin (TN), Herzuma (TN), Ogivri (TN), Trastuzumab (genetical recombination) [Trastuzumab biosimilar 3] (JAN), Kanjinti (TN), Trastuzumab (genetical recombination) [Trastuzumab biosimilar 1] (JAN), Trastuzumab (USAN/INN), Trastuzumab (genetical recombination) [Trastuzumab biosimilar 2] (JAN)
D11560  6.217(2)  9.012  Trastuzumab and hyaluronidase-oysk, Herceptin hylecta (TN), Trastuzumab and hyaluronidase
D11934  6.217(2)  8.962  Phesgo (TN), Pertuzumab, trastuzumab and hyaluronidase, Pertuzumab, trastuzumab and hyaluronidase-zzxf
D09687  6.608(2)  6.220  Ella (TN), Ulipristal acetate (JAN/USAN)
D03236  6.349(2)  6.225  Synthetic aluminum silicate (JP17), Aluminum silicate, synthetic (JAN), Silicamin (TN)
D05358  6.349(2)  6.225  Paraffin, synthetic (NF)
D00944  6.687(3)  6.228  Metformin hydrochloride (JP17/USP), Glucophage (TN)
D04966  6.687(3)  6.228  Metformin (USAN/INN)
D09744  6.687(3)  6.228  Metact (TN), Pioglitazone and metformin, Pioglitazone hydrochloride and metformin hydrochloride (JP17)
D10244  6.687(3)  6.228  Avandamet (TN), Metformin hydrochloride and rosiglitazone maleate
D10253  6.687(3)  6.228  Alogliptin benzoate and metformin hydrochloride, Vipdomet (TN), Kazano (TN)
D10261  6.687(3)  6.228  Metformin hydrochloride and sitagliptin phosphate, Janumet (TN)
D10263  6.687(3)  6.228  Metformin hydrochloride and saxagliptin hydrochloride, Kombiglyze (TN)
D10264  6.687(3)  6.228  Jentaduet (TN), Linagliptin and metformin hydrochloride
D10265  6.687(3)  6.228  Glipizide and metformin hydrochloride, Metaglip (TN)
D10266  6.687(3)  6.228  Glyburide and metformin hydrochloride, Glibenclamide and metformin hydrochloride, Glucovance (TN)
D10500  6.687(3)  6.228  Metformin hydrochloride and repaglinide, Prandimet (TN)
D10586  6.687(3)  6.228  Dapagliflozin and metformin, Xigduo xr (TN)
D10587  6.687(3)  6.228  Invokamet (TN), Canagliflozin and metformin
D10743  6.687(3)  6.228  Vildagliptin and metformin hydrochloride
D10752  6.687(3)  6.228  Empagliflozin and metformin, Synjardy (TN)
D11067  6.687(3)  6.228  Segluromet (TN), Ertugliflozin and metformin
D11109  6.687(3)  6.228  Anagliptin and Metformin hydrochloride
D11711  6.687(3)  6.228  Dapagliflozin, metformin and saxagliptin, Qternmet xr (TN)
D11856  6.687(3)  6.228  Trijardy xr (TN), Empagliflozin, linagliptin and metformin hydrochloride
D03769  6.232(2)  9.014  Enalaprilat (USP), Vasotec (TN)
D11021  6.233(3)  6.466  Baloxavir marboxil (JAN/USAN/INN), Xofluza (TN)
D00007  6.234(2)  9.454  Glutamic acid (USP), L-Glutamic acid (JP17)
D04341  6.234(2) 10.532  DL-Glutamic acid, Glutamic acid (USAN)
D07539  6.234(2) 10.532  Hypochylin (TN), Glutamic acid hydrochloride
D07729  6.236(3)  7.651  Plavix (TN), Clopidogrel (TN), Clopidogrel (USP/INN)
D10513  6.236(3)  6.737  Complavin (TN), Clopidogrel sulfate and aspirin, Clopidogrel and acetylsalicyclic acid
D10823  6.236(3)  7.651  Clopidogrel (TN), Clopidogrel hydrochloride
D10824  6.236(3)  7.651  Clopidogrel besylate, Clopidogrel (TN), Clopidogrel besilate
D00146  6.238(3)  9.082  Acthar (TN), Corticotropin (USP/INN), ACTH (TN)
D07008  6.238(2)    -    Chikujountanto
D04812  6.456(2)  6.243  Sucrose and povidone iodine, Sorenurse (TN)
D05121  6.533(2)  6.246  Naproxol (USAN/INN)
D09894  6.316(2)  6.246  Eliglustat hemitartrate, Eliglustat tartrate (JAN/USAN), Cerdelga (TN)
D10331  6.533(2)  6.246  Ozanezumab (USAN)
D10414  6.316(2)  6.246  Brilacidin (USAN)
D02028  6.483(2)  6.246  Diammonium glycyrrhizinate (JAN)
D05246  6.249(2)  8.476  Olmesartan (USAN/INN)
D08812  6.254(4)  6.888  Factor XIII with fibrinogen, Beriplast P combi-set (TN), Aprotinin, thrombin, human blood-coagulation factor XIII fraction, calcium chloride hydrate and freeze-dried human fibrinogen
D09731  6.257(2)  9.328  Crizotinib (JAN/USAN/INN), Xalkori (TN)
D02472  6.258(2)  7.476  Malarone (TN), Atovaquone and proguanil hydrochloride
D02487  6.258(2)  8.905  Paludrine (TN), Proguanil hydrochloride (JAN/USP), Chloroguanide hydrochloride
D08428  6.258(2)  8.905  Proguanil (INN)
D08778  6.265(4)  8.898  Aimmugen (TN), Freeze-dried inactivated tissue cluture hepatitis A vaccine, Freeze-dried, inactivated hepatitis A vaccine (JAN)
D00975  6.267(3)  7.681  Dexamethasone sodium phosphate (JAN/USP), Dalalone (TN), Maxidex (TN)
D02485  6.272(2)  7.353  Halofantrine hydrochloride (USAN), Halfan (TN)
D08033  6.272(2)  7.353  Halofantrine (INN)
D01446  6.273(2)  6.696  Magnesium carbonate (JP17/USP), Magnesium carbonate (TN)
D01780  6.273(2)  6.696  Sodium carbonate hydrate (JP17)
D02038  6.273(2)  6.696  Racol (TN), Potassium carbonate (JP17/USP)
D03960  6.273(2)  6.696  Aspirin and dialuminate, Aspirin, aluminum glycinate and magnesium carbonate, Bufferin (TN)
D07631  6.273(2)  6.696  Ethyl piperidinoacetylaminobenzoate, magnesia alumina hydrate and precipitated calcium carbonate, Sulcain (TN)
D07703  6.273(2)  6.696  Hydroxyethyl cellulose, boric acid, dibasic sodium phosphate, potassium chloride, sodium chloride and dried sodium carbonate, Scopisol (TN)
D08704  6.273(2)  6.696  Cabagin-U (TN), Magnesium aluminometasilicate, methylmethionine sulfonium chloride, magnesium carbonate and precipitated calcium carbonate
D10802  6.273(2)  6.696  Calcium carbonate (13C) (JAN), Carbonic-13C acid, calcium salt
D01583  7.198(2)  6.276  Pirfenidone (JAN/USAN/INN), Esbriet (TN)
D10630  7.256(2)  6.276  Fedratinib (USAN/INN)
D11296  7.256(2)  6.276  Fedratinib hydrochloride (USAN), Inrebic (TN), Fedratinib dihydrochloride monohydrate
D02490  6.278(3)  6.624  TS, Tylan (TN), Tylosin (USP/INN)
D08783  6.282(3)    -    Polyethylene glycol treated human normal immunoglobulin, Venoglobulin-IH (TN)
D08786  6.282(3)    -    Freeze-dried sulfonated human normal immunoglobulin, Venilon-I (TN)
D02295  6.630(3)  6.283  Vinisil (TN), Polyvinylpyrrolidone, Povidone (JP17/USP/INN)
D03383  6.283(2)    -    Murine ear drops (TN), Carbamide peroxide (USP)
D03213  6.291(2)  6.931  Eliquis (TN), Apixaban (JAN/USAN/INN)
D08402  6.294(2)  8.457  Curosurf (TN), Poractant alfa (BAN)
D04409  6.294(5)    -    Halobetasol propionate (USP), Ultravate (TN), Ulobetasol propionate
D06019  7.226(2)  6.295  Tecadenoson (USAN/INN)
D02994  6.766(3)  6.298  Rituximab (USAN/INN), Rituximab (genetical recombination) [Rituximab biosimilar 2] (JAN), Rituximab (genetical recombination) [Rituximab biosimilar 1] (JAN), Truximab (TN), Rituximab (genetical recombination) (JAN), Rituximab-pvvr, Rituxan (TN), Rituximab-abbs
D11393  6.766(3)  6.298  Rituxan hycela (TN), Rituximab and hyaluronidase
D00924  6.303(2)  6.903  Ceftriaxone sodium hydrate (JP17), Ceftriaxone sodium (USP), Rocephin (TN)
D07659  6.303(2)  6.903  Ceftriaxone (TN), Ceftriaxone (INN)
D03597  6.304(2)  6.617  Cotton, purified (USP)
D05707  6.304(2)  6.922  Rayon, Rayon, purified (USP)
D05839  6.304(2)  6.922  Siliceous earth, purified (NF)
D06459  6.304(2)  6.922  Human menopausal gonadotrophin, purified, Purified human menopausal gonadotrophin (JAN)
D06776  6.304(2)  6.922  Honey (TN), Honey (JP17), Honey, purified (NF)
D10278  6.304(3)  9.861  Quinapril hydrochloride and hydrochlorothiazide, Accuretic (TN)
D00459  6.304(3)    -    Accupril (TN), Quinapril hydrochloride (JP17/USP)
D03752  6.304(3)    -    Quinapril (INN)
D01172  6.307(3)  8.096  Diphenylpyraline hydrochloride (JAN), Vena (TN), Hispril (TN)
D03360  6.307(3)  8.096  Vena (TN), Diphenhydramine laurylsulfate (JAN)
D10639  6.315(3)  6.310  Grazyna (TN), Grazoprevir (USAN), Grazoprevir hydrate (JAN)
D10778  6.315(3)  6.310  Elbasvir and grazoprevir, Zepatier (TN)
D11565  6.315(3)  6.310  Grazoprevir (USAN/INN), Grazoprevir anhydrous
D00075  6.316(3)  7.321  Androderm (TN), Testosterone (JAN/USP), Striant (TN), Androgel (TN), Testim (TN), Axiron (TN)
D01921  6.316(3)  7.321  Estradiol and testosterone, Bothermon (TN)
D07423  6.317(3)  7.881  Defitelio (TN), Defibrotide sodium (JAN/USAN)
D06579  6.317(3)    -    Denibulin hydrochloride (USAN)
D04316  6.327(3)    -    Gestodene (USAN/INN)
D10312  6.618(2)  6.332  Clazakizumab (USAN/INN)
D05987  6.332(4)    -    Enjuvia (TN), Synthetic conjugated estrogens, B (USAN)
D03985  6.463(2)  6.335  Emetine hydrochloride (USP)
D10909  6.338(3)  7.201  Bictegravir (USAN/INN)
D10910  6.338(3)  7.201  Bictegravir sodium (JAN/USAN)
D00752  6.344(2)  6.635  Cellcept (TN), Mycophenolate mofetil (JAN/USP)
D05094  6.344(2)  6.635  Mycophenolate mofetil hydrochloride (USAN), Cellcept (TN)
D10610  6.633(2)  6.346  Beclabuvir (USAN/INN)
D10611  6.633(2)  6.346  Beclabuvir hydrochloride (JAN/USAN)
D00436  6.488(2)  6.350  Niclocide (TN), Niclosamide (USAN/INN)
D07822  8.933(3)  6.354  Didecyldimethylammonium chloride, Alfa Bergamon (TN)
D09616  7.068(2)  6.354  Filibuvir (USAN/INN)
D09960  7.359(2)  6.354  Jakavi (TN), Ruxolitinib phosphate (JAN/USAN), Jakafi (TN)
D09636  6.644(2)  6.357  Litronesib (USAN/INN)
D11253  9.621(3)  6.357  Bermekimab (USAN)
D11478  8.674(5)  6.357  Branebrutinib (USAN)
D03940  6.548(3)  6.362  Soliris (TN), Eculizumab (USAN/INN), Eculizumab (genetical recombination) (JAN)
D03917  6.366(3)    -    Drospirenone (JAN/USP/INN), Slynd (TN)
D04431  6.366(3)    -    Bayhep B (TN), Fovepta (TN), Hepatitis B immnoglobulin, Hepatitis B immune globulin (USP)
D00874  6.836(2)  6.368  Avelox (TN), Moxifloxacin hydrochloride (JAN/USP), Vigamox (TN)
D02141  7.446(2)  6.376  Infed (TN), Iron dextran (USP)
D02837  7.624(3)  6.378  Alteplase (USP/INN), Cathflo activase (TN), Activase (TN), Alteplase (genetical recombination) (JAN)
D03433  6.669(2)  6.382  Celgosivir hydrochloride (USAN)
D06798  8.559(3)  6.382  Coix extract, Coix seed (JP17), Coicis semen (TN), Powdered coix seed (JP17)
D00126  7.549(2)  6.383  Ibuprofen (JP17/USP/INN), Advil (TN), Motrin (TN)
D02152  7.549(2)  6.383  Hydrocodone bitartrate and ibuprofen, Vicoprofen (TN)
D08059  7.549(2)  6.383  Esprenit (TN), Ibuprofen sodium anhydrous, Ibuprofen sodium
D09760  7.549(2)  6.383  Ibuprofen sodium (USAN), Ibuprofen sodium hydrate
D10449  7.549(2)  6.383  Ibuprofen and scopolamine butylbromide
D11573  7.549(2)  6.383  Oxycodone and ibuprofen
D11897  7.549(2)  6.383  Acetaminophen and ibuprofen
D07747  6.384(3)  6.569  Rowatin (TN), alpha, beta-Pinene, borneol, anetholtrithion, d-camphene, cineole and fenchone
D00361  6.386(3)  6.557  Liotrix (USP), Thyrolar (TN), Levothyroxine sodium and liothyronine sodium
D01010  6.386(3)  7.846  Levothyroxine sodium hydrate (JP17), Levoxyl (TN), Synthroid (TN), Levothroid (TN), Levothyroxine sodium (USP)
D08125  6.386(3)  7.846  Forthyron (TN), Levothyroxine (BAN)
D11113  6.386(3)  7.846  Levothyroxine sodium (INN), Levothyroxine sodium anhydrous
D00109  6.387(3)  6.737  Acetylsalicylic acid, Easprin (TN), Durlaza (TN), Aspalon (JAN), Aspirin (JP17/USP)
D02079  6.387(3)  6.737  Empirin compound (TN), Codein phosphate and aspirin
D02154  6.387(3)  6.737  Codoxy (TN), Percodan-demi (TN), Aspirin, oxycodone hydrochloride and oxycodone terephthalate
D02155  6.387(3)  6.737  Aspirin, caffeine and propoxyphene hydrochloride, Darvon compound-65 (TN)
D07582  6.387(3)  6.737  Catalgine (TN), Aspirin sodium, Sodium acetylsalicylate
D11176  6.387(3)  6.737  Percodan (TN), Oxycodone hydrochloride and aspirin
D11586  6.387(3)  6.737  Carisoprodol and aspirin
D11587  6.387(3)  6.737  Carisoprodol, aspirin and codeine phosphate
D11615  6.387(3)  6.737  Aggrenox (TN), Aspirin and dipyridamole
D11616  6.387(3)  6.737  Yosprala (TN), Aspirin and omeprazole
D11804  6.387(3)  6.737  Aspirin and vonoprazan, Cabpirin (TN)
D11849  6.387(3)  6.737  Orphenadrine citrate, aspirin and caffeine, Norgesic forte (TN)
D11859  6.387(3)  6.737  Butalbital, aspirin and caffeine, Lanorinal (TN), Fiorinal (TN)
D11860  6.387(3)  6.737  Butalbital, aspirin, caffeine and codeine, Fiorinal with codeine (TN)
D00082  6.391(2)    -    Pyridoxamine phosphate (JAN), Pyridoxamine phosphate dihydrate
D00296  6.392(2)  9.273  Videx (TN), Didanosine (JAN/USP/INN)
D01370  7.039(4)  6.393  Leustatin (TN), Cladribine (JAN/USP/INN), Mavenclad (TN)
D04554  6.588(2)  6.394  Interferon beta-1a (USAN), Interferon beta-1a (genetical recombination) (JAN), Rebif (TN), Avonex (TN)
D11131  6.397(2)  6.543  Apabetalone (USAN/INN)
D02370  6.400(3)  6.858  Clavulanate potassium (JP17/USP), CVA
D10093  6.403(4)  8.493  Asunaprevir (JAN/USAN), Sunvepra (TN)
D03208  6.404(2)  9.016  Rasilez (TN), Aliskiren (USAN/INN)
D10290  6.404(2)  7.593  Tekamulo (TN), Aliskiren and amlodipine, Rasilamlo (TN)
D01108  6.405(2)  7.145  Magnesium sulfate (USP), Magnesium sulfate hydrate (JP17), Conclyte-Mg (TN), Magnesium sulfate heptahydrate
D01726  6.405(2)  7.145  Potassium sulfate (JP17/USAN)
D05877  6.405(2)  7.145  Sodium sulfate hydrate (JP17), Natrium Sulfuricum, Sodium sulfate (USP), Natrii Sulfus, Sodium sulfate decahydrate, Sodium sulfate (TN)
D05963  6.405(2)  7.145  Sulfate, Sulfuric acid (NF)
D09201  6.405(2)  7.145  Calcium sulfate (NF)
D11552  6.405(2)  7.145  Moviprep (TN), Polyethylene Glycol 3350, sodium sulfate, sodium chloride, potassium chloride, sodium ascorbate and ascorbic acid, Plenvu (TN)
D11985  6.405(2)  7.145  Sulprep (TN), Magnesium sulfate, anhydous sodium sulfate and potassium sulfate
D00258  6.407(2)  8.578  Cefixime (INN)
D07640  6.407(2)  8.578  Cefixime (USP), Suprax (TN), Cefixime hydrate (JP17), Oroken (TN), Cefixime trihydrate
D00751  6.408(3)  6.894  Solu-medrol (TN), A-methapred (TN), Methylprednisolone sodium succinate (JAN/USP)
D10625  6.412(3)  6.759  Erelsa (TN), Elbasvir (JAN/USAN/INN)
D10527  6.415(4)  7.716  Takelda (TN), Acetylsalicylic acid and lansoprazole
D10751  6.416(3)  7.530  Contrave (TN), Naltrexone and bupropion
D00496  6.419(2)  9.843  Penicillamine (JAN/USP/INN), Cuprimine (TN), Depen (TN)
D08330  6.419(2)  9.843  Pemine (TN), Penicillamine hydrochloride
D00101  6.425(2)  9.314  Vasopressin (JP17/USP), Pitressin (TN), Vasostrict (TN)
D00056  6.434(2)  7.056  Trypsin (JAN), Parenzyme (TN), Trypsin, crystallized (USP)
D04756  6.434(2)  7.056  Francetin T (TN), Fradiomycin sulfate and trypsin, crystallized
D08753  6.434(2)  7.056  Kimotab (TN), Bromelains and trypsin, crystallized
D05857  6.435(3)    -    Ceresine (TN), Sodium dichloroacetate (USAN)
D03096  6.438(2)    -    Beractant (USAN), Survanta (TN)
D09996  6.440(4)  9.239  Vemurafenib (JAN/USAN/INN), Zelboraf (TN)
D06947  6.441(2)    -    Keishito
D01078  6.444(2)  8.891  Telithromycin (JAN/USAN/INN), Ketek (TN)
D01211  6.444(5)  9.878  Uzel (TN), Wellcovorin (TN), Calcium folinate (JP17), Leucovorin calcium (USP)
D07986  6.444(5)  9.878  Folinic acid (BAN), Leucovorin, Rescuvolin (TN)
D07987  6.444(5)  9.878  Folinic acid calcium salt pentahydrate, Leucovorin calcium pentahydrate, Leucovorin (TN)
D04682  6.444(3)    -    Lecithin (NF)
D03053  6.444(2)  7.337  Bandage, adhesive (USP), Adhesive bandage
D06006  6.444(2)  6.998  Tape, adhesive (USP)
D07782  6.445(2)    -    Delavirdine (INN)
D08660  6.446(6)    -    Halobetasol, Ulobetasol (INN)
D11531  6.735(2)  6.449  Narsoplimab (USAN)
D11654  7.136(3)  6.449  Annovera (TN), Ethinylestradiol and segesterone
D00186  6.451(2)  8.293  Ciprofloxacin (JP17/USP/INN), Cipro (TN), Otiprio (TN)
D02216  6.451(2)  8.293  Ciloxan (TN), Proquin XR (TN), Cipro (TN), Ciprofloxacin hydrochloride hydrate (JP17), Ciprofloxacin hydrochloride (USP)
D10822  6.451(2)  8.293  Ciprofloxacin hemiheptahydrate, Ciprofloxacin hydrate (JAN)
D11089  6.451(2)  8.293  Otovel (TN), Ciprofloxacin hydrochloride and fluocinolone acetonide
D11582  6.451(2)  6.853  Ciprofloxacin hydrochloride and hydrocortisone, Cipro hc (TN)
D02970  6.453(4)    -    Aprinocarsen sodium (USAN), Affinitak (TN)
D08729  7.012(2)  6.453  Salicylic acid and zinc oxide, Zinc and salicylic acid (TN)
D00979  6.454(3)    -    Depo-medrol (TN), Methylprednisolone acetate (JAN/USP)
D00116  6.457(3)  8.376  Glucagon (genetical recombination) (JAN), Glucagon (JAN/USP/INN), Glucagon (TN)
D02118  6.457(3)  8.376  Glucagon monohydrochloride, Glucagon (TN), Glucagen (TN), Glucagon hydrochloride
D03139  7.495(2)  6.459  Blood group specific substances A, B, and AB
D01398  7.853(2)  6.462  Dermatol (TN), Bismuth subgallate (JP17/USP)
D09017  6.462(3)  8.716  Ticagrelor (JAN/USAN/INN), Brilinta (TN)
D11316  6.463(2)    -    Lumicitabine (USAN)
D04946  6.678(2)  6.466  Ferric pyrophosphate, soluble (JAN), Incremin (TN)
D04536  6.467(2)  8.155  FluShield (TN), Influenza virus vaccine (USP), Fluarix quadrivalent (TN)
D04747  6.467(3)    -    Elvanse (TN), Vyvanse (TN), Lisdexamfetamine dimesylate (USAN), Lisdexamfetamine mesilate (JAN)
D00013  6.467(3)  8.719  Aspartic acid (USP/INN), L-Aspartic acid (JP17)
D07913  6.468(2)  7.647  Esertia (TN), Escitalopram (INN)
D00307  6.469(2)  6.635  Doxycycline hydrate, Vibramycin (TN), Doxycycline (USP), Monodox (TN), Oracea (TN)
D02129  6.469(2)  6.635  Vibra-tabs (TN), Periostat (TN), Doxycycline hydrochloride hydrate (JP17), Lymepak (TN), Doryx (TN), Doxycycline hyclate (USP)
D03903  6.469(2)  6.635  Doxycycline calcium (USP)
D07876  6.469(2)  6.635  Doxycycline (INN), Doxycycline (TN), Doxychel (TN)
D07877  6.469(2)  6.635  Doxycycline hydrochloride, Vibramycin (TN)
D00048  6.472(4)    -    Thioctic acid amide (JAN), Lipoamide
D11120  7.285(2)  6.475  Emapalumab-lzsg, Emapalumab (USAN/INN), Emapalumab (genetical recombination) (JAN), Gamifant (TN)
D10806  6.762(2)  6.475  Velpatasvir (JAN/USAN/INN)
D11222  6.988(2)  6.478  Xpovio (TN), Selinexor (USAN/INN)
D06199  6.480(2)    -    Yondelis (TN), Trabectedin (JAN/USAN/INN)
D04034  6.481(4)    -    Wintermin (TN), Chlorpromazine phenolphthalinate (JAN)
D02163  6.481(4)    -    Flumezin (TN), Fluphenazine maleate (JAN)
D00045  6.482(2)  7.330  Adenoscan (TN), Adenosine (JAN/USP), Adenocard (TN)
D00107  7.071(3)  6.482  Tacrolimus hydrate (JP17), Tacrolimus (USP/INN), Prograf (TN), Protopic (TN)
D08556  7.071(3)  6.482  Tacrolimus (INN), Prograf (TN)
D00900  6.485(2)  7.456  Oseltamivir phosphate (JAN/USP), Ebilfumin (TN), Tamiflu (TN)
D05597  6.486(3)  9.326  Effient (TN), Prasugrel hydrochloride (JAN/USAN)
D09196  6.494(2)  9.861  Ecard LD (TN), Candesartan cilexetil and hydrochlorothiazide (JP17), Ecard HD (TN)
D00626  6.494(2)    -    Atacand (TN), Candesartan cilexetil (JP17/USP)
D07569  6.494(2)    -    Unisia (TN), Candesartan cilexetil and anmlodipine besylate (JP17)
D00592  6.495(2)  8.246  Aczone (TN), Dapsone (USP), Diaphenylsulfone (JAN)
D08681  7.078(3)  6.497  Vonicog alfa (USAN), Von willebrand factor, Vonicog alfa (genetical recombination) (JAN), Vonvendi (TN), Wilfactin (TN)
D00962  6.497(3)  9.515  Clomid (TN), Serophene (TN), Clomifene citrate (JP17), Clomiphene citrate (USP)
D01093  7.212(2)  6.498  Volley (TN), Butenafine hydrochloride (JP17/USAN), Mentax (TN)
D11480  7.428(2)  6.498  Crizanlizumab (USAN/INN), Crizanlizumab-tmca, Adakveo (TN)
D06165  6.498(3)    -    Tiqueside (USAN/INN)
D02300  6.499(4)  7.893  Adenosine triphosphate disodium hydrate (JAN), Adenosine triphosphate disodium trihydrate, ATP (TN), Adenosine 5'-triphosphate disodium
D08646  6.499(4)  7.896  ATP, Triphosadenine (DCF)
D03829  6.506(2)  7.403  Peramivir hydrate (JAN), Rapivab (TN), Peramivir (USAN/INN)
D09970  6.719(2)  6.516  Tofacitinib (USAN), Tasocitinib
D02051  6.517(3)  8.531  Potassium nitrate (JAN/USP), Sensodyne (TN)
D02313  6.517(3)  8.531  Nitrate, Nitric acid (NF)
D00083  6.768(2)  6.519  Nitrogen (TN), Nitrogen (JP17/NF)
D03229  6.521(2)  8.245  BLM, Bleomycin hydrochloride (JP17), Bleo (TN)
D07535  6.521(2)  8.245  BLM, Bleomycin (INN), Bleomycin Hexal (TN)
D00615  6.522(4)  7.708  Amlodipine besylate (USP), Amlodipine besilate (JP17), Norvasc (TN)
D10285  6.522(4)  7.708  Amlodipine besylate and olmesartan medoxomil, Azor (TN)
D10291  6.522(4)  7.708  Amturnide (TN), Aliskiren hemifumarate, amlodipine besylate and hydrochlorothiazide
D00518  6.812(2)  6.526  Keveyis (TN), Daranide (TN), Dichlorphenamide (USP), Diclofenamide (JAN/INN)
D10425  6.812(2)  6.526  Lomibuvir (USAN/INN)
D00058  6.526(4) 10.420  Gammalon (TN), gamma-Aminobutyric acid (JAN)
D06286  6.528(2)  6.615  Vegetable oil, hydrogenated (NF), Hydrogenated vegetable oil
D09004  6.528(2)  6.615  Soybean oil, hydrogenated (NF)
D10994  6.536(3)    -    Upadacitinib (USAN/INN)
D11048  6.536(3)    -    Rinvoq (TN), Upadacitinib hemihydrate, Upadacitinib hydrate (JAN)
D01665  7.252(2)  6.538  Basen (TN), Voglibose (JP17/USAN/INN)
D06905  7.468(2)  6.538  Peucedanum root (JP17), Zenko, Peucedani radix
D04070  6.538(4)  7.376  Estrogens, conjugated (JAN/USP), Conjugated estrogens, Premarin (TN)
D04071  6.538(4)  7.376  Estrogens, esterified (USP), Amnestrogen (TN), Menest (TN)
D03506  6.538(2)    -    Cinanserin hydrochloride (USAN)
D11287  6.540(2)    -    Pimodivir (USAN/INN)
D11367  6.540(2)    -    Pimodivir hydrochloride (USAN), Pimodivir hydrochloride hemihydrate
D00472  6.542(3)  6.672  Delta-cortef (TN), Prednisolone (JP17/USP/INN)
D04251  6.542(3)  6.672  Tetrahydrozoline hydrochloride and prednisolone, Cor tyzine (TN)
D04753  6.542(3)  6.672  Chloramphenicol, fradiomycin sulfate and prednisolone, Chlomy-P (TN)
D04769  6.542(3)  6.672  Aersolin D (TN), Fradiomycin sulfate and prednisolone
D11699  6.542(3)  6.672  Sulfacetamide sodium and prednisolone
D03896  7.746(2)  6.543  Dornase alfa (USAN/INN), Pulmozyme (TN), Dornase alfa (genetical recombination) (JAN)
D00167  6.958(2)  6.544  Dimercaprol (JP17/USP/INN), BAL (TN)
D02482  6.547(2)  7.674  Artesunic acid, Arsumax (TN), Artesunate (TN), Artesunate (USAN)
D02499  6.547(2)    -    Fuzeon (TN), Enfuvirtide (USAN/INN)
D10624  6.550(3)    -    Doravirine (JAN/USAN/INN), Pifeltro (TN)
D03300  6.551(4)  9.031  Calcium pantothenate, racemic (USP)
D11519  6.551(4)    -    Sodium pantothenate
D01082  6.551(4)    -    Calpan (TN), Calcium pantothenate (JP17/USP/INN)
D06076  6.555(3)    -    Teprotide (USAN/INN)
D10589  6.555(3)    -    Azilsartan medoxomil and chlorthalidone, Edarbyclor (TN)
D01011  7.953(3)  6.557  Cytomel (TN), Triostat (TN), Liothyronine sodium (JP17/USP)
D10066  6.557(4)  7.395  Dolutegravir (USAN), Tivicay (TN)
D10113  6.557(4)  7.395  Dolutegravir sodium (JAN/USAN), Tivicay (TN)
D11282  6.557(4)  7.395  Juluca (TN), Dolutegravir sodium and rilpivirine hydrochloride
D05776  6.558(2)  6.753  Rupintrivir (USAN/INN)
D01655  6.560(4)    -    Hepsera (TN), Adefovir pivoxil (JAN), Adefovir dipivoxil (USAN)
D00090  6.563(2)  6.888  Thrombin (JP17/USP/INN), Factor IIa, Thrombostat (TN)
D08813  6.563(2)  6.888  Aprotinin, thrombin and human fibrinogen, Tachocomb (TN)
D02993  6.564(4)    -    Arzoxifene hydrochloride (USAN)
D07299  6.564(4)    -    Erlosamide, Lacosamide (JAN/USAN/INN), Vimpat (TN)
D03021  6.568(3)  9.081  Vidaza (TN), Azacitidine (JAN/USAN/INN)
D00563  6.571(3)  7.114  Reflex (TN), Remeron (TN), Mirtazapine (JAN/USP/INN)
D01136  7.106(2)  6.575  Rikavarin (TN), Tranexamic acid (JP17/USP/INN), Transamin (TN), Cyklokapron (TN)
D11243  7.991(3)  6.575  Ublituximab (USAN/INN)
D03260  6.578(4)    -    Levitra (TN), Staxyn (TN), Vardenafil hydrochloride hydrate (JAN), Vardenafil monohydrochloride trihydrate
D06412  6.578(4)    -    Tekturna (TN), Aliskiren hemifumarate, Aliskiren fumarate (JAN/USAN)
D05209  6.584(3)    -    Norgestimate (USP/INN)
D07056  6.585(3)    -    Rokumigan, Rokumijiogan
D06970  6.585(3)    -    Jiinkokato
D07043  6.585(2)    -    Maobushisaishinto
D05069  6.946(2)  6.585  Monensin sodium (USP)
D07858  7.031(2)  6.590  Diosmin (INN), Daflon (TN)
D01825  6.591(4)    -    Fluocinolone acetonide (JP17/USP/INN), Synalar (TN), Retisert (TN), Fluocet (TN)
D04795  6.591(4)    -    Fradiomycin sulfate and fluocinolone acetonide, Flucort F (TN), Neo-synalar (TN), Neomycin sulfate and fluocinolone acetonide
D03286  6.662(2)  6.592  Alinamin (TN), Prosultiamine (JAN/INN)
D07107  6.879(2)  6.592  Danthron, Dantron (INN), Pilules Vinchy N.F. (TN)
D07529  6.879(2)  6.592  Binifibrate (INN), Biniwas (TN)
D11094  7.306(2)  6.592  Takhzyro (TN), Lanadelumab (genetical recombination) (JAN), Lanadelumab (USAN), Lanadelumab-flyo
D00588  6.596(2)  7.432  Levofloxacin hydrate (JP17), Levofloxacin (USP), Iquix (TN), Levofloxacin hemihydrate, Quixin (TN), Levaquin (TN)
D08120  6.596(2)  7.432  Levaquin (TN), Levofloxacin (INN), Cravit (TN)
D10885  6.599(3)  8.002  Tymlos (TN), Abaloparatide (USAN/INN)
D00229  6.600(2)  7.562  Amoxil (TN), Pasetocin (TN), Amoxicilline (INN), Dispermox (TN), Amoxicillin hydrate (JP17), Amoxicillin (USP), Amoxicillin trihydrate
D00230  6.600(2)  7.562  Amoxicillin and potassium clavulanate, Amoxicillin and clavulanate potassium
D02925  6.600(2)  7.562  Amoxicillin sodium (USAN)
D06485  6.600(2)  7.562  Amoxicillin hydrate and potassium clavulanate, Augmentin (TN)
D07452  6.600(2)  7.562  Amoxicillin (INN), AMPC, Amoxicillin (TN)
D09742  6.600(2)  7.562  Lansoprazole, amoxicillin hydrate and metronidazole, Lampion (TN)
D10520  6.600(2)  7.562  Rabeprazole, amoxicillin and metronidazole, Rabefine (TN)
D10774  6.600(2)  7.562  Vonoprazan, amoxicillin and metronidazole, Vonopion (TN)
D11858  6.600(2)  7.562  Talicia (TN), Omeprazole magnesium, amoxicillin and rifabutin
D00345  6.601(4)    -    Indapamide (JP17/USP), Natrix (TN), Lozol (TN)
D06401  6.601(4)    -    Indapamide hydrate, Tenaxil (TN)
D02484  6.603(2)  8.061  Artemether and lumefantrine, Coartem (TN)
D03821  6.603(2)  8.061  Lumefantrine (JAN/USP/INN)
D00122  7.489(3)  6.604  Calderol (TN), Calcifediol monohydrate, Rayaldee (TN), Calcifediol (USP/INN)
D00565  7.588(2)  6.608  Fenofibrate (JAN/USP/INN), Triglide (TN), Lipantil (TN), Tricor (TN), Antara (TN), Lipofen (TN)
D03365  7.667(2)  6.610  Nicotine (USP), Habitrol (TN)
D06571  6.612(2)  8.242  Antithrombin III human (USP), Kybernin (TN), Antithrombin III (INN)
D03256  6.612(4)  9.029  Valcyte (TN), Valganciclovir hydrochloride (JAN/USP)
D02811  6.612(4)    -    Alicaforsen sodium (USAN)
D00546  8.165(2)  6.613  Suprane (TN), Desflurane (JAN/USP/INN)
D08170  7.346(2)  6.615  Melatonina (TN), Melatobel (TN), Melatonin (JAN)
D00696  7.229(2)  6.616  Versed (TN), Midazolam hydrochloride (USAN)
D01953  6.617(3)  8.860  Estradiol benzoate (JP17/USP), Ovahormon (TN)
D04465  6.617(3)  8.860  Lutes (TN), Hydroxyprogesterone caproate and estradiol benzoate
D02768  6.618(3)    -    Adefovir (USAN/INN)
D01069  6.618(3)    -    Inhibace (TN), Cilazapril (USAN), Cilazapril hydrate (JP17)
D07699  6.618(3)    -    Cilazapril (INN), Inhibace (TN)
D05630  6.625(2)  7.230  Betaprone (TN), Propiolactone (USAN/INN)
D00938  6.625(3)  7.430  Posture (TN), Tricalcium phosphate
D00242 10.286(2)  6.626  Benzonatate (USP/INN), Tessalon perles (TN)
D10620  8.958(3)  6.626  Bimagrumab (JAN/USAN/INN)
D06791  8.429(2)  6.626  Ephedra herb (JP17), Powdered ephedra herb (Non-JPS), Ephedrae Herba (TN)
D03145  7.574(2)  6.629  Boldenone undecylenate (USAN), Equipoise (TN)
D01276  6.629(4)    -    Atazanavir sulfate (JAN/USAN), Reyataz (TN)
D00287  6.631(3)  7.949  Cytoxan (TN), Cyclophosphamide hydrate (JP17), Neosar (TN), Cyclophosphamide (USP)
D07760  6.631(3)  7.949  Cyclophosphamide (INN), Cytoxan (TN), Cyclophosphamide (TN)
D10282  6.635(3)  9.134  Tarka (TN), Trandolapril and verapamil hydrochloride
D00383  6.635(3)    -    Mavik (TN), Trandolapril (JAN/USP/INN)
D10223  6.639(5)  6.635  Ibrutinib (JAN/USAN), Imbruvica (TN)
D02102  7.531(2)  6.635  Dolophine hydrochloride (TN), Methadone hydrochloride (JAN/USP)
D08195  7.531(2)  6.635  Methadone (BAN)
D10354  7.457(3)  6.635  Dupilumab (genetical recombination) (JAN), Dupilumab (USAN), Dupixent (TN)
D10280  6.636(3)  9.861  Fosinopril and hydrochlorothiazide, Fosinopril sodium and hydrochlorothiazide (TN)
D00622  6.636(3)    -    Monopril (TN), Fosinopril sodium (USP)
D07992  6.636(3)    -    Fosinopril (INN), Monopril (TN)
D03846  7.313(2)  6.637  Tecfidera (TN), Dimethyl fumarate (JAN/USAN)
D02503  6.642(4)  8.702  Timentin (TN), Clavulanic acid and ticarcillin, Augpenin (TN)
D02747  6.642(3)    -    Peginterferon alfa-2a (genetical recombination) (JAN), Peginterferon alfa-2a (USAN/INN), Pegasys (TN)
D11580  6.643(3)    -    Perindopril arginine
D11787  6.644(2)    -    Ceralasertib (USAN/INN)
D04402  6.645(3)  7.728  Sodium bicarbonate and anhydrous monobasic sodium phosphate, New Lecicarbon (TN)
D08815  6.645(3)    -    Visiclear (TN), Z-521, Monobasic sodium phosphate monohydrate and dibasic sodium phosphate anhydrous
D06391  6.645(3)    -    Monobasic calcium phosphate hydrate (JP17)
D07131  6.938(2)  6.651  Ethyl biscoumacetate (INN)
D10071  7.151(4)  6.651  Ixekizumab (genetical recombination) (JAN), Ixekizumab (USAN), Taltz (TN)
D00340  6.652(2)  9.861  Hydrochlorothiazide (JP17/USP/INN), Esidrix (TN), Microzide (TN)
D04265  6.652(2)  9.861  Reserpine, hydralazine hydrochloride and hydrochlorothiazide
D10267  6.652(2)  9.861  Methyldopa and hydrochlorothiazide (TN), Methyldopa and hydrochlorothiazide
D10269  6.652(2)  9.861  Dyazide (TN), Hydrochlorothiazide and triamterene
D10270  6.652(2)  7.513  Aldactazide (TN), Spironolactone and hydrochlorothiazide
D10271  6.652(2)  9.365  Amiloride hydrochloride and hydrochlorothiazide (TN), Amiloride hydrochloride and hydrochlorothiazide
D10273  6.652(2)  9.861  Lopressor hct (TN), Metoprolol and hydrochlorothiazide, Dutoprol (TN)
D10274  6.652(2)  9.595  Propranolol hydrochloride and hydrochlorothiazide, Propranolol hydrochloride and hydrochlorothiazide (TN), Inderide (TN)
D10275  6.652(2)  9.861  Bisoprolol fumarate and hydrochlorothiazide, Ziac (TN)
D10277  6.652(2)  9.861  Vaseretic (TN), Enalapril maleate and hydrochlorothiazide
D10279  6.652(2)  8.812  Lotensin hct (TN), Benazepril hydrochloride and hydrochlorothiazide
D10281  6.652(2)  9.861  Moexipril hydrochloride and hydrochlorothiazide, Uniretic (TN)
D10283  6.652(2)  9.861  Eprosartan mesylate and hydrochlorothiazide, Teveten htc (TN)
D10284  6.652(2)  8.589  Olmesartan medoxomil and hydrochlorothiazide, Benicar hct (TN)
D10289  6.652(2)  9.861  Aliskiren hemifumarate and hydrochlorothiazide, Tekturna hct (TN)
D09567  6.653(3)  8.575  Ulipristal (USAN/INN)
D02326  6.655(2)  6.921  Cysteine hydrochloride (USP), Elcys (TN), L-Cysteine hydrochloride hydrate (JP17)
D08748  6.655(2)  6.921  Liver hydrolysate, cysteine hydrochloride, choline bitartrate, inositol and cyanocobalamin, Proheparum (TN)
D02124  6.655(2)  6.700  Penciclovir sodium (USAN)
D05407  6.655(2)  6.700  Penciclovir (USAN/INN), Denavir (TN)
D11137  6.656(2)  7.921  Larotrectinib (USAN/INN)
D10450  6.657(2)  8.603  Alectinib hydrochloride (JAN), Alecensa (TN)
D10542  6.657(2)  8.603  Alectinib (USAN/INN)
D04519  6.657(2)  7.073  Incyclinide (USAN), Metastat (TN)
D11757  8.550(4)  6.657  Inebilizumab-cdon, Inebilizumab (genetical recombination) (JAN), Uplinza (TN), Inebilizumab (USAN/INN)
D04553  6.662(2)    -    Interferon alfa-n3 (USAN), Alferon N (TN)
D00742  6.662(2)  7.721  Etanercept (genetical recombination) (JAN), Etanercept (genetical recombination) [Etanercept biosimilar 2] (JAN), Etanercept (genetical recombination) [Etanercept biosimilar 1] (JAN), Enbrel (TN), Etanercept (USAN/INN)
D04104  6.665(3)  7.943  Implanon (TN), Etonogestrel (USAN/INN)
D07450  6.667(2)  7.593  Amlodipine (USP/INN), Norvasc (TN)
D11069  6.667(2)  7.593  Amlobenz (TN), Amlodipine and benazepril, Lotrel (TN)
D11705  6.667(2)  7.593  Amlodipine and celecoxib, Consensi (TN)
D09332  6.670(3)    -    Denenicokin (USAN/INN)
D00217  7.789(2)  6.672  Acetaminophen (JP17/USP), Tylenol (TN), Paracetamol (INN)
D00846  7.789(2)  6.672  Vicodin (TN), Hydrocodone bitartrate and paracetamol, Hydrocodone bitartrate and acetaminophen
D07666  6.846(3)  6.676  Cetylpyridinium (DCF)
D02040  6.677(3)  6.872  Pralmorelin dihydrochloride (USAN), GHRP (TN), Pralmorelin hydrochloride (JAN)
D09166  7.064(2)  6.678  Codonopsis root (JP17), Codonopsis radix
D00895  6.679(5)    -    Delavirdine mesylate (USAN), Rescriptor (TN), Delavirdine mesilate (JAN)
D10225  6.680(3)  7.463  Sacubitril (JAN/USAN/INN)
D00190  7.014(2)  6.682  Rutin hydrate (JAN), Rutin trihydrate
D04675  7.014(2)  6.682  Esberiven (TN), Melilot extract and rutin hydrate
D08499  7.014(2)  6.682  Rutoside (INN), Venoruton (TN), Rutin
D01886  6.683(3)    -    Pandel (TN), Hydrocortisone butyrate propionate (JAN), Hydrocortisone buteprate, Hydrocortisone probutate (USAN)
D07726  6.687(3)  9.709  Clomifene (TN), Clomiphene, Clomifene (INN)
D10814  6.789(3)  6.692  Glecaprevir (USAN/INN)
D10815  6.789(3)  6.692  Glecaprevir hydrate (JAN)
D11014  7.026(3)  6.692  Glecaprevir and pibrentasvir, Mavyret (TN)
D11052  6.692(5)    -    Risankizumab (genetical recombination) (JAN), Risankizumab (USAN/INN), Skyrizi (TN), Risankizumab-rzaa
D06670  6.697(2)  7.183  Selzentry (TN), Maraviroc (JAN/INN)
D02547  6.699(2)    -    Pamaquine, Plasmoquine [veterinary] (TN)
D08083  7.560(2)  6.702  Vedolizumab (genetical recombination) (JAN), Vedolizumab (USAN), Entyvio (TN)
D10948  6.703(3)    -    Larcaviximab (USAN), ZMapp (TN)
D09710  6.703(2)  8.457  Edoxaban (USAN/INN)
D11148  6.991(2)  6.704  Berzosertib (USAN/INN)
D01892  7.635(2)  6.704  Epadel S (TN), Icosapent ethyl (USAN), Ethyl icosapentate (JP17), Vascepa (TN)
D07711  6.706(3)  6.858  Clavulanic acid (INN), Clavulox (TN), CVA
D00736  6.706(3)    -    Trientine dihydrochloride, Syprine (TN), Trientine hydrochloride (JP17/USP)
D08237  6.836(2)  6.712  Avelox IV (TN), Moxifloxacin (INN)
D11618  6.836(2)  6.712  Moxifloxacin hydrochloride monohydrate
D08061  7.645(3)  6.714  Icosapent (INN), Eicosapentaenoic acid
D00211  6.721(4)  8.943  Rifampicin (JP17/INN), Rifadin (TN), Rifampin (USP), Rimactane (TN)
D08479  6.721(4)  8.943  Rifadine (TN), Rifampicin sodium
D04023  6.724(4)  9.866  Erlotinib hydrochloride (JAN/USAN), Tarceva (TN)
D00237  6.725(2)  9.007  Ridaura (TN), Auranofin (JP17/USAN/INN)
D10064  6.730(4)  8.236  Dabrafenib (USAN)
D00108  7.347(2)  6.732  Cadex (TN), Iodine (JP17/USP)
D03283  7.347(2)  6.732  Iodine and cadexomer, Iodosorb (TN), Cadexomer iodine (USAN)
D04838  7.347(2)  6.732  Iodine zinc iodide for Kantop (TN), Zinc iodide and iodine
D04875  7.347(2)  6.732  Zinc sulfate, iodine, sodium iodine and glycerin, Neoglycerol (TN)
D07709  7.347(2)  6.732  Polyvinyl alcohol and iodine, PA iodo (TN)
D08787  6.732(3)  6.864  Freeze-dried pepsin treated human normal immunoglobulin, Globulin (TN)
D01179  6.732(4)  9.425  Cymbalta (TN), Yentreve (TN), Duloxetine hydrochloride (JAN/USP)
D06795  6.735(3)    -    Saussureae radix (TN), Saussurea root (JP17)
D08066  6.736(4)  7.609  Imatinib (INN), Glamox (TN)
D00182  6.736(3)    -    Norethisterone (JP17), Norethindrone (USP), Primolut-N (TN), Camila (TN), Micronor (TN)
D04462  6.736(3)    -    Necon (TN), Norethisterone and mestranol
D00076  6.736(2)  8.201  Norepinephrine (INN), Nor adrenalin (TN), Noradrenaline (JP17)
D09794  6.736(2)  8.201  Norepinephrine hydrochloride (JAN), (+/-)-Noradrenaline hydrochloride
D00777  6.871(3)  6.740  Symmetrel (TN), Osmolex er (TN), Gocovri (TN), Amantadine hydrochloride (JP17/USP)
D07441  6.888(2)  6.740  Amantadine (INN)
D10816  6.740(2)  7.602  Pibrentasvir (JAN/USAN/INN)
D02517  7.721(2)  6.741  Sulfamethoxypyrimidine, Sulla (TN), Sulfametoxydiazine (INN), Sulfameter (USAN)
D01029  6.743(3)  9.071  Aggrastat (TN), Tirofiban hydrochloride hydrate, Tirofiban hydrochloride (USAN)
D00015  6.744(4)  8.340  Glutamine (USP), Nutrestore (TN), L-Glutamine (JP17), Levoglutamide, Endari (TN)
D03923  6.796(2)  6.746  Dusting powder, absorbable (USP)
D04305  6.796(2)  6.746  Gauze, absorbent
D06428  6.796(2)  6.746  Gelatin film, absorbable, Gelfilm (TN)
D06429  6.796(2)  6.746  Gelfoam (TN), Gelatin sponge, absorbable
D00903  7.439(2)  6.747  Cataflam (TN), Cambia (TN), Diclofenac potassium (USP), Zipsor (TN)
D00008  6.818(2)  6.747  Oxyfull (TN), Oxydol (JP17), Hydrogen peroxide (USP)
D00325  8.236(2)  6.752  Vanos (TN), Fluocinonide (JP17/USP/INN), Lidex (TN)
D09661  7.038(2)  6.752  Rintatolimod (USAN/INN)
D11648  7.467(2)  6.753  Volanesorsen (USAN/INN), Waylivra (TN)
D11650  7.467(2)  6.753  Volanesorsen sodium (USAN), Waylivra (TN)
D09720  6.753(2)  7.679  Rekambys (TN), Rilpivirine (JAN/USAN/INN)
D09958  6.753(2)  7.679  Edurant (TN), Rilpivirine hydrochloride (JAN/USAN)
D11966  6.753(2)  7.679  Cabotegravir and rilpivirine, Cabenuva (TN)
D00443  6.755(2)  7.513  Spironolactone (JP17/USP/INN), Aldactone (TN)
D08130  6.756(4)    -    Lisdexamfetamine (INN)
D09385  6.757(2)    -    Rambazole (TN), Talarozole (USAN/INN)
D01062  6.761(2)  6.773  Sprol (TN), Cetylpyridinium chloride hydrate (JAN), Cetylpyridinium chloride (USP)
D04881  6.761(2)  6.773  Aluminum chloride, cetylpyridinium chloride and Lidocaine, Dental TDZ (TN)
D08260  6.763(3)    -    Neomycin (INN), Neomycin, Kaomycine (TN), Fradiomycin
D10826  6.764(3)  7.359  Qtern (TN), Saxagliptin and dapagliflozin
D10061  6.764(3)    -    Brodalumab (USAN), Siliq (TN), Brodalumab (genetical recombination) (JAN)
D02011  6.764(3)  9.411  Flavin adenine dinucleotide sodium (JP17), FAD sodium, FAD (TN)
D00005  6.764(3)    -    Adeflavin (TN), Flavin adenine dinucleotide (JAN)
D04902  6.764(3)    -    Ribomin S (TN), Flavin adenine dinucleotide sodium and pyridoxal phosphate
D04959  6.764(3)    -    Adelavin (TN), Liver extract and flavin adenine dinucleotide sodium, Liver extract and flavin adenine dinucleotide
D01724  6.768(2)  7.151  Aluminum potassium sulfate (TN), Alum, potassium (USP), Aluminum potassium sulfate hydrate (JP17)
D02842  6.768(2)  7.151  Alum, ammonium (USP)
D05531  6.768(2)  7.151  Rhus tox antigen (TN), Poison ivy extract, alum precipitated (USAN), Alum precipitated poison ivy extract
D04040  6.774(2)  8.446  Eprosartan (USAN/INN)
D01977  6.774(2)  9.191  Gefitinib (JAN/USAN/INN), Iressa (TN)
D10210  6.775(4)  8.518  Rifater (TN), Rifampin, isoniazid and pyrazinamide
D11578  6.775(4)  9.545  Rifampin and isoniazid
D11042  6.776(3)  7.774  Tezacaftor and ivacaftor, Symdeko (TN)
D04765  6.776(3)    -    Lofexidine hydrochloride (USAN), Lucemyra (TN), Lofexidine (TN)
D02802  7.305(2)  6.778  Lemtrada (TN), Alemtuzumab (USAN/INN), Alemtuzumab (genetical recombination) (JAN), Campath (TN)
D00620  6.779(2)  8.812  Lotensin (TN), Benazepril hydrochloride (JAN/USP)
D07499  6.779(2)  8.812  Benazepril Sandoz (TN), Benazepril (INN), Forteekor [veterinary] (TN)
D03865  6.781(2)    -    Dirithromycin (USP/INN), Dynabac (TN)
D00181  7.055(2)  6.781  Argatroban (USP), Argatroban hydrate (JP17), Novastan (TN)
D00901  6.781(2)  8.754  Rimantadine hydrochloride (USP), Flumadine (TN)
D08483  6.781(2)  8.754  Rimantadine (INN)
D02194  6.782(3)  6.835  Cilastatin sodium (JP17/USP), CS
D08747  6.785(2)  6.817  Licothion (TN), DL-Methionine and glycyrrhizic acid
D08956  6.791(2)  6.792  Omacetaxine mepesuccinate (USAN), Synribo (TN), Homoharringtonine
D06547  6.793(2)  7.631  TT, Tetanus toxoid (USP)
D10551  6.794(2)    -    Ceritinib (JAN/USAN/INN), Zykadia (TN)
D00633  6.795(3)  8.983  Intropin (TN), Dopamine hydrochloride (JP17/USP), Actopamin (TN)
D07870  6.795(3)  8.983  Dopamine (INN), Medopa (TN)
D05794  6.795(2)  7.513  Salts, rehydration (USP)
D07554  6.795(2)  7.171  Acetylcysteine Sodium (TN), L-Cysteine, N-acetyl-, sodium salt, Acetylcysteine sodium salt
D01843  8.489(2)  6.795  Fludeoxyglucose (18F) (JAN/INN), 18DG, Fludeoxyglucose F 18 (USP), Fludeoxyglucose F 18 (TN)
D04862  7.082(2)  6.795  meso-Nordihydroguaiaretic acid, Masoprocol (USAN/INN), Actinex (TN)
D11390  7.499(3)  6.795  Daunorubicin and cytarabine, Vyxeos (TN)
D11457  8.333(2)  6.795  Fenebrutinib (USAN/INN)
D00106  6.799(2)  7.230  Prostacyclin, Prostaglandin I2, Epoprostenol (TN), Epoprostenol (USAN/INN)
D03478  6.799(4)    -    Ovidrel (TN), Choriogonadotropin alfa (USAN/INN), Choriogonadotropin alfa (genetical recombination) (JAN)
D05295  6.811(3)  8.167  Smallpox vaccine (USP)
D10481  7.420(2)  6.811  Nintedanib (USAN/INN), Ofev (TN)
D04358  6.812(3)  9.022  Golimumab (genetical recombination) (JAN), Golimumab (USAN/INN), Simponi (TN)
D10550  6.812(3)    -    Festinavir, Censavudine (USAN)
D08302  6.812(3) 10.361  Ornithine (INN)
D01915  6.815(4)  7.812  Crestor (TN), Rosuvastatin calcium (JAN/USAN), ZD 4522
D07704  6.815(2)  8.176  Citadur (TN), Citalopram (USP/INN)
D07705  6.815(2)  8.176  Citalopram hydrochloride, Seropram (TN)
D10899  6.815(4)    -    Voxilaprevir (USAN/INN)
D08707  6.815(3)  7.728  l-Menthol, gentian, scopolia extract powder and sodium bicarbonate, Bellsan (TN)
D10080  6.818(3)    -    Sirukumab (USAN), Sirukumab (genetical recombination) (JAN)
D00277  6.820(2)  8.531  Cleocin (TN), Clindamycin (USAN/INN)
D02132  6.820(2)  8.531  Cleocin hydrochloride (TN), Dalacin (TN), Clindamycin hydrochloride (JP17/USP)
D00412  6.820(2)    -    Zalcitabine (JAN/USP/INN), Hivid (TN)
D01235  6.821(2)  8.180  Josamycin (TN), Josamycin (JP17/USAN/INN), Leucomycin A3, JM
D10873  6.821(4)    -    Eucrisa (TN), Crisaborole (USAN/INN)
D10483  7.537(2)  6.823  Plegridy (TN), Peginterferon beta-1a (USAN/INN)
D00750  6.824(5)  8.556  Levamisole hydrochloride (USP), Ergamisol (TN)
D01264  6.824(5)  9.159  Daunorubicin hydrochloride (JP17/USP), Cerubidine (TN)
D05237  6.826(4)  8.386  Ointment, hydrophilic (USP), Hydrophilic ointment, Hydrophilic ointment (TN), Hydrophilic cream (JP17)
D01994  6.828(5)    -    Gasmotin (TN), Mosapride citrate dihydrate, Mosapride citrate hydrate (JP17)
D01204  6.829(3)  8.589  Olmesartan medoxomil (JP17/USP), Olmetec (TN), Benicar (TN)
D09594  6.829(3)  8.589  Rezaltas (TN), Olmesartan medoxomil and azelnidipine
D00255  7.586(2)  6.831  Coreg (TN), Artist (TN), Carvedilol (JP17/USP/INN)
D00765  6.833(4)    -    Rocuronium bromide (JAN/USP/INN), Zemuron (TN)
D04097  6.833(3)    -    Etilevodopa (USAN/INN)
D03779  6.833(3)    -    Dibotermin alfa (USAN/INN)
D08918  6.834(4)  9.194  Falimarev (USAN), CEA, MUC-1, fowlpox virus
