# Supplementary material for: A novel method of literature mining to identify candidate COVID-19 drugs
Source: Bioinform Adv. 2021 Jul 22;1(1):vbab013. doi: 10.1093/bioadv/vbab013 (PMC9710631; doi:10.1093/bioadv/vbab013)
Supplement: vbab013_Supplementary_Data [file vbab013_supplementary_data.zip › TableS2_muramatsu.rtf]

Table S2. Distances between COVID-19 and each drug calculated by the Dice index method (first 1000 items).
Code: KEGG code, D_id: indirect (predicted) distance, D_d: direct (co-existing) distance

Code    D_id(Dpt)  D_d   Name
---------------------------------------------------------------------------------------------------------------
D08050  4.024(2)  3.224  Polirreumin (TN), Hydroxychloroquine (INN)
D11472  4.045(2)  3.479  Veklury (TN), Remdesivir (JAN/USAN)
D01425  4.698(2)  3.937  Lopinavir (JAN/USP/INN)
D02498  4.698(2)  3.937  Kaletra (TN), Lopinavir and ritonavir
D02596  5.026(2)  3.941  Tocilizumab (genetical recombination) (JAN), Actemra (TN), Tocilizumab (USAN/INN)
D03344  5.283(2)  4.100  Silver protein (TN), Silver protein, mild, Silver protein (JP17)
D00427  4.858(2)  4.120  Norvir (TN), Ritonavir (JAN/USP/INN)
D10582  4.858(2)  4.120  Dasabuvir, ombitasvir, paritaprevir and ritonavir, Viekira pak (TN)
D10745  4.858(2)  4.120  Technivie (TN), Ombitasvir, paritaprevir and ritonavir
D03043  5.901(2)  4.140  Air (TN), Air, medical (USP)
D02366  4.937(2)  4.152  Chloroquine (USP/INN)
D03469  4.937(2)  4.152  Aralen hydrochloride (TN), Chloroquine hydrochloride (USP)
D06458  5.121(2)  4.284  Bay gam (TN), Globulin, immune (USP), Gamma globulin (TN), Hyqvia (TN), Human normal immunoglobulin (JP17)
D10211  6.110(2)  4.312  Rotavirus vaccine, live, oral, pentavalent, Rotateq (TN)
D02134  5.164(2)  4.359  Zithromac (TN), Zmax (TN), Azithromycin hydrate (JP17), Azimycin (TN), Azithromycin dihydrate
D06390  5.164(2)  4.359  Zmax (TN), Azithromycin (USP)
D07486  5.164(2)  4.359  Azithromycin (INN), Azithromycin (TN), Azasite (TN)
D05864  6.216(2)  4.378  Sodium monofluorophosphate (USP), Aim (TN)
D04550  5.864(2)  4.548  Semilente iletin (TN), Insulin zinc, prompt (USP)
D09153  6.074(2)  4.609  Elder
D09537  5.204(2)  4.675  Avigan (TN), Favipiravir (JAN/USAN/INN)
D00003  6.354(2)  4.819  Oxygen (JP17/USP)
D03841  6.354(2)  4.819  Nitrous oxide and oxygen, Anesoxyn (TN)
D04319  6.206(2)  4.838  Glaze, pharmaceutical (NF)
D10156  6.364(2)  4.882  Evenity (TN), Romosozumab-aqqg, Romosozumab (USAN), Romosozumab (genetical recombination) (JAN)
D06505  6.675(2)  4.892  Starch, topical (USP)
D08150  6.998(2)  4.946  Lufenuron (USP/INN), Program [veterinary] (TN)
D08266  6.353(2)  4.956  Pizensy (TN), Lactitol (NF/INN), Importal (TN)
D04548  5.262(2)  4.974  Insulin, neutral (USAN), Neutral insulin injection (INN)
D00548  7.022(2)  4.978  Amidate (TN), Etomidate (USP/INN)
D10553  5.008(3)  9.146  Dasabuvir (USAN/INN)
D10581  5.008(3)  9.146  Dasabuvir sodium hydrate, Dasabuvir sodium monohydrate, Exviera (TN)
D04482  5.042(2)  6.081  Ethinylestradiol and levonorgestrel, Trivora (TN), Lo/ovral (TN), Ange (TN), Levora (TN), Seasonique (TN), Triphasil-21 (TN)
D02844  6.495(2)  5.075  Aluminum carbonate, basic (USAN), Basaljel (TN)
D03678  6.495(2)  5.075  Fuchsin, basic (USP)
D10598  5.158(4)    -    Ombitasvir hydrate (JAN), Ombitasvir heminonahydrate
D10576  5.158(4)    -    Ombitasvir (USAN)
D00111  5.536(2)  5.182  Lactate (TN), Lactic acid (JP17/USP)
D00936  5.536(2)  5.182  Prequist powder (TN), Calcium lactate (USP)
D02183  5.536(2)  5.182  Mediject L (TN), Sodium lactate (JAN/USP)
D02254  5.536(2)  5.182  Calcium lactate pentahydrate, Calcium lactate hydrate (JP17), Calcium lactate (TN)
D04969  6.921(2)  5.196  Wax, white (NF), White beeswax (JP17), White wax (TN)
D05239  6.921(2)  5.196  White ointment (JP17), Ointment, white (USP)
D05304  6.921(2)  5.196  Petrolatum, white (USP), White petrolatum (JP17), Moroline (TN)
D03138  6.710(2)  5.202  Blood cells, red (USP)
D10580  5.208(4)  8.051  Paritaprevir (USAN/INN)
D10597  5.208(4)  8.051  Paritaprevir dihydrate, Paritaprevir hydrate (JAN)
D02112  5.974(2)  5.211  Parnaparin sodium (INN), Heparin sodium (JP17/USP/INN), Liquemin sodium (TN)
D04427  5.974(2)  5.211  Nadroparin calcium (INN), Calciparine (TN), Calciparin, Heparin calcium (JP17)
D07510  5.974(2)  5.211  Heparin (TN), Semuloparin (USAN), Enoxaparin, Heparin (BAN), Parnaparin, Bemiparin, Nadroparin, Adomiparin (USAN)
D07422  5.993(2)  5.240  Fibrogammin (TN), Fibrinolysin, human, Fibrinolysin (human) (INN)
D00292  6.323(2)  5.278  Maxidex (TN), Dexamethasone (JP17/USP/INN), Decadron (TN)
D02273  6.323(2)  5.278  Stmerin D (TN), Isoproterenol sulfate, dexamethasone and atropine methybromide
D08733  6.323(2)  5.278  Glymesason (TN), Dexamethasone and glyteer
D10296  6.323(2)  5.278  Ciprodex (TN), Ciprofloxacin hydrochloride and dexamethasone
D11177  6.323(2)  5.278  Maxitrol (TN), Dexasporin (TN), Dexamethasone, neomycin sulfate and polymyxin B sulfate
D11178  6.323(2)  5.278  Tobramycin and dexamethasone, Tobradex (TN)
D02916  6.594(2)  5.291  Ammonia, Ammonia solution, strong (NF)
D05765  6.594(2)  5.291  Rose water, strong (NF), Rose water , stronger (NF)
D00366  5.312(2)    -    Diapid (TN), Lypressin (USAN/INN)
D04182  6.589(2)  5.330  Fibrinogen I 125 (USAN), Fibrinogen (125I) (INN), Ibrin (TN)
D05439  6.619(2)  5.334  Perflubron (USP/INN), Imagent (TN)
D00584  5.336(4) 10.132  Fluorouracil (JP17/USP/INN), Fluoroplex (TN), Carac (TN), 5-FU (TN), Adrucil (TN)
D10528  5.336(2)  5.485  Aldosterone (INN)
D03237  6.447(2)  5.356  Aluminum silicate, natural (JAN), Natural aluminum silicate (JP17), Adsorbin (TN)
D06544  6.098(2)  5.377  Corticotropin, repository (USP), Cortigel (TN)
D00150  5.902(3)  5.418  Angiotensin II (INN)
D02014  5.902(3)  5.418  Angiotensin II (human type) (JAN), Delivert (TN), Angiotensin II (USAN)
D04296  6.909(2)  5.424  Asthma (TN), Diprophylline, methoxyphenamine hydrochloride, noscapine and chlorpheniramine maleate
D00357  5.426(2)  7.532  Cozaar (TN), Losartan potassium (JP17/USP)
D07895  5.426(2)  7.532  Hydrochlorothiazide and losartan potassium, Losarhyd (TN), Losartan potassium and hydrochlorothiazide (JP17), Hyzaar (TN)
D08146  5.426(2)  7.532  Losartic (TN), Losartan (INN)
D01202  6.795(2)  5.431  Artes (TN), Melinamide (JAN)
D03143  6.163(2)  5.442  Blood, whole (USP)
D00423  5.443(2)  5.632  Ribasphere (TN), Copegus (TN), Rebetol (TN), Ribavirin (JP17/USP/INN), Virazole (TN)
D00407  6.139(2)  5.444  Medrol (TN), Methylprednisolone (JP17/USP/INN)
D04250  6.139(2)  5.444  Fradiomycin sulfate and methylprednisolone, Neo medrol EE (TN)
D12027  5.450(2)  7.753  Civet
D07943  6.536(2)  5.476  Fendiline hydrochloride, Sensit (TN)
D04543  7.047(2)  5.504  Insulin human zinc, extended (USP)
D04549  7.047(2)  5.504  Insulin zinc, extended (USP), Ultralente iletin (TN)
D01732  6.558(2)  5.512  Sodium sulfate, dried (TN), Sodium sulfate, dried (JAN), Anhydous sodium sulfate (JP17)
D02416  6.558(2)  5.512  Aluminum hydroxide (USP), Aluminum hydroxide, dried (USP), Dried aluminum hydroxide gel (JP17), Dialume (TN), ALterna GEL (TN)
D04172  6.558(2)  5.512  Tetucur (TN), Fero-gradumet (TN), Ferrous sulfate, dried (USP), Ferrous sulfate hydrate
D04364  6.456(2)  5.512  Kolantyl (TN), Dicyclomine hydrochloride, aluminium hydroxide, dried and magnesium oxide
D04380  6.558(2)  5.512  Yeast, dried, Dried yeast (JP17), Ebios (TN)
D05283  6.558(2)  5.512  Dried sodium carbonate (JP17), Sodium carbonate (NF), Sodium carbonate, dried
D08708  6.558(2)  5.512  NS (TN), Licase (alpha Amylase), aluminum hydroxide, dried, magnesium carbonate, sodium bicarbonate and precipitated calcium carbonate, AM (TN)
D08793  6.558(2)  5.512  Human blood-coagulation factor eight inhibitor bypassing activity complex, dried, Feiba (TN)
D08794  6.558(2)  5.512  Human blood-coagulation factor IX complex, dried, PPSB-HT (TN)
D08802  6.419(3)  5.512  Human blood-coagulation factor XIII fraction, dried, Fibrogammin P (TN)
D08816  6.558(2)  5.512  Niflec (TN), Potassium chloride, sodium chloride, sodium bicarbonate and sodium sulfate, dried
D02934  6.531(2)  5.519  Anakinra (USAN/INN), Kineret (TN)
D04664  7.020(2)  5.530  Lanolin, modified (USP), Hydrous lanolin (JP17), Wool wax, Lanolin (TN)
D07471  5.545(2)  7.259  Reyataz (TN), Atazanavir (INN)
D10753  5.545(2)  7.241  Evotaz (TN), Atazanavir and cobicistat
D08682  5.933(2)  5.549  Choice (TN), Warfarine, Warfarin (INN)
D11032  5.550(2)  8.435  Plitidepsin (INN)
D03045  6.614(2)  5.552  Parepectolin (TN), Attapulgite, activated (USP)
D03251  6.614(2)  5.552  Medicinal carbon (JP17), Charcoal, activated (USP), Medicinal carbon (TN)D00429  5.563(2)  7.729  Saquinavir (JAN/USP/INN), Fortovase (TN)
D05043  6.966(2)  5.578  Mineral oil, light (NF), Light liquid paraffin (JP17)
D07227  5.586(2)    -    Ornipressin (INN), POR 8 Sandoz (TN)
D04133  6.969(2)  5.589  Fat, hard (NF)
D05355  5.601(2)  6.239  Papain (USP), Caroid (TN)
D06298  7.088(2)  5.618  Vidarabine (JAN), Vidarabine anhydrous, Armes (TN), ARA-A
D03600  6.769(2)  5.618  Creatinine (NF)
D03656  5.645(2)  6.463  Darunavir (USAN/INN)
D10832  5.645(2)  6.463  Darunavir and cobicistat, Rezolsta (TN), Prezcobix (TN)
D11382  5.645(2)  6.463  Symtuza (TN), Darunavir, cobicistat, emtricitabine and tenofovir alafenamide
D08306  6.148(2)  5.658  Agucort (TN), Oseltamivir (INN)
D00016  5.986(2)  5.666  L-Serine (JP17), Serine (USP)
D10558  6.573(3)  5.695  Umifenovir (INN)
D03833  5.699(2)  8.140  Indinavir hydrate, Indinavir (USAN)
D02026  6.140(2)  5.716  Magnesium aspartate, Magnesium L-aspartate (JAN)
D04948  6.140(2)  5.716  Aspara K (TN), Potassium L-aspartate (JAN), L-Aspartate potassium, Potassium aspartate
D04952  6.140(2)  5.716  Potassium aspartate and magnesium aspartate (JAN/USAN), Aspara (TN)
D05258  6.236(2)  5.742  Gonadotrophin, serum (JAN/INN), Anteron (TN), Serum gonadotrophin (JAN)
D00448  5.766(2)  8.059  Salazosulfapyridine (JP17), Azulfidine (TN), Sulfasalazine (USP/INN)
D10226  5.777(3) 10.413  Entresto (TN), Sacubitril mixture with valsartan, Sacubitril valsartan sodium hydrate (JAN)
D10193  6.692(2)  5.795  Rotarix (TN), Live attenuated human rota virus vaccine, oral
D00012  6.020(2)  5.805  Alanine (USP), L-Alanine (JP17)
D00068  7.168(2)  5.807  Dehydrated ethanol, Alcohol (USP), Anhydrous ethanol (JP17), Dehydrated ethanol (TN), Ethyl alcohol
D02799  7.168(2)  5.807  Alcohol, rubbing (USP), Alcolo (TN)
D04732  7.168(2)  5.807  Isopropanol and methylated alcohol, Alcohol (TN)
D10301  7.168(2)  5.807  Chlorhexidine gluconate and alcohol, Prevantics (TN)
D07508  6.717(2)  5.812  Mediator (TN), Benfluorex hydrochloride
D01738  6.892(2)  5.843  Definity (TN), Perflutren (JAN/USAN/INN)
D00534  6.934(2)  5.894  Malathion (USP), Ovide (TN)
D05424  6.944(3)  5.894  Human serum albumin, Albumin (TN), Human serum albumin (genetical recombination) (JAN)
D00564  5.933(2)  7.124  Coumadin (TN), Jantoven (TN), Warfarin sodium (USP)
D01280  5.933(2)  7.124  Warfarin potassium (JP17), Athrombin-K (TN)
D04371  7.070(2)  5.936  Soap, green (USP), Green soap
D00251  5.937(2)  8.476  Captopril (JP17/USP/INN), Capoten (TN), Apopril (TN)
D10276  5.937(2)  8.476  Captopril and hydrochlorothiazide, Capozide (TN)
D00804  6.283(2)  5.949  Soolantra (TN), Stromectol (TN), Sklice (TN), Ivermectin (JAN/USP/INN)
D06773  7.091(2)  5.950  Lonicera leaf and stem (JP17)
D11581  5.950(3)  8.258  Prestalia (TN), Perindopril and amlodipine
D00753  5.951(4)  7.121  Rapamune (TN), Sirolimus (JAN/USAN/INN), Rapamycin (TN)
D00024  7.129(2)  5.952  Sulfur (JP17), Sulfur, sublimed (USP), Sulfur, precipitated (USP), Bensulfoid (TN), Sastid (TN)
D00932  7.129(2)  5.952  Calcium carbonate (USP), Precipitated calcium carbonate (JP17), Cal-sup (TN), Calcium carbonate, precipitated (JAN)
D08259  5.953(2)  7.177  Viracept (TN), Nelfinavir (INN)
D03674  7.020(2)  5.958  Lovenox (TN), Enoxaparin sodium (JAN/USP/INN)
D08914  5.968(2)    -    Eribulin mesilate (JAN), Halaven (TN), Eribulin mesylate (USAN)
D07974  5.993(4) 10.132  Fluorouracil (TN), Fluorouracil sodium salt
D01505  6.744(2)  5.994  Domin (TN), Talipexole hydrochloride (JAN)
D08446  6.744(2)  5.994  Prothipendyl hydrochloride, Dominal (TN)
D07776  6.796(2)  6.007  Daunorubicin (INN), DaunoXome (TN), DM
D05200  7.150(2)  6.027  Merital (TN), Nomifensine maleate (USAN)
D02769  6.035(5) 10.081  Adenosine phosphate (USAN/INN), Adenyl (TN)
D06177  7.287(2)  6.046  Tolbutamide sodium, sterile, Orinase diagnostic (TN)
D00896  6.060(2)  9.084  Efavirenz (JAN/USP/INN), Sustiva (TN)
D10851  6.060(2)  7.846  Atripla (TN), Efavirenz, emtricitabine and tenofovir disoproxil
D11392  6.060(2)  8.122  Symfi (TN), Efavirenz, lamivudine and tenofovir disoproxil fumarate
D00897  6.074(3)    -    Indinavir sulfate (USP), Crixivan (TN)
D02861  6.074(3)    -    Indinavir sulfate ethanolate (JAN), Crixivan (TN)
D00488  6.096(2)  9.377  Pyrimethamine (JAN/USP/INN), Daraprim (TN)
D02448  6.096(2)  9.377  Pyrimethamine and sulfadoxine, Fansidar (TN)
D10308  6.746(2)  6.098  Olumiant (TN), Baricitinib (JAN/USAN/INN)
D01118  6.110(4)    -    Parkin (TN), Ethopropazine hydrochloride, Profenamine hydrochloride (JAN), Parsidol (TN)
D03276  6.110(4)    -    Parkin (TN), Profenamine hibenzate (JAN)
D01982  6.122(3)  8.122  Viread (TN), Tenofovir disoproxil fumarate (JAN/USAN)
D02297  6.122(3)  7.846  Emtricitabine and tenofovir disoproxil, Truvada (TN)
D10756  6.122(3)  7.241  Elvitegravir, cobicistat, emtricitabine and tenofovir disoproxil, Stribild (TN)
D00517  6.127(2)  6.359  Amyl nitrite (JP17/USP), Vaporole (TN), Aspiral (TN)
D08547  7.193(2)  6.130  Sulodexide (INN), Vessel (TN)
D00276  6.136(2)  7.489  Biaxin (TN), Clarithromycin (JP17/USP/INN)
D08774  6.136(2)  7.489  Amoxicillin hydrate, clarithromycin and lansoprazole, Prevpac (TN), Lansap (TN)
D10246  6.136(2)  7.489  Omeclamox-pak (TN), Omeprazole, clarithromycin and amoxicillin
D10519  6.136(2)  7.489  Rabeprazole, amoxicillin and clarithromycin, Rabecure (TN)
D10775  6.136(2)  7.489  Vonosap (TN), Vonoprazan, amoxicillin and clarithromycin
D10571  6.185(4)  8.122  Eviplera (TN), Rilpivirine hydrochloride, tenofovir disoproxil fumarate and emtricitabine, Complera (TN)
D11395  6.185(4)  7.846  Cimduo (TN), Temixys (TN), Lamivudine and tenofovir disoproxil
D11396  6.185(4)  7.846  Doravirine, lamivudine and tenofovir disoproxil, Delstrigo (TN)
D00023  6.394(3)  6.188  Carbamide, Urea (JP17/USP), Pastaron (TN)
D01749  7.187(2)  6.188  Urea (13C) (JAN), Helicosol (TN), Urea C13 (USP)
D06074  6.190(2)  7.036  Tenofovir hydrate, Tenofovir (USAN)
D01160  6.195(3)  9.126  Saquinavir mesilate (JAN), Saquinavir mesylate (USP), Invirase (TN)
D09578  6.201(2)  6.329  Albumin human, recombinant (NF)
D08834  7.143(2)  6.208  Ohara (TN), Stomachic and digestive
D03484  6.220(2)  6.627  Chymotrypsin (JAN/USP/INN), Catarase (TN)
D00570  6.988(2)  6.226  Colchicine (JP17/USP), Colchicine (TN)
D11574  6.988(2)  6.226  Probenecid and colchicine, Col-probenecid (TN)
D00831  6.235(2)  7.811  Mefloquine hydrochloride (JP17/USP), Lariam (TN)
D04895  6.235(2)  7.811  Mefloquine (USAN/INN)
D08095  6.238(3)    -    Corlanor (TN), Coralan (TN), Corlentor (TN), Ivabradine hydrochloride (JAN/USAN), Ivabrandine hydrochloride
D00009  6.243(3)  6.396  Purified glucose (JP17), Glucose (JP17), D-Glucose
D02019  6.243(3)  6.396  Magnesium sulfate hydrate and glucose, Magnesol (TN)
D02325  6.243(3)  6.396  Glucose hydrate (JP17), Dextrose (USP), Dextrose monohydrate, alpha-D-Glucose monohydrate, Cartose (TN)
D04109  6.243(3)  6.396  Glucose and inorganic salt, Opeguard MA (TN)
D04337  6.243(3)  6.396  Glucose, liquid (JAN/NF), Glucose-40 (TN), Liquid glucose
D04963  6.243(3)  6.396  Dextron (TN), Dextran 40 and glucose
D04978  6.243(3)  6.396  ACD-A solution, Sodium citrate hydrate, citric acid hydrate and glucose, Acid -Citrate -Dextrose solution, ACD-A (TN)
D08741  6.243(3)  6.396  Adenine, sodium citrate hydrate, citric acid hydrate, glucose and potassium phosphate, monobasic, Karmi CA (TN), CPDA solution
D08742  6.243(3)  6.396  CPD solution, Sodium citrate hydrate, citric acid hydrate, glucose and potassium phosphate, monobasic, Karmi C (TN)
D08743  6.243(3)  6.396  D-Mannitol, adenine, potassium phosphate, monobasic, sodium citrate hydrate, citric acid hydrate, glucose and sodium chloride, MAP solution
D03063  6.863(2)  6.253  Tice BCG (TN), BCG vaccine (USP)
D06677  6.265(3)    -    Vitekta (TN), Elvitegravir (JAN/USAN), GS-9137
D01199  6.267(2)  8.145  Emtriva (TN), Emtricitabine (JAN/USAN/INN)
D10755  6.267(2)  7.241  Elvitegravir, cobicistat, emtricitabine and tenofovir alafenamide, Genvoya (TN)
D10835  6.267(2)  8.145  Emtricitabine and tenofovir alafenamide, Descovy (TN)
D10836  6.267(2)  8.145  Emtricitabine, rilpivirine and tenofovir alafenamide, Odefsey (TN)
D11039  6.267(2)  8.145  Biktarvy (TN), Bictegravir, emtricitabine and tenofovir alafenamide
D02305  6.280(2)  7.156  ROS, Roxadyl (TN), Rosoxacin (USAN/INN)
D10469  6.285(3)  7.369  Olysio (TN), Simeprevir sodium (JAN), Sovriad (TN)
D10578  6.285(3)  6.759  Harvoni (TN), Ledipasvir and sofosbuvir
D03383  6.291(2)    -    Carbamide peroxide (USP), Murine ear drops (TN)
D07008  6.297(2)    -    Chikujountanto
D02114  6.300(2)  7.522  Plaquenil (TN), Hydroxychloroquine sulfate (JAN/USP)
D10442  6.316(3)  9.039  Ledipasvir (USAN)
D07892  6.351(2)  9.402  Enalapril (INN), Enalapril (TN)
D10161  7.214(2)  6.360  Sarilumab (USAN), Kevzara (TN), Sarilumab (genetical receombination) (JAN)
D00672  6.373(2)  8.477  Hydroxyzine dihydrochloride, Atarax (TN), Vistaril (TN), Hydroxyzine hydrochloride (JP17/USP)
D08054  6.373(2)  8.477  Hydroxyzine (INN), Marex (TN)
D00085  6.405(2)  6.827  Insulin (JAN/USP)
D04546  6.405(2)  6.827  Insulin, dalanated (USAN)
D04547  6.405(2)  6.827  Humalog PEN (TN), Isophane insulin (aqueous suspension) (JAN), Insulin, isophane (USP), Isophane insulin human (genetical recombination) injectable aqueous suspension (JP17)
D00944  7.133(3)  6.406  Metformin hydrochloride (JP17/USP), Glucophage (TN)
D04966  7.133(3)  6.406  Metformin (USAN/INN)
D09744  7.133(3)  6.406  Metact (TN), Pioglitazone and metformin, Pioglitazone hydrochloride and metformin hydrochloride (JP17)
D10244  7.133(3)  6.406  Avandamet (TN), Metformin hydrochloride and rosiglitazone maleate
D10253  7.133(3)  6.406  Vipdomet (TN), Kazano (TN), Alogliptin benzoate and metformin hydrochloride
D10261  7.133(3)  6.406  Metformin hydrochloride and sitagliptin phosphate, Janumet (TN)
D10263  7.133(3)  6.406  Metformin hydrochloride and saxagliptin hydrochloride, Kombiglyze (TN)
D10264  7.133(3)  6.406  Jentaduet (TN), Linagliptin and metformin hydrochloride
D10265  7.133(3)  6.406  Metaglip (TN), Glipizide and metformin hydrochloride
D10266  7.133(3)  6.406  Glyburide and metformin hydrochloride, Glucovance (TN), Glibenclamide and metformin hydrochloride
D10500  7.133(3)  6.406  Metformin hydrochloride and repaglinide, Prandimet (TN)
D10586  7.133(3)  6.406  Xigduo xr (TN), Dapagliflozin and metformin
D10587  7.133(3)  6.406  Canagliflozin and metformin, Invokamet (TN)
D10743  7.133(3)  6.406  Vildagliptin and metformin hydrochloride
D10752  7.133(3)  6.406  Empagliflozin and metformin, Synjardy (TN)
D11067  7.133(3)  6.406  Segluromet (TN), Ertugliflozin and metformin
D11109  7.133(3)  6.406  Anagliptin and Metformin hydrochloride
D11711  7.133(3)  6.406  Dapagliflozin, metformin and saxagliptin, Qternmet xr (TN)
D11856  7.133(3)  6.406  Trijardy xr (TN), Empagliflozin, linagliptin and metformin hydrochloride
D02261  6.409(2)  8.116  Quinine hydrochloride (TN), Quinine hydrochloride hydrate (JP17), Quinine hydrochloride dihydrate
D08460  6.409(2)  8.116  Quinine (BAN), Kinder Quinina (TN)
D08461  6.409(2)  8.116  Quinine dihydrochloride, Quinine (TN)
D08810  6.409(2)  7.366  Quinine hydrochloride, sodium chloride, tartaric acid and sucrose
D00749  6.418(2)  8.043  Arava (TN), Leflunomide (JAN/USP/INN)
D00899  6.419(3)  9.720  Viracept (TN), Nelfinavir mesilate (JAN), Nelfinavir mesylate (USAN/INN)
D00353  6.427(2)  8.445  Epivir (TN), Lamivudine (JAN/USP/INN)
D07507  6.427(2)  8.445  Lamivudine zidovudine (TN), Zidovudine and lamivudine, Combivir (TN)
D08775  6.427(2)  8.445  Abacavir sulfate and lamivudine, Epzicom (TN)
D10600  6.427(2)  8.445  Triumeq (TN), Dolutegravir, abacavir and lamivudine
D10754  6.427(2)  8.134  Dutrebis (TN), Lamivudine and raltegravir, Lamivudine and raltegravir potassium
D10838  6.427(2)  8.445  Abacavir, lamivudine and zidovudine, Trizivir (TN)
D11521  6.427(2)  8.445  Lamivudine, nevirapine and zidovudine
D11522  6.427(2)  8.445  Dovato (TN), Dolutegravir and lamivudine
D00894  6.430(2)  8.477  Agenerase (TN), Amprenavir (JAN/USAN/INN)
D03236  7.062(2)  6.443  Aluminum silicate, synthetic (JAN), Synthetic aluminum silicate (JP17), Silicamin (TN)
D05358  7.062(2)  6.443  Paraffin, synthetic (NF)
D08240  6.809(3)  6.444  Nafamostat (INN)
D01167  6.456(2)  6.820  Magnesium oxide (JP17/USP), Magmitt (TN)
D01679  6.456(2)  6.820  Calcium oxide (JP17), Lime (USP)
D03436  6.456(2)  6.820  Cellulose, oxidized (JAN/USP), Surgicel (TN)
D08696  6.456(2)  6.820  Asgen (TN), Acetaminophen, ephedra herb, scopolia extract, caffeine and sodium benzoate and magnesium oxide
D08711  6.456(2)  6.820  Coptis rhizome, senna leaf, rhubarb, magnesium oxide and magnesium sulfate hydrate, Cetilo (TN)
D10793  6.456(2)  6.820  Prepopik (TN), Picoprep (TN), Sodium picosulfate hydrate, magnesium oxide and citric acid, anhydrous, Clenpiq (TN)
D00101  6.463(2)  9.391  Pitressin (TN), Vasopressin (JP17/USP), Vasostrict (TN)
D10081  6.464(3)  7.369  Simeprevir (JAN/USAN)
D08729  7.201(2)  6.476  Salicylic acid and zinc oxide, Zinc and salicylic acid (TN)
D08092  6.665(2)  6.484  Dilator (TN), Isoxsuprine (INN)
D02994  7.348(2)  6.486  Truximab (TN), Rituximab (genetical recombination) (JAN), Rituximab (genetical recombination) [Rituximab biosimilar 2] (JAN), Rituximab (USAN/INN), Rituximab (genetical recombination) [Rituximab biosimilar 1] (JAN), Rituxan (TN), Rituximab-pvvr, Rituximab-abbs
D11393  7.348(2)  6.486  Rituximab and hyaluronidase, Rituxan hycela (TN)
D06676  6.509(2)  8.134  MK-0518, Raltegravir (INN)
D07133  6.509(2)  8.134  Isentress (TN), Raltegravir potassium (JAN/USAN)
D04620  6.515(3)    -    Iseganan hydrochloride (USAN)
D07725  6.515(3)    -    Clomethiazole edisilate, Heminevrin (TN)
D00580  6.519(2) 10.442  Sulfadoxine (JAN/USP/INN)
D00028  6.989(2)  6.522  Concentrated glycerin (JP17), Glycerin, concentrated (JAN), Glycerin (JP17/USP), Glycerol (INN)
D08780  6.989(2)  6.522  Berinert P (TN), Human C1 inactivator, freeze-dried concentrated
D08795  6.989(2)  6.522  Human anti-thrombin III, freeze-dried concentrated, Neuart (TN)
D08796  6.989(2)  6.522  Human activated protein C, freeze-dried concentrated, Anact C (TN)
D10884  6.989(2)  6.522  Human prothrombin complex, freeze-dried concentrated
D11965  6.989(2)  6.522  alpha 1-Antitrypsin, Human alpha 1-Proteinase inibitor, freeze-dried concentrated, Lynspad (TN)
D00435  6.529(2)  9.310  Nevirapine (JAN/USP/INN), Viramune (TN)
D00140  6.530(2)  8.355  T-stat (TN), Akne-mycin (TN), Eryc (TN), Erythromycin (JP17/USP/INN), Pce (TN), Erygel (TN), Staticin (TN)
D11646  6.530(2)  8.355  Benzoyl peroxide and erythromycin, Aktipak (TN)
D05095  6.840(3)  6.534  Mycophenolate sodium (USP), Myfortic (TN)
D10366  6.539(3)  6.759  Sovaldi (TN), Sofosbuvir (JAN/USAN)
D00072  6.540(4) 10.903  Cytorest (TN), Cytochrome c (JAN)
D00522  6.551(2)  8.504  Candesartan (USAN/INN)
D00142  6.556(2)  7.082  Otrexup (TN), Methotrexate (JP17/USP/INN), Xatmep (TN)
D02115  6.556(2)  7.082  Trexall (TN), Rasuvo (TN), Methotrexate sodium
D05519  7.053(2)  6.557  Pituitrin (TN), Pituitary, posterior
D00238  6.561(2)  7.556  Azasan (TN), Imuran (TN), Azathioprine (JP17/USP/INN)
D03033  6.561(2)  7.556  Imuran (TN), Azathioprine sodium (USP)
D00041  6.569(2)  8.792  L-Threonine (JP17), Threonine (USP)
D02050  6.575(2)  7.611  Potassium phosphate, monobasic (JAN/NF)
D04400  6.575(2)  7.611  Anhydrous monobasic sodium phosphate, Sodium phosphate, monobasic (USP)
D01703  7.652(2)  6.578  Omnaris (TN), Alvesco (TN), Zetonna (TN), Ciclesonide (JAN/USAN/INN)
D01612  6.584(4)    -    Sodium picosulfate hydrate (JP17), Laxoberon (TN), Laxoberal (TN)
D00001  7.110(2)  6.585  Purified water in containers (JP17), Water, purified (USP), Water for injection (JP17), Sterile water (TN), Sterile purified water in containers (JP17), Water for injection in containers (JP17), Purified water (JP17), Water (JP17/USP)
D06249  7.532(2)  6.585  Water, tritiated (USAN), Tritiated water, Tritiotope (TN)
D06947  6.585(2)    -    Keishito
D02481  6.588(2)  7.551  Qing Hau Sau, Artemisinin (INN)
D03203  6.589(3)  8.509  Orencia (TN), Abatacept (genetical recombination) (JAN), Abatacept (USAN/INN)
D02486  7.056(2)  6.592  Nitazoxanide (USAN/INN), Alinia (TN)
D08681  7.620(2)  6.593  Von willebrand factor, Vonicog alfa (USAN), Wilfactin (TN), Vonicog alfa (genetical recombination) (JAN), Vonvendi (TN)
D02466  6.606(2)  8.107  Amodiaquine hydrochloride (USP), Camoquin hydrochloride (TN)
D02922  6.606(2)  8.107  Amodiaquine (USP/INN)
D10065  6.611(3)  7.568  Daclatasvir (USAN)
D10105  6.611(3)  7.568  Daclatasvir hydrochloride (JAN), Daclatasvir dihydrochloride (USAN), Daklinza (TN)
D10882  6.611(3)  7.568  Daclatasvir, asunaprevir and beclabuvir, Ximency (TN)
D00090  6.615(2)  6.910  Thrombin (JP17/USP/INN), Thrombostat (TN), Factor IIa
D08812  6.615(2)  6.910  Beriplast P combi-set (TN), Factor XIII with fibrinogen, Aprotinin, thrombin, human blood-coagulation factor XIII fraction, calcium chloride hydrate and freeze-dried human fibrinogen
D08813  6.615(2)  6.910  Tachocomb (TN), Aprotinin, thrombin and human fibrinogen
D07606  6.615(2)  6.778  Camostat (INN)
D04472  7.326(2)  6.617  Metharmon-F (TN), Pregnenolone, androstenedione, testosterone, estrone and dried thyroide
D06482  7.391(2)  6.617  Thyradin (TN), Thyroid (USP)
D09959  7.441(5)  6.622  Ruxolitinib (USAN/INN)
D00083  7.430(2)  6.626  Nitrogen (JP17/NF), Nitrogen (TN)
D01670  6.627(2)  7.336  Ronastat (TN), Nafamostat mesylate (USAN), Nafamostat mesilate (JP17)
D00751  6.628(3)  9.127  A-methapred (TN), Solu-medrol (TN), Methylprednisolone sodium succinate (JAN/USP)
D00473  7.008(2)  6.632  Rayos (TN), Meticorten (TN), Prednisone (USP), Prednisone monohydrate, Deltasone (TN)
D00836  8.493(2)  6.633  Buprenorphine hydrochloride (JP17/USP), Buprenex (TN)
D07132  8.493(2)  6.633  Buprenorphine (JAN/INN), Temgesic (TN)
D10250  8.493(2)  6.633  Bunavail (TN), Buprenorphine and naloxone, Suboxone (TN)
D03139  7.612(2)  6.651  Blood group specific substances A, B, and AB
D03365  7.736(2)  6.652  Habitrol (TN), Nicotine (USP)
D09196  6.655(2) 10.492  Candesartan cilexetil and hydrochlorothiazide (JP17), Ecard HD (TN), Ecard LD (TN)
D07057  6.662(2)  9.055  Abacavir (INN)
D00107  7.648(2)  6.671  Tacrolimus (USP/INN), Prograf (TN), Protopic (TN), Tacrolimus hydrate (JP17)
D08556  7.648(2)  6.671  Prograf (TN), Tacrolimus (INN)
D04070  6.680(4)  7.424  Premarin (TN), Estrogens, conjugated (JAN/USP), Conjugated estrogens
D04071  6.680(4)  7.424  Amnestrogen (TN), Estrogens, esterified (USP), Menest (TN)
D02300  6.681(4)  7.902  ATP (TN), Adenosine 5'-triphosphate disodium, Adenosine triphosphate disodium trihydrate, Adenosine triphosphate disodium hydrate (JAN)
D08646  6.681(4)  7.995  Triphosadenine (DCF), ATP
D00752  6.687(2)  6.882  Mycophenolate mofetil (JAN/USP), Cellcept (TN)
D05094  6.687(2)  6.882  Cellcept (TN), Mycophenolate mofetil hydrochloride (USAN)
D00413  6.692(2)  8.396  Retrovir (TN), Zidovudine (JP17/USP/INN)
D00979  6.693(3)    -    Methylprednisolone acetate (JAN/USP), Depo-medrol (TN)
D00126  7.725(2)  6.694  Advil (TN), Motrin (TN), Ibuprofen (JP17/USP/INN)
D02152  7.725(2)  6.694  Hydrocodone bitartrate and ibuprofen, Vicoprofen (TN)
D08059  7.725(2)  6.694  Ibuprofen sodium, Ibuprofen sodium anhydrous, Esprenit (TN)
D09760  7.725(2)  6.694  Ibuprofen sodium hydrate, Ibuprofen sodium (USAN)
D10449  7.725(2)  6.694  Ibuprofen and scopolamine butylbromide
D11573  7.725(2)  6.694  Oxycodone and ibuprofen
D11575  7.725(2)  6.694  Duexis (TN), Ibuprofen and famotidine
D11897  7.725(2)  6.694  Acetaminophen and ibuprofen
D04946  7.202(2)  6.700  Ferric pyrophosphate, soluble (JAN), Incremin (TN)
D07056  6.703(3)    -    Rokumigan, Rokumijiogan
D06970  6.703(3)    -    Jiinkokato
D00902  6.709(2)  8.637  Relenza (TN), Zanamivir (USP/INN)
D01937  6.709(2)  8.637  Relenza (TN), Zanamivir hydrate (JAN)
D10639  6.710(3)  8.042  Grazoprevir hydrate (JAN), Grazoprevir (USAN), Grazyna (TN)
D10778  6.710(3)  8.042  Elbasvir and grazoprevir, Zepatier (TN)
D11565  6.710(3)  8.042  Grazoprevir (USAN/INN), Grazoprevir anhydrous
D00400  6.722(2)  8.672  Diovan (TN), Valsartan (JP17/USP/INN)
D09197  6.722(2)  8.672  Co-dio (TN), Valsartan and hydrochlorothiazide (JP17)
D09745  6.722(2)  8.672  Valsartan and amlodipine besilate, Exforge (TN)
D10286  6.722(2)  8.258  Amlodipine, valsartan and hydrochlorothiazide, Exforge hct (TN)
D10287  6.722(2)  8.672  Valturna (TN), Aliskiren hemifumarate and valsartan
D10525  6.722(2)  8.672  Atedio (TN), Valsartan and cilnidipine
D11388  6.722(2)  8.672  Nebivolol and valsartan, Byvalson (TN)
D02126  6.726(2)  9.342  Primaquine phosphate (JAN/USP), Primaquine (TN)
D08420  6.726(2)  9.342  Primaquine (INN), Kanaprim (TN)
D10827  6.732(3)  6.759  Epclusa (TN), Sofosbuvir and velpatasvir
D10900  6.732(3)  6.759  Vosevi (TN), Sofosbuvir, velpatasvir and voxilaprevir
D00056  6.732(2)  7.250  Parenzyme (TN), Trypsin, crystallized (USP), Trypsin (JAN)
D04756  6.732(2)  7.250  Francetin T (TN), Fradiomycin sulfate and trypsin, crystallized
D08753  6.732(2)  7.250  Bromelains and trypsin, crystallized, Kimotab (TN)
D02141  7.643(2)  6.736  Iron dextran (USP), Infed (TN)
D08170  7.448(2)  6.745  Melatonin (JAN), Melatonina (TN), Melatobel (TN)
D00007  6.756(2) 10.448  Glutamic acid (USP), L-Glutamic acid (JP17)
D04341  6.756(2) 10.688  DL-Glutamic acid, Glutamic acid (USAN)
D07539  6.756(2) 10.688  Glutamic acid hydrochloride, Hypochylin (TN)
D03304  7.069(2)  6.757  Interferon beta (JAN), Feron (TN)
D00076  6.758(2)  8.210  Nor adrenalin (TN), Norepinephrine (INN), Noradrenaline (JP17)
D09794  6.758(2)  8.210  Norepinephrine hydrochloride (JAN), (+/-)-Noradrenaline hydrochloride
D00109  7.053(2)  6.762  Aspalon (JAN), Durlaza (TN), Acetylsalicylic acid, Easprin (TN), Aspirin (JP17/USP)
D02079  7.053(2)  6.762  Codein phosphate and aspirin, Empirin compound (TN)
D02154  7.053(2)  6.762  Codoxy (TN), Percodan-demi (TN), Aspirin, oxycodone hydrochloride and oxycodone terephthalate
D02155  7.053(2)  6.762  Darvon compound-65 (TN), Aspirin, caffeine and propoxyphene hydrochloride
D03960  7.053(2)  6.762  Aspirin and dialuminate, Bufferin (TN), Aspirin, aluminum glycinate and magnesium carbonate
D03969  7.053(2)  6.762  Aspirin and ascorbic acid, E.A.C (TN)
D07582  7.053(2)  6.762  Catalgine (TN), Sodium acetylsalicylate, Aspirin sodium
D10513  7.053(2)  6.762  Clopidogrel and acetylsalicyclic acid, Clopidogrel sulfate and aspirin, Complavin (TN)
D11176  7.053(2)  6.762  Oxycodone hydrochloride and aspirin, Percodan (TN)
D11586  7.053(2)  6.762  Carisoprodol and aspirin
D11587  7.053(2)  6.762  Carisoprodol, aspirin and codeine phosphate
D11615  7.053(2)  6.762  Aggrenox (TN), Aspirin and dipyridamole
D11616  7.053(2)  6.762  Aspirin and omeprazole, Yosprala (TN)
D11804  7.053(2)  6.762  Aspirin and vonoprazan, Cabpirin (TN)
D11849  7.053(2)  6.762  Orphenadrine citrate, aspirin and caffeine, Norgesic forte (TN)
D11859  7.053(2)  6.762  Butalbital, aspirin and caffeine, Fiorinal (TN), Lanorinal (TN)
D11860  7.053(2)  6.762  Butalbital, aspirin, caffeine and codeine, Fiorinal with codeine (TN)
D00008  7.503(2)  6.764  Oxydol (JP17), Hydrogen peroxide (USP), Oxyfull (TN)
D00108  7.399(2)  6.770  Iodine (JP17/USP), Cadex (TN)
D03283  7.399(2)  6.770  Iodine and cadexomer, Iodosorb (TN), Cadexomer iodine (USAN)
D04838  7.399(2)  6.770  Zinc iodide and iodine, Iodine zinc iodide for Kantop (TN)
D04875  7.399(2)  6.770  Zinc sulfate, iodine, sodium iodine and glycerin, Neoglycerol (TN)
D07709  7.399(2)  6.770  Polyvinyl alcohol and iodine, PA iodo (TN)
D03597  7.110(2)  6.775  Cotton, purified (USP)
D00924  6.778(2)  7.164  Ceftriaxone sodium (USP), Rocephin (TN), Ceftriaxone sodium hydrate (JP17)
D07659  6.778(2)  7.164  Ceftriaxone (TN), Ceftriaxone (INN)
D06286  7.158(2)  6.780  Vegetable oil, hydrogenated (NF), Hydrogenated vegetable oil
D09004  7.158(2)  6.780  Soybean oil, hydrogenated (NF)
D00701  6.783(3)  7.801  Luminal sodium (TN), Phenobarbital sodium (JAN/USP/INN)
D06199  6.789(2)    -    Trabectedin (JAN/USAN/INN), Yondelis (TN)
D03923  8.006(2)  6.789  Dusting powder, absorbable (USP)
D04305  8.006(2)  6.789  Gauze, absorbent
D06428  8.006(2)  6.789  Gelatin film, absorbable, Gelfilm (TN)
D06429  8.006(2)  6.789  Gelfoam (TN), Gelatin sponge, absorbable
D10779  6.798(3)  9.488  Avycaz (TN), Ceftazidime and avibactam, Zavicefta (TN)
D00472  7.520(2)  6.802  Delta-cortef (TN), Prednisolone (JP17/USP/INN)
D04251  7.520(2)  6.802  Cor tyzine (TN), Tetrahydrozoline hydrochloride and prednisolone
D04753  7.520(2)  6.802  Chlomy-P (TN), Chloramphenicol, fradiomycin sulfate and prednisolone
D04769  7.520(2)  6.802  Aersolin D (TN), Fradiomycin sulfate and prednisolone
D11699  7.520(2)  6.802  Sulfacetamide sodium and prednisolone
D02577  6.804(3)    -    Addyi (TN), Flibanserin (USAN/INN)
D10590  6.806(3) 10.463  Trinessa (TN), Norgestimate and ethinyl estradiol, Mononessa (TN)
D10839  6.806(3) 10.463  Evra (TN), Norelgestromin and ethinyl estradiol, Xulane (TN)
D07043  6.808(2)    -    Maobushisaishinto
D10625  6.810(3)  8.446  Elbasvir (JAN/USAN/INN), Erelsa (TN)
D10223  6.814(5)  7.799  Imbruvica (TN), Ibrutinib (JAN/USAN)
D00040  6.815(2)  7.275  Cholesterol (JP17/NF), Cholesterol (TN)
D02326  6.818(2)  6.933  L-Cysteine hydrochloride hydrate (JP17), Elcys (TN), Cysteine hydrochloride (USP)
D08748  6.818(2)  6.933  Liver hydrolysate, cysteine hydrochloride, choline bitartrate, inositol and cyanocobalamin, Proheparum (TN)
D02490  7.052(2)  6.824  Tylan (TN), Tylosin (USP/INN), TS
D00445  6.828(2)  9.752  Zerit (TN), Sanilvudine (JAN), Stavudine (USAN/INN)
D00848  6.829(3)    -    Benylin DM (TN), Dextromethorphan hydrobromide (USP), Dextromethorphan hydrobromide hydrate (JP17)
D11932  6.832(3)    -    Duobrii (TN), Halobetasol and tazarotene, Ulobetsol and tazarotene
D00167  7.124(2)  6.851  BAL (TN), Dimercaprol (JP17/USP/INN)
D02194  7.687(2)  6.853  Cilastatin sodium (JP17/USP), CS
D08066  6.857(4)  7.939  Glamox (TN), Imatinib (INN)
D00082  6.860(2)    -    Pyridoxamine phosphate dihydrate, Pyridoxamine phosphate (JAN)
D09002  6.869(2)    -    Dried sodium sulfite (JP17), Sodium sulfite (NF)
D00013  6.878(3)  8.995  L-Aspartic acid (JP17), Aspartic acid (USP/INN)
D05987  6.888(4)    -    Enjuvia (TN), Synthetic conjugated estrogens, B (USAN)
D10428  6.888(3)  8.473  Tenofovir alafenamide (USAN/INN)
D03257  6.891(2)  9.442  Herzuma (TN), Trastuzumab (genetical recombination) [Trastuzumab biosimilar 2] (JAN), Kanjinti (TN), Trastuzumab-anns, Trastuzumab (genetical recombination) [Trastuzumab biosimilar 3] (JAN), Trastuzumab-qyyp, Trastuzumab (genetical recombination) (JAN), Trastuzumab-dttb, Trastuzumab (genetical recombination) [Trastuzumab biosimilar 1] (JAN), Trastuzumab-dkst, Trastuzumab (USAN/INN), Ogivri (TN), Herceptin (TN), Trastuzumab-pkrb
D11560  6.891(2)  9.442  Herceptin hylecta (TN), Trastuzumab and hyaluronidase, Trastuzumab and hyaluronidase-oysk
D11934  6.891(2)  9.442  Phesgo (TN), Pertuzumab, trastuzumab and hyaluronidase, Pertuzumab, trastuzumab and hyaluronidase-zzxf
D09731  6.908(2) 10.441  Xalkori (TN), Crizotinib (JAN/USAN/INN)
D09025  6.909(2)  7.700  Triglycerides, medium-chain (NF)
D02370  6.912(3)  7.813  CVA, Clavulanate potassium (JP17/USP)
D06886  6.913(3)  7.446  Tysabri (TN), Natalizumab (genetical recombination) (JAN), Natalizumab (USAN/INN)
D00433  8.269(3)  6.918  Sulfadiazine silver (JP17), Silvadene (TN), Sulfadiazine, silver (USP)
D01211  6.919(5) 10.495  Calcium folinate (JP17), Leucovorin calcium (USP), Wellcovorin (TN), Uzel (TN)
D07986  6.919(5) 10.495  Rescuvolin (TN), Leucovorin, Folinic acid (BAN)
D07987  6.919(5) 10.495  Leucovorin calcium pentahydrate, Leucovorin (TN), Folinic acid calcium salt pentahydrate
D02837  8.627(4)  6.920  Alteplase (USP/INN), Alteplase (genetical recombination) (JAN), Cathflo activase (TN), Activase (TN)
D03843  6.921(2)  9.144  Tipranavir disodium (USAN)
D08605  6.921(2)  9.144  Tipranavir (INN), Aptivus (TN)
D11857  6.924(3)  9.139  Duaklir pressair (TN), Aclidinium bromide and formoterol fumarate
D08787  7.432(2)  6.924  Freeze-dried pepsin treated human normal immunoglobulin, Globulin (TN)
D04011  6.927(2)  9.514  Kashowadol (TN), Chondroitin sulfate sodium and sodium salicylate
D04078  6.927(2)  9.514  Chondron (TN), Chondroitin sulfate sodium (JAN)
D04084  6.927(2)  9.514  Chondron napha (TN), Chondroitin sulfate sodium and naphazoline hydrochloride
D04945  6.927(2)  9.514  Chondroitin sulfate and iron colloid, Blutal (TN)
D07632  6.927(2)  9.073  Chondroitin sulfate sodium and sodium hyaluronate, Viscoat (TN)
D07633  6.927(2)  9.491  Mucotear (TN), Chondroitin sulfate sodium and FAD sodium, Mucofadin (TN)
D02367  6.934(3)    -    Desogestrel (USAN/INN)
D09971  6.935(3)  8.432  Tegobuvir (USAN/INN)
D07086  6.938(2)  7.536  Xarelto (TN), Rivaroxaban (JAN/USAN/INN)
D02125  7.564(2)  6.941  Chloroquine phosphate (USP), Aralen (TN)
D00874  7.352(2)  6.944  Vigamox (TN), Moxifloxacin hydrochloride (JAN/USP), Avelox (TN)
D08237  7.352(2)  6.944  Avelox IV (TN), Moxifloxacin (INN)
D11618  7.352(2)  6.944  Moxifloxacin hydrochloride monohydrate
D00750  6.945(5)  9.306  Ergamisol (TN), Levamisole hydrochloride (USP)
D01264  6.945(5)  9.362  Daunorubicin hydrochloride (JP17/USP), Cerubidine (TN)
D05401  7.384(2)  6.948  Kogenate FS (TN), Octocog alfa (INN), Octocog alfa (genetical recombination) (JAN), Factor VIII (rDNA)
D00307  6.962(2)  6.948  Doxycycline (USP), Doxycycline hydrate, Monodox (TN), Vibramycin (TN), Oracea (TN)
D02129  6.962(2)  6.948  Vibra-tabs (TN), Doxycycline hyclate (USP), Periostat (TN), Doxycycline hydrochloride hydrate (JP17), Doryx (TN), Lymepak (TN)
D03903  6.962(2)  6.948  Doxycycline calcium (USP)
D07876  6.962(2)  6.948  Doxycycline (TN), Doxychel (TN), Doxycycline (INN)
D07877  6.962(2)  6.948  Vibramycin (TN), Doxycycline hydrochloride
D00074  6.962(2)  7.089  Nitric oxide (JAN/USAN), INOmax (TN)
D01136  7.502(2)  6.964  Rikavarin (TN), Transamin (TN), Tranexamic acid (JP17/USP/INN), Cyklokapron (TN)
D00157  6.966(2)  7.012  Glycyrrhizic acid, Glycyrrhizin (JAN)
D00190  7.426(2)  6.970  Rutin hydrate (JAN), Rutin trihydrate
D04675  7.426(2)  6.970  Melilot extract and rutin hydrate, Esberiven (TN)
D08499  7.426(2)  6.970  Rutin, Venoruton (TN), Rutoside (INN)
D04518  6.977(3)  8.240  Inalimarev (CEA, MUC-1, vaccinia virus) (USAN), Panvac (TN), PANVAC-V
D02747  6.980(3)    -    Peginterferon alfa-2a (USAN/INN), Peginterferon alfa-2a (genetical recombination) (JAN), Pegasys (TN)
D06457  6.980(3)  8.445  Gonadotropin, chorionic (USP), Human chorionic gonadotrophin (JP17), A.P.L. (TN), HCG (TN)
D06440  7.724(2)  6.983  Ether, anesthetic (JP17)
D09881  6.985(4)  7.241  Cobicistat (JAN/USAN/INN), Tybost (TN)
D02102  8.141(2)  6.986  Methadone hydrochloride (JAN/USP), Dolophine hydrochloride (TN)
D08195  8.141(2)  6.986  Methadone (BAN)
D01446  7.285(2)  6.990  Magnesium carbonate (TN), Magnesium carbonate (JP17/USP)
D01780  7.285(2)  6.990  Sodium carbonate hydrate (JP17)
D02038  7.285(2)  6.990  Potassium carbonate (JP17/USP), Racol (TN)
D07631  7.285(2)  6.990  Sulcain (TN), Ethyl piperidinoacetylaminobenzoate, magnesia alumina hydrate and precipitated calcium carbonate
D07703  7.285(2)  6.990  Hydroxyethyl cellulose, boric acid, dibasic sodium phosphate, potassium chloride, sodium chloride and dried sodium carbonate, Scopisol (TN)
D08704  7.285(2)  6.990  Magnesium aluminometasilicate, methylmethionine sulfonium chloride, magnesium carbonate and precipitated calcium carbonate, Cabagin-U (TN)
D10259  7.285(2)  6.990  Pepcid complete (TN), Famotidine, calcium carbonate and magnesium hydroxide
D10802  7.229(2)  6.990  Calcium carbonate (13C) (JAN), Carbonic-13C acid, calcium salt
D07053  6.991(3)    -    Ryutanshakanto
D06915  6.991(3)    -    Anchusan
D06931  6.991(3)    -    Kambakutaisoto
D06997  6.991(3)    -    Seishoekkito
D01108  6.995(2)  7.186  Magnesium sulfate heptahydrate, Magnesium sulfate (USP), Magnesium sulfate hydrate (JP17), Conclyte-Mg (TN)
D01726  6.995(2)  7.186  Potassium sulfate (JP17/USAN)
D05877  6.995(2)  7.186  Sodium sulfate decahydrate, Natrium Sulfuricum, Natrii Sulfus, Sodium sulfate (TN), Sodium sulfate hydrate (JP17), Sodium sulfate (USP)
D05963  6.995(2)  7.186  Sulfate, Sulfuric acid (NF)
D09201  6.995(2)  7.186  Calcium sulfate (NF)
D11552  6.995(2)  7.186  Moviprep (TN), Polyethylene Glycol 3350, sodium sulfate, sodium chloride, potassium chloride, sodium ascorbate and ascorbic acid, Plenvu (TN)
D11985  6.995(2)  7.186  Sulprep (TN), Magnesium sulfate, anhydous sodium sulfate and potassium sulfate
D04409  6.995(4)    -    Halobetasol propionate (USP), Ultravate (TN), Ulobetasol propionate
D09706  7.614(2)  6.998  Carbon monoxide (USAN)
D01710  7.000(2)    -    Rulide (TN), Roxithromycin (JP17/USAN/INN)
D00025  7.110(2)  7.006  Sugar, compressible (NF), Sugar, confectioner's (NF), Sucrose (TN), Sucrose, purified, White soft sugar (JP17), Sucrose (JP17/NF), Sugar spheres (NF)
D01144  7.577(3)  7.008  Sword (TN), Prulifloxacin (JAN/INN)
D02867  7.011(3)  9.136  Lexiva (TN), Fosamprenavir calcium hydrate (JAN)
D03835  7.011(3)  9.136  Lexiva (TN), Fosamprenavir calcium (USAN), Telzir (TN)
D08043  7.014(3)  9.036  Captique (TN), Hyaluronic acid (BAN), Monovisc (TN)
D00211  7.021(4)  9.218  Rimactane (TN), Rifampin (USP), Rifampicin (JP17/INN), Rifadin (TN)
D10210  7.021(4)  9.363  Rifater (TN), Rifampin, isoniazid and pyrazinamide
D11578  7.021(4)  9.882  Rifampin and isoniazid
D00952  9.273(2)  7.022  Megestrol acetate (USP), Megace (TN)
D09707  7.024(2)  8.863  Dabigatran (USAN/INN)
D09996  7.028(4) 10.436  Vemurafenib (JAN/USAN/INN), Zelboraf (TN)
D07769  7.033(3)  8.732  Cytidine, Posilent (TN)
D00496  7.036(2) 10.489  Penicillamine (JAN/USP/INN), Depen (TN), Cuprimine (TN)
D08330  7.036(2) 10.489  Pemine (TN), Penicillamine hydrochloride
D00627  7.050(2)  8.652  Micardis (TN), Telmisartan (JP17/USP/INN)
D09219  7.050(2)  8.652  Telmisartan and hydrochlorothiazide (JP17), Micombi (TN), Micardis hct (TN)
D09743  7.050(2)  8.652  Telmisartan and amlodipine besilate, Micamlo (TN), Telmisartan and amlodipine besylate, Twynsta (TN)
D10805  7.050(2)  8.258  Micatrio (TN), Telmisartan, amlodipine and hydrochlorothiazide
D10837  7.052(2) 10.453  Phenylephrine and ketorolac, Omidria (TN)
D11853  7.052(2)  9.217  Phenylephrine and promethazine
D00511  7.052(2)    -    Phenylephrine hydrochloride (JP17/USP), Neo-synephrine (TN)
D04081  7.052(2)    -    Tropicamide and phenylephrine hydrochloride, Sandol P (TN)
D08365  7.052(2)    -    Phenylephrine Minims (TN), Phenylephrine (INN)
D11698  7.052(2)    -    Cyclopentolate hydrochloride and phenylephrine hydrochloride, Cyclomydril (TN)
D00217  8.018(2)  7.059  Tylenol (TN), Paracetamol (INN), Acetaminophen (JP17/USP)
D00846  8.018(2)  7.059  Vicodin (TN), Hydrocodone bitartrate and paracetamol, Hydrocodone bitartrate and acetaminophen
D00186  7.059(2)  8.418  Otiprio (TN), Cipro (TN), Ciprofloxacin (JP17/USP/INN)
D02216  7.059(2)  8.418  Ciprofloxacin hydrochloride hydrate (JP17), Cipro (TN), Ciprofloxacin hydrochloride (USP), Ciloxan (TN), Proquin XR (TN)
D10822  7.059(2)  8.418  Ciprofloxacin hydrate (JAN), Ciprofloxacin hemiheptahydrate
D11089  7.059(2)  8.418  Otovel (TN), Ciprofloxacin hydrochloride and fluocinolone acetonide
D11582  7.059(2)  7.179  Ciprofloxacin hydrochloride and hydrocortisone, Cipro hc (TN)
D00004  7.640(2)  7.059  Carbon dioxide (JP17/USP), Carbon dioxide (TN)
D00588  7.068(2)  7.848  Levofloxacin (USP), Iquix (TN), Levaquin (TN), Levofloxacin hydrate (JP17), Levofloxacin hemihydrate, Quixin (TN)
D08120  7.068(2)  7.848  Levofloxacin (INN), Cravit (TN), Levaquin (TN)
D10527  7.082(3)  8.217  Takelda (TN), Acetylsalicylic acid and lansoprazole
D00408  8.890(3)  7.084  Android (TN), Testred (TN), Methyltestosterone (JP17/USP/INN)
D04968  7.577(2)  7.088  Yellow beewax (JP17), Yellow wax (TN), Wax, Yellow (NF)
D05240  7.577(2)  7.088  Ointment, yellow (USP)
D06516  7.577(2)  7.088  Phenolphthalein, yellow, Feen-a-mint gum (TN)
D03354  7.100(4)  9.040  Sodium hyaluronate (JAN), Purified sodium hyaluronate (JP17), Legend (TN), Hyaluronate sodium (USAN)
D06579  7.101(5)    -    Denibulin hydrochloride (USAN)
D00015  7.106(3)  8.388  Nutrestore (TN), L-Glutamine (JP17), Glutamine (USP), Levoglutamide, Endari (TN)
D00229  7.108(2)  7.834  Amoxicilline (INN), Amoxicillin hydrate (JP17), Amoxicillin trihydrate, Dispermox (TN), Amoxicillin (USP), Amoxil (TN), Pasetocin (TN)
D00230  7.108(2)  7.834  Amoxicillin and clavulanate potassium, Amoxicillin and potassium clavulanate
D02925  7.108(2)  7.834  Amoxicillin sodium (USAN)
D06485  7.108(2)  7.834  Augmentin (TN), Amoxicillin hydrate and potassium clavulanate
D07452  7.108(2)  7.834  Amoxicillin (TN), AMPC, Amoxicillin (INN)
D09742  7.108(2)  7.834  Lansoprazole, amoxicillin hydrate and metronidazole, Lampion (TN)
D10520  7.108(2)  7.834  Rabeprazole, amoxicillin and metronidazole, Rabefine (TN)
D10774  7.108(2)  7.834  Vonopion (TN), Vonoprazan, amoxicillin and metronidazole
D11858  7.108(2)  7.834  Talicia (TN), Omeprazole magnesium, amoxicillin and rifabutin
D05707  7.110(2)  7.195  Rayon, Rayon, purified (USP)
D05839  7.110(2)  7.195  Siliceous earth, purified (NF)
D06459  7.110(2)  7.195  Purified human menopausal gonadotrophin (JAN), Human menopausal gonadotrophin, purified
D06776  7.110(2)  7.195  Honey (JP17), Honey (TN), Honey, purified (NF)
D02811  7.111(4)    -    Alicaforsen sodium (USAN)
D00895  7.111(4)    -    Delavirdine mesilate (JAN), Rescriptor (TN), Delavirdine mesylate (USAN)
D00863  7.113(2)  7.185  Betadine (TN), Povidone-iodine (JP17/USP)
D00563  7.644(2)  7.116  Reflex (TN), Remeron (TN), Mirtazapine (JAN/USP/INN)
D07747  7.175(4)  7.118  Rowatin (TN), alpha, beta-Pinene, borneol, anetholtrithion, d-camphene, cineole and fenchone
D08479  7.120(4)  9.218  Rifadine (TN), Rifampicin sodium
D00296  7.121(2) 10.438  Didanosine (JAN/USP/INN), Videx (TN)
D09385  7.123(2)    -    Rambazole (TN), Talarozole (USAN/INN)
D10288  7.128(3)  9.724  Olmesartan medoxomil, amlodipine besylate and hydrochlorothiazide, Tribenzor (TN)
D01724  7.130(2)  7.293  Aluminum potassium sulfate hydrate (JP17), Alum, potassium (USP), Aluminum potassium sulfate (TN)
D08403  7.130(2)  7.293  Nabumeton A (TN), Potassium
D00592  7.131(2)  9.077  Aczone (TN), Dapsone (USP), Diaphenylsulfone (JAN)
D06532  8.258(2)  7.132  Travert 10% in plastic container (TN), Sugar, invert (USP)
D01790  7.132(5) 10.559  Oxaliplatin (JAN/USP/INN), Eloxatin (TN)
D00523  7.135(2)  9.743  Avapro (TN), Irbesartan (JP17/USP/INN)
D10243  7.135(2)  9.743  Aimix (TN), Irbesartan and amlodipine besilate (JP17)
D10249  7.135(2)  9.743  Avalide (TN), Ifirmacombi (TN), Irbesartan and hydrochlorothiazide
D10480  7.135(2)  9.743  Irtra (TN), Irbesartan and trichlormethiazide
D05527  8.547(2)  7.136  Platelet concentrate (USP), PC
D06571  7.139(2)  8.351  Antithrombin III (INN), Antithrombin III human (USP), Kybernin (TN)
D02248  7.140(3)    -    Levomepromazine maleate (JP17/USAN), Hirnamin (TN)
D03976  8.194(2)  7.141  Isopropylantipyrine, allyl isopropyl acetyl urea, acetaminophen and anhydrous caffeine, SG (TN)
D09546  7.154(3)    -    Savaysa (TN), Edoxaban tosylate monohydrate, Lixiana (TN), Edoxaban tosilate hydrate (JAN)
D01322  7.155(3)    -    TAO, Triacetyloleandomycin (JAN), Troleandomycin (USAN/INN), Tao (TN)
D04471  8.660(2)  7.157  Ethinylestradiol and methylestrenolone, EP (TN)
D08660  7.158(5)    -    Ulobetasol (INN), Halobetasol
D02482  7.159(2)  8.658  Artesunate (TN), Artesunate (USAN), Artesunic acid, Arsumax (TN)
D08260  7.168(3)    -    Neomycin (INN), Fradiomycin, Neomycin, Kaomycine (TN)
D00002  7.173(4)  9.125  Nadide (JAN/USAN/INN), Nicotinamide adenine dinucleotide
D00045  7.174(2)  7.346  Adenoscan (TN), Adenosine (JAN/USP), Adenocard (TN)
D01766  7.174(3)  7.334  Foipan (TN), Camostat monomethanesulfonate, Camostat mesylate, Camostat mesilate (JP17)
D10093  7.175(3) 10.418  Sunvepra (TN), Asunaprevir (JAN/USAN)
D00088  7.628(3)  7.179  Anusol HC (TN), Cortef (TN), Colocort (TN), Hydrocortisone (JP17/USP/INN), Acticort (TN), Plenadren (TN), Hytone (TN), HC
D04699  7.714(2)  7.179  Proctosedyl (TN), Hydrocortisone, fradiomycin sulfate, dibucaine hydrochloride and esculoside
D04776  7.714(2)  7.179  Terra Cortril (TN), Oxytetracycline hydrochloride and hydrocortisone
D04801  7.714(2)  7.179  Hydrocortisone and crotamiton, Eurax H (TN)
D08827  7.714(2)  7.179  Posterisan forte (TN), Hydrocortisone and killed escherichia coli suspension
D11855  7.714(2)  7.179  Neomycin, polymyxin B sulfate and hydrocortisone, Cortisporin (TN), Casporyn hc (TN)
D07423  7.184(3)  9.147  Defibrotide sodium (JAN/USAN), Defitelio (TN)
D02503  7.184(4)  9.385  Augpenin (TN), Timentin (TN), Clavulanic acid and ticarcillin
D10605  7.185(3)    -    Tenofovir alafenamide fumarate (USAN/JAN), Vemlidy (TN)
D09669  7.865(2)  7.186  Siltuximab (USAN/INN), Sylvant (TN)
D04112  7.188(2) 10.421  Intelence (TN), Etravirine (JAN/USAN/INN)
D01655  7.190(4)    -    Adefovir pivoxil (JAN), Hepsera (TN), Adefovir dipivoxil (USAN)
D00258  7.193(2)  9.205  Cefixime (INN)
D07640  7.193(2)  9.205  Cefixime hydrate (JP17), Cefixime trihydrate, Oroken (TN), Cefixime (USP), Suprax (TN)
D02146  8.443(2)  7.201  Acetaminophen and codeine phosphate, Tylenol w/codeine (TN)
D02153  8.443(2)  7.201  Acetaminophen and oxycodone hydrochloride, Tylox (TN), Percocet (TN), Roxicet 5/500 (TN)
D04044  8.443(2)  7.201  LL (TN), Chlorpheniramine maleate, acetaminophen, salicylamide and anhydrous caffeine
D04046  7.592(2)  7.201  Salicylamide, acetaminophen, anhydrous caffeine and promethazine methylenedisalicylate, PL (TN)
D08695  7.928(3)  7.201  dl-Methylephedrine hydrochloride, dihydrocodeine phosphate, diprophylline, diphenhydramine salicylate, acetaminophen and bromovalerylurea, Coughcode N (TN)
D09999  8.443(2)  7.201  Tramcet (TN), Tramadol hydrochloride and acetaminophen
D11561  8.443(2)  7.201  Allzital (TN), Tencon (TN), Bupap (TN), Butapap (TN), Butalbital and acetaminophen
D11628  8.443(2)  7.201  Benzhydrocodone and acetaminophen, Apadaz (TN)
D11850  8.443(2)  7.201  Butalbital, acetaminophen and caffeine
D11851  8.443(2)  7.201  Butalbital, acetaminophen, caffeine and codeine phosphate
D03917  7.203(3)    -    Drospirenone (JAN/USP/INN), Slynd (TN)
D01825  7.204(4)    -    Fluocinolone acetonide (JP17/USP/INN), Retisert (TN), Synalar (TN), Fluocet (TN)
D04795  7.204(4)    -    Flucort F (TN), Neomycin sulfate and fluocinolone acetonide, Fradiomycin sulfate and fluocinolone acetonide, Neo-synalar (TN)
D00054  7.206(2)  8.904  Inosine (JAN/INN), Inotin (TN)
D10806  7.210(3)  8.448  Velpatasvir (JAN/USAN/INN)
D10066  7.212(4)  8.484  Dolutegravir (USAN), Tivicay (TN)
D10113  7.212(4)  8.484  Dolutegravir sodium (JAN/USAN), Tivicay (TN)
D11282  7.212(4)  8.484  Juluca (TN), Dolutegravir sodium and rilpivirine hydrochloride
D01126  7.214(2)  8.333  R-gene (TN), Arginine hydrochloride (USP), L-Arginine hydrochloride (JP17)
D02982  7.214(2)  8.333  Arginine (USP/INN), L-Arginine (JP17)
D06483  7.214(2)  8.333  R-gene (TN), Arginine hydrochloride
D04187  7.935(2)  7.219  Gilenya (TN), Fingolimod hydrochloride (JAN/USAN)
D10001  7.935(2)  7.219  Gilenya (TN), Fingolimod (INN)
D06500  8.892(2)  7.226  Dried bitter orange peel (TN), Orange peel tincture (JP17), Orange peel tincture, sweet (NF), Osbeck (TN)
D04519  7.704(2)  7.228  Incyclinide (USAN), Metastat (TN)
D05794  7.229(2)  7.611  Salts, rehydration (USP)
D07554  7.229(2)  7.404  L-Cysteine, N-acetyl-, sodium salt, Acetylcysteine Sodium (TN), Acetylcysteine sodium salt
D05857  7.234(2)    -    Sodium dichloroacetate (USAN), Ceresine (TN)
D01953  7.236(3)  9.763  Ovahormon (TN), Estradiol benzoate (JP17/USP)
D04465  7.236(3)  9.763  Hydroxyprogesterone caproate and estradiol benzoate, Lutes (TN)
D04800  7.236(3)  8.465  Amel S (TN), Heparinoid, adrenal extract and salicylic acid
D08740  7.236(3)    -    Bone wax (TN), Yellow beewax, almond oil and salicylic acid
D00097  7.236(3)    -    Salicylic acid (JP17/USP), Salicylic acid (TN)
D00036  7.240(4)  8.449  Niacinamide (USP), Nicotinamide (JP17/INN)
D04252  7.240(4)  8.449  Nicotinamide and papaverine hydrochloride, Stomin A (TN)
D04915  7.240(4)  8.386  Calcium pantothenate, riboflavin, pyridoxine hydrochloride and nicotinamide, Pancal (TN)
D07851  7.240(4)  7.842  Ascorbic acid, thiamine chloride hydrochloride, pyridoxine hydrochloride, riboflavin sodium phosphate, nicotinamide and panthenol, C para (TN)
D07859  7.240(4)  7.842  Ascorbic acid, thiamine nitrate, nicotinamide, calcium pantothenate, pyridoxine hydrochloride and riboflavin, Wasser-V (TN)
D08830  7.240(4)  7.842  Retinol palmitate, thiamine nitrate, riboflavin, pyridoxine hydrochloride, cyanocobalamin, ascorbic acid, ergocalciferol, tocopherol acetate, calcium pantothenate, nicotinamide and folic acid, Panvitan (TN)
D10085  7.240(4)    -    Alisertib (USAN)
D10086  7.240(4)    -    Alisertib sodium (USAN), Alisertib sodium hydrate
D02497  7.258(2)  9.136  Fosamprenavir (INN)
D03837  7.258(2)  9.136  Fosamprenavir sodium (USAN)
D01697  7.259(4)    -    Colforsin daropate hydrochloride (JAN), Nkh 477, Adehl (TN)
D00362  7.262(2)  8.553  Lisinopril (USP), Zestril (TN), Lisinopril hydrate (JP17), Prinivil (TN), Lisinopril dihydrate
D08131  7.262(2)  8.553  Zestril (TN), Lisinopril (INN)
D10268  7.262(2)  8.553  Hydrochlorothiazide and lisinopril, Zestoretic (TN), Prinzide (TN)
D00318  8.161(2)  7.262  Fluxid (TN), Famotidine (JP17/USP/INN), Pepcid (TN)
D02748  7.262(3)    -    Pegintron (TN), Peginterferon alfa-2b (genetical recombination) (JAN), Peginterferon alfa-2b (USAN/INN), Sylatron (TN)
D10557  7.268(4)    -    Repatha (TN), Evolocumab (USAN/INN), Evolocumab (genetical recombination) (JAN)
D02293  7.270(3)  7.861  Calcium ascorbate (USP)
D05853  7.270(3)  7.861  Sodium ascorbate (USP/INN), Cevalin (TN)
D07575  7.270(3)  7.861  Ascorbic acid calcium salt, Calcium Ascorbate (TN)
D03213  7.274(2)  7.670  Apixaban (JAN/USAN/INN), Eliquis (TN)
D04316  7.279(3)    -    Gestodene (USAN/INN)
D03343  7.279(2)  7.663  Dextran sulfate sodium sulfur 5 (JP17), Dextran sulfate sodium sulfur 18 (JP17), MDS (TN)
D04365  7.759(2)  7.280  Glycyrrhizae radix praeparata, Liquorice, Licorice (NF), Powdered glycyrrhiza (JP17), Prepared glycyrrhiza (JP17), Glycyrrhiza (JP17), Glycyrrhizae radix, Glycyrrhiza (TN)
D08710  7.759(2)  7.280  Compound glycyrrhiza (TN), Sulfur, fennel, glycyrrhiza and senna leaf
D02842  7.281(2)  7.908  Alum, ammonium (USP)
D05531  7.281(2)  7.908  Rhus tox antigen (TN), Poison ivy extract, alum precipitated (USAN), Alum precipitated poison ivy extract
D00742  7.284(2)  8.304  Etanercept (USAN/INN), Enbrel (TN), Etanercept (genetical recombination) (JAN), Etanercept (genetical recombination) [Etanercept biosimilar 2] (JAN), Etanercept (genetical recombination) [Etanercept biosimilar 1] (JAN)
D04744  7.797(2)  7.289  Sodium thiosulfate and ethanol, Hypoethanol (TN)
D04761  7.797(2)  7.289  Camphor (TN), d-Camphor and ethanol
D06542  7.797(2)  7.289  Ethanol (TN), Ethanol (JP17)
D06620  7.797(2)  7.289  Camphor (TN), dl-Camphor and ethanol
D07913  7.291(2)  8.553  Escitalopram (INN), Esertia (TN)
D10335  7.293(4)    -    Alirocumab (USAN), Praluent (TN)
D10657  7.294(3)    -    Pradigastat sodium (USAN)
D10664  7.294(3)    -    Pradigastat (USAN)
D00277  7.294(2)  8.938  Cleocin (TN), Clindamycin (USAN/INN)
D02132  7.294(2)  8.938  Cleocin hydrochloride (TN), Dalacin (TN), Clindamycin hydrochloride (JP17/USP)
D04682  7.299(4)    -    Lecithin (NF)
D00184  7.815(2)  7.306  Gengraf (TN), Cyclosporine (USP), Sandimmune (TN), Neoral (TN), Ciclosporin (JP17), Restasis (TN)
D01844  7.308(2)  8.356  Arixtra (TN), Fondaparinux sodium (JAN/USP/INN)
D07711  7.313(3)  7.813  Clavulox (TN), CVA, Clavulanic acid (INN)
D00075  7.326(2)  7.322  Testosterone (JAN/USP), Axiron (TN), Androgel (TN), Testim (TN), Striant (TN), Androderm (TN)
D01921  7.326(2)  7.322  Estradiol and testosterone, Bothermon (TN)
D00736  7.324(3)    -    Syprine (TN), Trientine dihydrochloride, Trientine hydrochloride (JP17/USP)
D05218  7.718(2)  7.326  Ocrelizumab (genetical recombination) (JAN), Ocrevus (TN), Ocrelizumab (USAN)
D08124  7.326(3)    -    Levalbuterol, Levosalbutamol (INN)
D03068  7.333(2) 10.421  LymphoStat-B (TN), Belimumab (USAN), Benlysta (TN), Belimumab (genetical recombination) (JAN)
D02472  7.333(2)  9.037  Malarone (TN), Atovaquone and proguanil hydrochloride
D02487  7.333(2) 10.425  Paludrine (TN), Chloroguanide hydrochloride, Proguanil hydrochloride (JAN/USP)
D08428  7.333(2) 10.425  Proguanil (INN)
D05246  7.335(2)  9.740  Olmesartan (USAN/INN)
D00106  7.336(2)  8.076  Prostaglandin I2, Epoprostenol (USAN/INN), Prostacyclin, Epoprostenol (TN)
D11052  7.343(3)    -    Risankizumab (genetical recombination) (JAN), Risankizumab-rzaa, Skyrizi (TN), Risankizumab (USAN/INN)
D04747  7.343(3)    -    Lisdexamfetamine mesilate (JAN), Lisdexamfetamine dimesylate (USAN), Elvanse (TN), Vyvanse (TN)
D00275  7.344(5)  9.436  Platinol (TN), Cisplatin (JP17/USP/INN)
D01035  7.897(2)  7.346  Cepharanthine (JAN), Cepharanthine (TN)
D01778  7.957(2)  7.348  Bisolvon (TN), Bromhexine hydrochloride (JP17/USAN)
D07542  7.957(2)  7.348  Bromhexine (INN), Fluibron (TN)
D01345  7.434(2)  7.351  Dantroche hibitane (TN), Chlorhexidine hydrochloride (JP17/USP)
D04425  7.434(2)  7.351  Despa (TN), Diphenhydramine salicylate, hydrocortisone acetate, benzalkonium chloride and chlorhexidine hydrochloride
D07668  7.434(2)  7.351  Chlorhexidine (INN), Dentisept [veterinary] (TN), Merfen-incolore (TN)
D10909  7.359(3)  9.721  Bictegravir (USAN/INN)
D10910  7.359(3)  9.721  Bictegravir sodium (JAN/USAN)
D00421  7.361(2)  8.134  Altace (TN), Ramipril (USP/INN)
D02056  8.044(2)  7.366  Sodium chloride (JP17/USP), Adsorbanac (TN)
D02060  8.044(2)  7.366  Potassium chloride (JP17/USP), Klor-con (TN), Kaon-Cl (TN), KCL (TN), K-dur (TN), Klotrix (TN)
D02256  7.688(3)  7.366  Calcium chloride dihydrate, Calcium chloride (USP), Calcium chloride hydrate (JP17), Conclyte-Ca (TN)
D04424  7.806(2)  7.366  Saliveht (TN), Potassium phosphate, dibasic, potassium chloride, calcium chloride hydrate, sodium chloride and magnesium chloride
D04834  8.044(2)  7.366  Magnesium chloride (JAN/USP)
D04981  8.044(2)  7.366  Tromethamol, sodium chloride and potassium chloride, Tham Set (TN)
D08814  8.044(2)  7.366  Miotecter (TN), Potassium chloride, calcium chloride hydrate, sodium chloride, magnesium chloride and Sodium bicarbonate
D11174  8.044(2)  7.366  Movicol (TN), Macrogol 4000, sodium chloride, sodium bicarbonate and potassium chloride
D03353  7.376(2)  8.481  Fragmin (TN), Ardeparin sodium, Dalteparin sodium (JAN/USP/INN)
D00346  7.379(4)  9.882  Laniazid (TN), Isoniazid (JP17/USP/INN)
D02768  7.380(3)    -    Adefovir (USAN/INN)
D03053  7.381(2)  7.528  Adhesive bandage, Bandage, adhesive (USP)
D06006  7.381(2)  7.528  Tape, adhesive (USP)
D09327  7.382(4)    -    Karenitecin (TN), Cositecan (USAN)
D02598  7.428(2)  7.385  Infliximab (USAN/INN), Remicade (TN), Renflexis (TN), Infliximab-dyyb, Infliximab-axxq, Avsola (TN), Inflectra (TN), Infliximab-abda, Infliximab (genetical recombination) [Infliximab biosimilar 3] (JAN), Infliximab (genetical recombination) (JAN), Infliximab (genetical recombination) [Infliximab biosimilar 1] (JAN), Infliximab (genetical recombination) [Infliximab biosimilar 2] (JAN)
D11131  7.386(3)  9.720  Apabetalone (USAN/INN)
D08918  7.386(4)  9.196  CEA, MUC-1, fowlpox virus, Falimarev (USAN)
D02275  7.387(2)  9.636  Succin (TN), Suxamethonium chloride dihydrate, Suxamethonium chloride hydrate (JP17)
D10914  7.387(2)  9.636  Calcium succinate (USP), Calcium succinate monohydrate
D00765  7.390(4)    -    Zemuron (TN), Rocuronium bromide (JAN/USP/INN)
D02000  7.391(3)    -    Leucomycin tartrate (TN), Kitasamycin tartrate (JP17)
D00146  7.393(2)  9.165  Corticotropin (USP/INN), Acthar (TN), ACTH (TN)
D03229  7.394(2)  8.523  BLM, Bleo (TN), Bleomycin hydrochloride (JP17)
D07535  7.394(2)  8.523  BLM, Bleomycin (INN), Bleomycin Hexal (TN)
D06964  7.396(2)    -    Saikokeishito extract (JP17), Saikokeishito
D02040  7.402(3) 10.413  Pralmorelin hydrochloride (JAN), Pralmorelin dihydrochloride (USAN), GHRP (TN)
D00221  8.284(2)  7.404  Acetylcysteine (JP17/USP/INN), Mucomyst (TN)
D01711  7.404(2)  7.418  Sodium hypochlorite (JAN/USP), Texant (TN)
D01727  7.404(2)  7.418  Chlorinated lime (JP17), Calcium hypochlorite
D00058  7.405(4) 10.639  gamma-Aminobutyric acid (JAN), Gammalon (TN)
D01061  7.410(5)  9.437  Campto (TN), CPT 11, Irinotecan hydrochloride trihydrate, Irinotecan hydrochloride hydrate (JP17), CPT-11, Camptosar (TN), Irinotecan hydrochloride (USP), Onivyde (TN)
D08086  7.410(5)  9.437  Biotecan (TN), Irinotecan (INN)
D05295  7.417(3)  9.730  Smallpox vaccine (USP)
D04805  8.058(3)  7.421  Tocopherol and vitamin A, Juvela (TN)
D06543  8.414(2)  7.421  Aquasol A (TN), Vitamin A (USP), Vitamin A1, Retinol
D00027  7.428(2)  8.605  Uracil (JAN/USAN)
D02131  7.428(2)  8.605  Uftoral (TN), Tegafur and uracil
D09776  7.428(2)  8.605  Uracil (2-13C) (JAN)
D04990  7.429(3)  8.381  Stronger neo minophagen C (TN), Monoammonium glycyrrhizinate, glycine and L-cysteine hydrochloride
D00938  8.016(2)  7.434  Tricalcium phosphate, Posture (TN)
D03225  7.437(5)    -    Belotecan hydrochloride (USAN)
D06556  7.437(5)    -    Apilimod mesylate (USAN)
D08284  7.440(2)    -    (-)-Noradrenaline hydrochloride, Arterenol (TN), Norepinephrine hydrochoride
D12020  8.327(2)  7.440  Garlic
D05776  7.442(3)  9.127  Rupintrivir (USAN/INN)
D00116  7.442(3)  8.430  Glucagon (JAN/USP/INN), Glucagon (genetical recombination) (JAN), Glucagon (TN)
D02118  7.442(3)  8.430  Glucagon monohydrochloride, Glucagon hydrochloride, Glucagon (TN), Glucagen (TN)
D10814  7.445(3)  8.807  Glecaprevir (USAN/INN)
D10815  7.445(3)  8.807  Glecaprevir hydrate (JAN)
D11014  7.445(3)  8.807  Mavyret (TN), Glecaprevir and pibrentasvir
D00182  7.446(3)    -    Micronor (TN), Primolut-N (TN), Norethindrone (USP), Norethisterone (JP17), Camila (TN)
D04462  7.446(3)    -    Necon (TN), Norethisterone and mestranol
D02574  7.448(4)    -    Strattera (TN), Tomoxetine hydrochloride, Atomoxetine hydrochloride (JAN/USP)
D07782  7.450(2)    -    Delavirdine (INN)
D00345  7.453(4)    -    Natrix (TN), Indapamide (JP17/USP), Lozol (TN)
D06401  7.453(4)    -    Indapamide hydrate, Tenaxil (TN)
D02484  7.456(2)  9.196  Coartem (TN), Artemether and lumefantrine
D03821  7.456(2)  9.196  Lumefantrine (JAN/USP/INN)
D08754  7.468(2)    -    L-Arginine and L-arginine hydrochloride, Argi-U (TN)
D00696  8.112(2)  7.471  Midazolam hydrochloride (USAN), Versed (TN)
D00340  7.473(2) 10.492  Hydrochlorothiazide (JP17/USP/INN), Esidrix (TN), Microzide (TN)
D04265  7.473(2) 10.492  Reserpine, hydralazine hydrochloride and hydrochlorothiazide
D10267  7.473(2) 10.492  Methyldopa and hydrochlorothiazide, Methyldopa and hydrochlorothiazide (TN)
D10269  7.473(2) 10.492  Hydrochlorothiazide and triamterene, Dyazide (TN)
D10270  7.473(2)  8.183  Spironolactone and hydrochlorothiazide, Aldactazide (TN)
D10271  7.473(2)  9.835  Amiloride hydrochloride and hydrochlorothiazide (TN), Amiloride hydrochloride and hydrochlorothiazide
D10273  7.473(2) 10.492  Lopressor hct (TN), Dutoprol (TN), Metoprolol and hydrochlorothiazide
D10274  7.473(2)  9.677  Inderide (TN), Propranolol hydrochloride and hydrochlorothiazide (TN), Propranolol hydrochloride and hydrochlorothiazide
D10275  7.473(2) 10.492  Ziac (TN), Bisoprolol fumarate and hydrochlorothiazide
D10277  7.473(2) 10.492  Vaseretic (TN), Enalapril maleate and hydrochlorothiazide
D10278  7.473(2) 10.492  Quinapril hydrochloride and hydrochlorothiazide, Accuretic (TN)
D10279  7.473(2) 10.422  Lotensin hct (TN), Benazepril hydrochloride and hydrochlorothiazide
D10280  7.473(2) 10.492  Fosinopril sodium and hydrochlorothiazide (TN), Fosinopril and hydrochlorothiazide
D10281  7.473(2) 10.492  Uniretic (TN), Moexipril hydrochloride and hydrochlorothiazide
D10283  7.473(2) 10.492  Teveten htc (TN), Eprosartan mesylate and hydrochlorothiazide
D10284  7.473(2) 10.419  Olmesartan medoxomil and hydrochlorothiazide, Benicar hct (TN)
D10289  7.473(2) 10.492  Tekturna hct (TN), Aliskiren hemifumarate and hydrochlorothiazide
D10291  7.473(2)  9.724  Aliskiren hemifumarate, amlodipine besylate and hydrochlorothiazide, Amturnide (TN)
D02222  8.212(2)  7.475  Meropenem hydrate (JP17), Meropenem (USP), Merrem (TN)
D08185  8.212(2)  7.475  Meropenem (INN), Merrem I.V. (TN), Meronem (TN)
D11015  8.212(2)  7.475  Meropenem and vaborbactam, Vabomere (TN)
D01078  7.481(2) 10.424  Telithromycin (JAN/USAN/INN), Ketek (TN)
D00018  7.487(4)  7.842  ASCOR (TN), Ascorbic acid (JP17/USP/INN), Ascoltin (TN), Ascorbicap (TN)
D04899  7.487(4)  7.842  Thiamine chloride hydrochloride, riboflavin sodium phosphate and ascorbic acid
D04909  7.487(4)  7.842  Ascorbic acid and calcium pantothenate (JP17), Cinal (TN)
D04967  7.487(4)  7.842  Ophthalm K (TN), Carbazochrome, phytonadione and ascorbic acid
D08739  7.487(4)  7.842  L-Cysteine and ascorbic acid, Crystfan (TN)
D03260  7.491(4)    -    Levitra (TN), Vardenafil monohydrochloride trihydrate, Staxyn (TN), Vardenafil hydrochloride hydrate (JAN)
D06412  7.491(4)    -    Tekturna (TN), Aliskiren fumarate (JAN/USAN), Aliskiren hemifumarate
D00255  8.677(2)  7.491  Artist (TN), Carvedilol (JP17/USP/INN), Coreg (TN)
D00204  7.492(2)  9.075  Omnipen (TN), Ampicillin (USP/INN), Anhydrous ampicillin (JP17)
D01251  7.492(2)  9.075  Amcill (TN), Ampicillin trihydrate, Ampicillin hydrate (JP17)
D02065  7.492(2)  8.588  Ampicillin sodium and sulbactam sodium (JP17), Unasyn-S (TN)
D02119  7.492(2)  9.075  Omnipen-N (TN), Viccillin (TN), Ampicillin sodium (JP17/USP)
D02136  7.492(2)  9.075  Ampicillin and dicloxacillin sodium
D02502  7.492(2)  9.075  Viccillin S (TN), Ampicillin and cloxacillin
D10816  7.494(4)  9.723  Pibrentasvir (JAN/USAN/INN)
D02162  7.496(5)  9.722  Chlorprothixene hydrochloride (JAN)
D05096  7.496(2)  7.969  Mycophenolic acid (USAN/INN), Mycophenolic acid (TN)
D01994  7.501(5)    -    Mosapride citrate dihydrate, Gasmotin (TN), Mosapride citrate hydrate (JP17)
D00443  7.502(3)  8.183  Spironolactone (JP17/USP/INN), Aldactone (TN)
D00283  8.357(2)  7.507  Clozapine (JAN/USP/INN), Clozaril (TN)
D00066  7.508(4)  8.769  Progesterone (JP17/USP/INN), Prometrium (TN), Crinone (TN)
D04459  7.508(4)  8.284  Progesterone and estradiol, Lutes (TN)
D11137  7.508(2) 10.414  Larotrectinib (USAN/INN)
D01340  8.768(4)  7.509  Narcan (TN), Naloxone hydrochloride (JP17/USP), Evzio (TN)
D08249  8.768(4)  7.509  DBL Naloxone (TN), Naloxone (INN)
D11620  8.768(4)  7.509  Naloxone hydrochloride dihydrate
D00566  7.510(3)  9.567  Salsonin (TN), Sodium salicylate (JP17/USP)
D00827  7.510(3)  9.567  Magan (TN), Magnesium salicylate (USP), Magnesium salicylate tetrahydrate
D04016  7.510(3)  9.567  Camphor and sodium salicylate, Camphorin (TN)
D04022  7.510(3)  9.340  Sodium salicylate, dibucaine hydrocholoride and calcium bromide, Neo vitacain (TN)
D01029  7.513(3) 10.429  Aggrastat (TN), Tirofiban hydrochloride (USAN), Tirofiban hydrochloride hydrate
D03256  7.514(4) 10.428  Valganciclovir hydrochloride (JAN/USP), Valcyte (TN)
D04154  8.588(2)  7.515  Fennel oil, bitter, Fennel oil (JP17/NF)
D04388  8.588(2)  7.515  Orange peel, bitter, Dried bitter orange peel (TN), Bitter orange peel (JP17), Powdered bitter orange peel (Non-JPS)
D02743  7.517(5)    -    Celmoleukin (INN), Celeuk (TN), Celmoleukin (genetical recombination) (JP17)
D07445  7.520(3)  8.901  Amfetamin (TN), Dyanavel (TN), Amphetamine, Adzenys (TN), Amfetamine (INN)
D11036  7.520(3)  8.539  Glycopyrrolate and formoterol, Bevespi (TN)
D11042  7.520(3)  9.727  Tezacaftor and ivacaftor, Symdeko (TN)
D04765  7.520(3)    -    Lofexidine hydrochloride (USAN), Lucemyra (TN), Lofexidine (TN)
D02538  7.525(3)    -    Sodium nifurstyrenate
D00636  8.491(2)  7.526  Nexterone (TN), Amiodarone hydrochloride (JP17/USP), Ancaron (TN), Cordarone (TN)
D02910  8.491(2)  7.526  Amiodarone (USAN/INN)
D03753  7.528(2)    -    Perindopril (USAN/INN)
D03940  7.537(2)  7.601  Soliris (TN), Eculizumab (genetical recombination) (JAN), Eculizumab (USAN/INN)
D04014  7.543(3)    -    Enzastaurin hydrochloride (JAN/USAN)
D06574  7.543(3)    -    Casopitant mesylate (USAN)
D02485  7.544(2)  8.460  Halofantrine hydrochloride (USAN), Halfan (TN)
D08033  7.544(2)  8.460  Halofantrine (INN)
D04358  7.544(3) 10.428  Simponi (TN), Golimumab (USAN/INN), Golimumab (genetical recombination) (JAN)
D04843  7.545(3)    -    Malic acid (NF), Malate
D02179  7.548(2) 10.469  Hexa-betalin (TN), Pyridoxine hydrochloride (JP17/USP)
D04901  7.548(2) 10.427  Alithia N (TN), Thiamine disulfide, pyridoxine hydrochloride and cyanocobalamin
D04904  7.548(2) 10.469  Thiamine disulfide, pyridoxine hydrochloride and hydroxocobalamin acetate, Dai medine (TN)
D04911  7.548(2) 10.427  Benfotiamine, pyridoxine hydrochloride and cyanocobalamin, Vitamedin (TN)
D04913  7.548(2) 10.427  Daivitamix (TN), Thiamine chloride hydrochloride, pyridoxine hydrochloride and cyanocobalamin
D04916  7.548(2) 10.413  Tridocelan (TN), Thiamine nitrate, pyridoxine hydrochloride and hydroxocobalamin acetate
D04917  7.548(2)  8.386  Octotiamine, riboflavin, pyridoxine hydrochloride and cyanocobalamin, Neurovitan (TN)
D04930  7.548(2) 10.469  Vitamedin-S (TN), Benfotiamine, pyridoxine hydrochloride and Hydroxocobalamin hydrochloride
D04935  7.548(2) 10.427  Thiamine disulfide phosphate, pyridoxine hydrochloride and cyanocobalamin, Vitamedin (TN)
D04937  7.548(2)  8.386  Bifuroxin (TN), Riboflavin and pyridoxine hydrochloride
D08454  7.548(2)  9.776  Vitamin B6 (TN), Pyridoxine (INN)
D10357  7.548(2) 10.469  Doxylamine succinate and pyridoxine hydrochloride, Diclegis (TN)
D01276  7.550(4)    -    Atazanavir sulfate (JAN/USAN), Reyataz (TN)
D07450  7.551(2)  8.258  Norvasc (TN), Amlodipine (USP/INN)
D10290  7.551(2)  8.258  Tekamulo (TN), Rasilamlo (TN), Aliskiren and amlodipine
D11069  7.551(2)  8.258  Amlobenz (TN), Lotrel (TN), Amlodipine and benazepril
D11705  7.551(2)  8.258  Amlodipine and celecoxib, Consensi (TN)
D08707  7.555(3)  7.842  Bellsan (TN), l-Menthol, gentian, scopolia extract powder and sodium bicarbonate
D02597  7.557(3)  8.016  Hyrimoz (TN), Cyltezo (TN), Adalimumab-fkjp, Hadlima (TN), Humira (TN), Amjevita (TN), Adalimumab (USAN/INN), Adalimumab-afzb, Abrilada (TN), Adalimumab-adbm, Adalimumab (genetical recombination) [Adalimumab biosimilar 1] (JAN), Adalimumab (genetical recombination) (JAN), Adalimumab-atto, Adalimumab-adaz, Adalimumab-bwwd, Adalimumab (genetical recombination) [Adalimumab biosimilar 2] (JAN), Adalimumab (genetical recombination) [Adalimumab biosimilar 3] (JAN)
D04024  7.558(3)    -    Tyverb (TN), Lapatinib tosilate hydrate (JAN), Lapatinib ditosylate (USAN), Lapatinib ditosylate monohydrate, Tykerb (TN)
D03985  7.679(2)  7.558  Emetine hydrochloride (USP)
D01572  7.563(5) 10.421  Pam (TN), Pralidoxime iodide (JAN/USAN)
D03925  7.566(2)  8.015  Freeze-dried diphtheria antitoxin (TN), Freeze-dried diphtheria antitoxin, equine (JP17)
D05187  7.566(2)  8.015  Gas gangrene antitoxin, pentavalent, Freeze-dried gas gangrene antitoxin, equine (TN), Gas gangrene antitoxin, equine (JP17)
D05317  7.566(2)  8.015  Freeze-dried habu antivenom, equine (JP17)
D05368  7.566(2)  8.015  Freeze-dried botulism antitoxin, equine (JP17)
D05372  7.566(2)  8.015  Freeze-dried mamushi antivenom, equine (JP17)
D06513  7.566(2)  8.015  Freeze-dried tetanus antitoxin, equine (JP17)
D08808  7.566(2)  8.015  Anti-human thymocyte immunoglobulin, equine (JAN), Lymphoglobuline (TN)
D02280  7.568(3) 10.534  Ancoma (TN), Sodium L-glutamate hydrate (JAN), Monosodium L-glutamate monohydrate, Monosodium glutamate (NF)
D02714  7.569(4)  8.309  Zortress (TN), Afinitor (TN), Votubia (TN), Everolimus (JAN/USAN/INN)
D00014  7.569(2)  7.766  Tathion (TN), Glutathione (JP17), Glutathione (reduced type)
D00031  7.569(2)  7.766  Oxiglutatione (JAN/INN), Glutathione (TN)
D09403  7.571(5)    -    Custirsen sodium (USAN)
D00181  8.209(3)  7.577  Novastan (TN), Argatroban hydrate (JP17), Argatroban (USP)
D10064  7.577(4)  9.732  Dabrafenib (USAN)
D09332  7.577(3)    -    Denenicokin (USAN/INN)
D03145  8.292(2)  7.580  Boldenone undecylenate (USAN), Equipoise (TN)
D00201  7.584(2)  8.715  Tetracycline (JAN/USP/INN), Sumycin (TN)
D02122  7.584(2)  8.715  Tetracycline hydrochloride (JP17/USP), Bristacycline (TN)
D04775  7.584(2)  8.715  Tetra cortisone (TN), Tetracycline hydrochloride and hydrocortisone acetate
D04865  7.584(2)  8.715  Tetracycline hydrochloride and Epidihydrocholesterin, Tetracycline presteron (TN)
D10245  7.584(2)  8.521  Pylera (TN), Helidac (TN), Bismuth subcitrate, tetracycline and metronidazole
D00262  7.585(2) 10.471  Cefuroxime (TN), Cefuroxime (USAN/INN)
D00915  7.585(2) 10.471  Zinacef (TN), Cefuroxime sodium (JAN/USP)
D04536  7.586(2)  9.163  Fluarix quadrivalent (TN), Influenza virus vaccine (USP), FluShield (TN)
D02499  7.591(2)    -    Fuzeon (TN), Enfuvirtide (USAN/INN)
D03975  7.803(2)  7.593  Apronalide, Allylisopropylacetylurea, Apronal
D00141  7.593(2)  9.027  Indomethacin (USP), Indocin (TN), Indometacin (JP17/INN), Aconip (TN)
D02110  7.593(2)  9.027  Indocin I.V. (TN), Indomethacin sodium (USP), Indomethacin sodium trihydrate, Indometacin sodium hydrate (JAN), Indometacin sodium
D02214  8.008(5)  7.595  Ellence (TN), EPI, Epirubicin hydrochloride (JP17/USP)
D00626  7.597(3)    -    Atacand (TN), Candesartan cilexetil (JP17/USP)
D07569  7.597(3)    -    Unisia (TN), Candesartan cilexetil and anmlodipine besylate (JP17)
D02548  7.598(2)  9.242  Giardiasis (TN), Quinacrine hydrochloride anhydrous, Mepacrine dihydrochloride
D06088  7.599(2)  8.105  TAT, Tetanus antitoxin
D03899  8.214(5)  7.599  Adriblastina (TN), ADR, Doxorubicin (USAN/INN)
D00320  8.727(3)  7.602  Subsys (TN), Duragesic (TN), Fentanyl (JAN/USP/INN)
D10811  8.727(3)  7.602  Fentanyl hydrochloride (JAN), Ionsys (TN)
D00341  7.605(2)  8.738  Droxia (TN), Hydroxyurea (USP), Hydrea (TN), Hydroxycarbamide (JAN/INN)
D09643  7.881(2)  7.606  Myeloperoxidase (USAN)
D09012  7.607(3)  8.820  Incivek (TN), Incivo (TN), Telaprevir (JAN/USAN/INN)
D00954  7.607(3)    -    Norgestrel (JP17/USP/INN), Ovrette (TN)
D00937  7.806(2)  7.611  Calster (TN), Calcium phosphate, dihydrate, dibasic (USP), Dibasic calcium phosphate hydrate (JP17), Dibasic calcium phosphate (TN)
D02403  7.806(2)  7.611  Potassium phosphate, dibasic (JAN/USP), Mediject P (TN)
D03302  7.806(2)  7.611  Dibasic calcium phosphate, anhydrous (JAN), Calcium phosphate, dibasic, CalStar (TN)
D03303  7.806(2)  7.611  Calcium phosphate, tribasic (NF)
D04836  7.806(2)  7.611  Magnesium phosphate pentahydrate, Magnesium phosphate (USP)
D05869  7.806(2)  7.611  Sodium phosphate, dibasic (USP)
D06389  7.806(2)  7.611  Dibasic sodium phosphate hydrate (JP17), Phosphoric acid, disodiumsalt, dodecahydrate, Sodium phosphate, dibasic, dodecahydrate
D09000  7.806(2)  7.611  Sodium phosphate, tribasic (NF)
D05251  8.838(2)  7.612  Xolair (TN), Omalizumab (genetical recombination) (JAN), Omalizumab (USAN/INN)
D03370  7.614(2)  7.729  Polyethylene glycol (NF), Sentry polyox WSR (TN), PEG, Lutrol E (TN), Polyethylene glycol 3350 (USP), Macrogol, Polyethylene oxide (NF)
D00022  7.674(6)  7.615  Tyrosine (USP/INN), L-Tyrosine (JP17)
D01598  7.616(4)    -    Calcium L-aspartate hydrate (JAN), Calcium L-aspartate, Aspara-CA (TN)
D11936  7.618(3)  7.739  Bamlanivimab (USAN)
D08079  7.620(2) 10.134  Inositol (NF), Inosital (TN)
D03584  7.620(5)  9.720  Colforsin (USAN/INN)
D00285  7.624(2)  8.615  Co-Trimoxazole (BAN), Sulfamethoxazole and trimethoprim, Septra (TN), Bactrim (TN)
D00447  7.624(2)  8.886  Gantanol (TN), Sulfamethoxazole (JP17/USP/INN)
D00777  7.789(2)  7.625  Symmetrel (TN), Gocovri (TN), Osmolex er (TN), Amantadine hydrochloride (JP17/USP)
D07441  7.789(2)  7.625  Amantadine (INN)
D06670  7.625(2)  8.635  Maraviroc (JAN/INN), Selzentry (TN)
D01767  7.626(2)    -    Tilcotil (TN), Tenoxicam (JAN/USAN/INN)
D00900  7.627(2)  9.722  Tamiflu (TN), Oseltamivir phosphate (JAN/USP), Ebilfumin (TN)
D01261  8.140(2)  7.629  Fluorescein (JAN/USP), Fluorescite (TN)
D02024  8.140(2)  7.629  Floures (TN), Fluorescein sodium (JP17/USP)
D11645  8.140(2)  7.629  Fluorescein sodium and benoxinate hydrochloride, Altafluor benox (TN)
D03478  7.631(3)    -    Ovidrel (TN), Choriogonadotropin alfa (genetical recombination) (JAN), Choriogonadotropin alfa (USAN/INN)
D01038  7.631(2)  7.664  Hesperidin (JAN)
D10885  7.636(3) 10.414  Abaloparatide (USAN/INN), Tymlos (TN)
D01966  7.638(3)  8.508  Ezetimibe (JAN/USP/INN), Zetia (TN)
D10257  7.638(3)  8.508  Ezetimibe and simvastatin, Vytorin (TN)
D10385  7.638(3)  7.820  Liptruzet (TN), Ezetimibe and atorvastatin, Atozet (TN)
D11520  7.638(3)  8.508  Rosuzet (TN), Ezetimibe and rosuvastatin
D11794  7.638(3)  8.508  Nexlizet (TN), Bempedoic acid and ezetimibe
D02147  8.278(2)  7.638  Albuterol (USP), Salbutamol, Proventil (TN)
D10589  7.639(3)    -    Edarbyclor (TN), Azilsartan medoxomil and chlorthalidone
D05214  7.941(2)  7.647  Oatmeal, colloidal, Colloidial oatmeal (USP)
D06522  7.651(2)  7.647  Silicon dioxide, colloidal (NF), Silica
D00237  7.647(2) 10.427  Ridaura (TN), Auranofin (JP17/USAN/INN)
D01453  7.649(3)  8.948  Caffeine hydrate (JP17), Caffeine (TN), Caffeine monohydrate
D05956  7.649(3)    -    Sulfisoxazole acetyl (USP), Gantrisin (TN), Acetylsulfisoxazole (JAN), Lipo gantrisin (TN)
D03658  7.649(4)  8.258  Dasatinib (JAN/INN)
D06414  7.649(4)  8.258  Dasatinib hydrate (JAN), Sprycel (TN), Dasatinib (USAN)
D08336  8.175(2)  7.651  Peplomycin (INN), PEP
D05838  7.651(2)  8.580  Silica, dental-type (NF)
D00453  7.654(2)    -    Ofloxacin (JP17/USP/INN), Floxin (TN)
D08291  7.654(2)    -    Ofloxacin hydrochloride, Tarivid (TN)
D01583  8.038(3)  7.656  Esbriet (TN), Pirfenidone (JAN/USAN/INN)
D03829  7.656(2)  9.144  Peramivir (USAN/INN), Peramivir hydrate (JAN), Rapivab (TN)
D02295  7.820(2)  7.656  Povidone (JP17/USP/INN), Polyvinylpyrrolidone, Vinisil (TN)
D04008  7.657(3)  8.654  Baraclude (TN), Entecavir (USP), Entecavir hydrate (JAN)
D07896  7.657(3)  8.654  Entecavir (INN)
D02573  7.663(3)    -    Vestra (TN), Reboxetine mesilate, Reboxetine mesylate (USAN)
D02970  7.665(4)    -    Affinitak (TN), Aprinocarsen sodium (USAN)
D10899  7.671(4)    -    Voxilaprevir (USAN/INN)
D03802  7.671(4)    -    Tanomastat (USAN/INN)
D00145  7.672(2)  8.615  Trimpex (TN), Trimethoprim (JAN/USP/INN), Proloprim (TN)
D11085  7.672(2)  8.615  Trimpex (TN), Trimethoprim hydrochloride, Primsol (TN)
D11086  7.672(2)  7.738  Polytrim (TN), Polymyxin B and trimethoprim
D00731  7.673(2)  8.085  Milmag (TN), Magnesium hydroxide (JAN/USP), Mint-o-mag (TN), Magnesia, (Milk of) (USP)
D01083  7.673(2)  8.085  Calkyl (TN), Calcium hydroxide (JP17/USP)
D01168  7.673(2)  8.085  Potassium hydroxide (JP17/NF), Potassium hydroxide (TN)
D01169  7.673(2)  8.085  Sodium hydroxide (TN), Sodium hydroxide (JP17/NF)
D04393  7.673(2)  8.085  Aluminum hydroxide and magnesium hydroxide, Sakloft (TN), Maalox (TN)
D04856  7.673(2)  8.085  Calvital (TN), Sulfathiazole, diethylaminoethyl p-butylaminobenzoate hydrochloride, iodoform and calcium hydroxide
D08130  7.674(4)    -    Lisdexamfetamine (INN)
D11328  7.679(3)    -    Sunosi (TN), Solriamfetol hydrochloride (USAN)
D00208  7.680(5)  9.142  Mitomycin (USP/INN), Muamycin (TN), Mitomycin C (JP17), Mitomycin (TN), Jelmyto (TN), MMC
D03142  7.681(2)  7.737  Blood grouping serums anti-D, anti-C, anti-E, anti-c, anti-e (USP)
D01904  8.281(3)  7.681  Cefpiramide sodium (JP17/USAN), CPM, Suncefal (TN)
D03428  8.281(3)  7.681  Cefpiramide (USP/INN), CPM
D06547  7.681(2)  7.741  Tetanus toxoid (USP), TT
D00748  7.735(2)  7.684  Aldesleukin (USAN/INN), Proleukin (TN), Interleukin-2
D08302  7.685(3) 10.616  Ornithine (INN)
D10450  7.687(2) 10.419  Alecensa (TN), Alectinib hydrochloride (JAN)
D10542  7.687(2) 10.419  Alectinib (USAN/INN)
D00110  9.214(2)  7.695  Cocaine (TN), Cocaine (USP)
D02182  9.214(2)  7.695  Cocaine hydrochloride (TN), Cocaine hydrochloride (JP17/USP)
D00244  7.697(2)  8.526  Celestone (TN), Betamethasone (JP17/USP/INN), Rinderon (TN)
D04447  7.697(2)  8.526  Celestamine (TN), Betamethasone and d-chlorpheniramine maleate
D10300  7.697(2)  8.526  Clotrimazole and betamethasone, Lotrisone (TN)
D00067  7.698(3) 10.522  Estrone (TN), Estrone (JAN/USP/INN)
D04698  7.699(4)    -    Leteprinim potassium (USAN), Neotrofin (TN)
D09970  7.704(2)  7.865  Tofacitinib (USAN), Tasocitinib
D02993  7.705(4)    -    Arzoxifene hydrochloride (USAN)
D07299  7.705(4)    -    Vimpat (TN), Lacosamide (JAN/USAN/INN), Erlosamide
D08820  8.668(2)  7.708  Fresh frozen plasma -leukocytes reduced (FFP-LR), Fresh frozen plasma (FFP) (TN), Fresh-frozen human plasma
D08516  7.709(4)  8.365  Sitagliptin (prop.INN)
D11064  7.709(4)  8.365  Sitagliptin and ipragliflozin, Sujanu (TN)
D11066  7.709(4)  8.365  Steglujan (TN), Ertugliflozin and sitagliptin
D07413  7.711(5)    -    Pantothen Pharmaselect (TN), Pantothenic acid (BAN)
D05068  7.712(2)  9.089  Monensin (USP), Coban (TN)
D05069  7.712(2)  9.089  Monensin sodium (USP)
D08228  7.712(2)  9.089  Monensin (INN), Elancoban [veterinary] (TN)
D00614  7.713(2)    -    Sodium nitroprusside (USP), Sodium nitroprusside hydrate (JAN), Nitropress (TN)
D02142  7.713(2)  7.906  Tagocid (TN), Teicoplanin (JP17/USAN/INN)
D08166  7.714(3) 10.494  Farlutal inyectable (TN), Medroxyprogesterone (INN)
D01139  7.719(2)  9.082  Ammonium chloride (JAN/USP), Conclyte-A (TN)
D04554  7.721(2)  7.767  Interferon beta-1a (USAN), Interferon beta-1a (genetical recombination) (JAN), Avonex (TN), Rebif (TN)
D01370  8.069(3)  7.722  Leustatin (TN), Cladribine (JAN/USP/INN), Mavenclad (TN)
D02549  7.724(3)    -    Rifamycin (USAN/INN), Rifamycin SV
D08480  7.724(3)    -    Rifocina (TN), Rifamycin sodium (USAN), Aemcolo (TN), Rifamycin SV sodium salt, Otofa (TN)
D01559  7.967(4)  7.729  Ethyl L-cysteine hydrochloride (JP17), L-Cysteine, ethyl ester, hydrochloride, Daiace (TN)
