# Supplementary material for: A novel method of literature mining to identify candidate COVID-19 drugs
Source: Bioinform Adv. 2021 Jul 22;1(1):vbab013. doi: 10.1093/bioadv/vbab013 (PMC9710631; doi:10.1093/bioadv/vbab013)
Supplement: vbab013_Supplementary_Data [file vbab013_supplementary_data.zip › TableS3_muramatsu.rtf]

Table S3. Distances between COVID-19 and each drug calculated by the conditional probability method (first 1000 items).
Code: KEGG code, D_id: indirect (predicted) distance, D_d: direct (co-existing) distance

Code    D_id(Dpt)  D_d   Name
---------------------------------------------------------------------------------------------------------------
D05864  2.711(2)  1.641  Sodium monofluorophosphate (USP), Aim (TN)
D03043  2.206(2)  1.725  Air, medical (USP), Air (TN)
D08266  2.132(2)  1.856  Lactitol (NF/INN), Pizensy (TN), Importal (TN)
D03045  2.179(2)  2.033  Attapulgite, activated (USP), Parepectolin (TN)
D03251  2.179(2)  2.033  Medicinal carbon (TN), Medicinal carbon (JP17), Charcoal, activated (USP)
D07422  2.132(2)  2.321  Fibrinolysin, human, Fibrinolysin (human) (INN), Fibrogammin (TN)
D00028  2.206(2)  3.839  Glycerol (INN), Glycerin, concentrated (JAN), Concentrated glycerin (JP17), Glycerin (JP17/USP)
D04380  2.206(2)  4.581  Dried yeast (JP17), Ebios (TN), Yeast, dried
D05439  2.206(2)  2.929  Imagent (TN), Perflubron (USP/INN)
D08780  2.206(2)  3.839  Human C1 inactivator, freeze-dried concentrated, Berinert P (TN)
D08795  2.206(2)  3.839  Human anti-thrombin III, freeze-dried concentrated, Neuart (TN)
D08796  2.206(2)  3.839  Human activated protein C, freeze-dried concentrated, Anact C (TN)
D10884  2.206(2)  3.839  Human prothrombin complex, freeze-dried concentrated
D11965  2.206(2)  3.839  alpha 1-Antitrypsin, Lynspad (TN), Human alpha 1-Proteinase inibitor, freeze-dried concentrated
D00074  2.250(3)  6.560  INOmax (TN), Nitric oxide (JAN/USAN)
D09578  2.339(3)  5.047  Albumin human, recombinant (NF)
D06458  2.856(2)  2.364  Human normal immunoglobulin (JP17), Bay gam (TN), Hyqvia (TN), Gamma globulin (TN), Globulin, immune (USP)
D05258  2.569(4)  3.396  Serum gonadotrophin (JAN), Gonadotrophin, serum (JAN/INN), Anteron (TN)
D03143  2.604(3)  2.755  Blood, whole (USP)
D03237  2.604(3)  3.285  Adsorbin (TN), Aluminum silicate, natural (JAN), Natural aluminum silicate (JP17)
D05043  2.604(3)  3.940  Light liquid paraffin (JP17), Mineral oil, light (NF)
D03344  3.519(2)  2.644  Silver protein (TN), Silver protein, mild, Silver protein (JP17)
D07943  2.683(3)  2.973  Sensit (TN), Fendiline hydrochloride
D00001  2.717(3)  4.829  Sterile water (TN), Sterile purified water in containers (JP17), Water for injection (JP17), Purified water in containers (JP17), Water (JP17/USP), Water for injection in containers (JP17), Purified water (JP17), Water, purified (USP)
D00009  2.717(3)  4.935  D-Glucose, Purified glucose (JP17), Glucose (JP17)
D00025  2.717(3)  6.150  Sugar, compressible (NF), White soft sugar (JP17), Sucrose, purified, Sugar, confectioner's (NF), Sucrose (JP17/NF), Sucrose (TN), Sugar spheres (NF)
D02019  2.717(3)  4.935  Magnesium sulfate hydrate and glucose, Magnesol (TN)
D02325  2.717(3)  4.935  Glucose hydrate (JP17), Dextrose monohydrate, alpha-D-Glucose monohydrate, Cartose (TN), Dextrose (USP)
D03597  2.717(3)  6.150  Cotton, purified (USP)
D04109  2.717(3)  4.935  Opeguard MA (TN), Glucose and inorganic salt
D04337  2.717(3)  4.935  Glucose, liquid (JAN/NF), Glucose-40 (TN), Liquid glucose
D04963  2.717(3)  4.935  Dextran 40 and glucose, Dextron (TN)
D04978  2.717(3)  4.935  Acid -Citrate -Dextrose solution, ACD-A (TN), ACD-A solution, Sodium citrate hydrate, citric acid hydrate and glucose
D05707  2.717(3)  6.150  Rayon, purified (USP), Rayon
D05839  2.717(3)  6.150  Siliceous earth, purified (NF)
D06459  2.717(3)  6.150  Purified human menopausal gonadotrophin (JAN), Human menopausal gonadotrophin, purified
D06776  2.717(3)  6.150  Honey, purified (NF), Honey (JP17), Honey (TN)
D08741  2.717(3)  4.935  Karmi CA (TN), Adenine, sodium citrate hydrate, citric acid hydrate, glucose and potassium phosphate, monobasic, CPDA solution
D08742  2.717(3)  4.935  Sodium citrate hydrate, citric acid hydrate, glucose and potassium phosphate, monobasic, Karmi C (TN), CPD solution
D08743  2.717(3)  4.935  MAP solution, D-Mannitol, adenine, potassium phosphate, monobasic, sodium citrate hydrate, citric acid hydrate, glucose and sodium chloride
D03931  2.717(3)    -    Ecallantide (USAN/INN), Ecallantide (genetical recombination) (JAN), Kalbitor (TN)
D07508  2.759(3)  3.880  Mediator (TN), Benfluorex hydrochloride
D04133  2.766(3)  5.294  Fat, hard (NF)
D04845  2.766(3)    -    Maltitol (NF)
D00022  2.774(3)  7.062  L-Tyrosine (JP17), Tyrosine (USP/INN)
D08170  2.774(3)  7.135  Melatobel (TN), Melatonin (JAN), Melatonina (TN)
D01505  2.780(2)  4.891  Talipexole hydrochloride (JAN), Domin (TN)
D08446  2.780(2)  4.891  Prothipendyl hydrochloride, Dominal (TN)
D04969  2.806(2)  3.785  White beeswax (JP17), White wax (TN), Wax, white (NF)
D05239  2.806(2)  3.785  White ointment (JP17), Ointment, white (USP)
D05304  2.806(2)  3.785  White petrolatum (JP17), Moroline (TN), Petrolatum, white (USP)
D01032  2.821(4)  9.496  Agar (TN), Agar (JP17/NF), Powdered agar (JP17)
D10211  3.054(4)  2.828  Rotavirus vaccine, live, oral, pentavalent, Rotateq (TN)
D05318  2.852(4)  9.719  Potato starch (JP17), Starch, potato (NF), Potato starch (TN)
D10297  2.852(4)  9.496  Theophylline in dextrose (TN), Theophylline and dextrose
D08802  2.856(3)  4.581  Fibrogammin P (TN), Human blood-coagulation factor XIII fraction, dried
D10156  3.536(4)  2.887  Romosozumab (USAN), Romosozumab-aqqg, Evenity (TN), Romosozumab (genetical recombination) (JAN)
D00548  2.892(2)  5.124  Etomidate (USP/INN), Amidate (TN)
D02916  2.892(3)  3.225  Ammonia, Ammonia solution, strong (NF)
D05765  2.892(3)  3.225  Rose water , stronger (NF), Rose water, strong (NF)
D00056  2.899(3)  6.414  Parenzyme (TN), Trypsin (JAN), Trypsin, crystallized (USP)
D04664  2.899(3)  3.807  Wool wax, Lanolin, modified (USP), Hydrous lanolin (JP17), Lanolin (TN)
D04756  2.899(3)  6.414  Francetin T (TN), Fradiomycin sulfate and trypsin, crystallized
D07747  2.899(3)  5.274  alpha, beta-Pinene, borneol, anetholtrithion, d-camphene, cineole and fenchone, Rowatin (TN)
D08547  2.899(3)  4.800  Vessel (TN), Sulodexide (INN)
D08753  2.899(3)  6.414  Bromelains and trypsin, crystallized, Kimotab (TN)
D00943  2.912(3)  9.314  Fluorinse (TN), Sodium fluoride (JAN/USP)
D08736  2.912(3)  9.314  Fluoden A (TN), Sodium fluoride and phosphoric acid
D00003  2.917(3)  3.074  Oxygen (JP17/USP)
D03841  2.917(3)  3.074  Nitrous oxide and oxygen, Anesoxyn (TN)
D02056  2.940(4)  6.822  Sodium chloride (JP17/USP), Adsorbanac (TN)
D02060  2.940(4)  6.822  Klor-con (TN), Potassium chloride (JP17/USP), KCL (TN), Klotrix (TN), K-dur (TN), Kaon-Cl (TN)
D02256  2.940(4)  6.822  Calcium chloride (USP), Calcium chloride dihydrate, Calcium chloride hydrate (JP17), Conclyte-Ca (TN)
D04424  2.940(4)  6.822  Potassium phosphate, dibasic, potassium chloride, calcium chloride hydrate, sodium chloride and magnesium chloride, Saliveht (TN)
D04834  2.940(4)  6.822  Magnesium chloride (JAN/USP)
D04981  2.940(4)  6.822  Tromethamol, sodium chloride and potassium chloride, Tham Set (TN)
D06249  2.940(4)  4.829  Tritiotope (TN), Tritiated water, Water, tritiated (USAN)
D06286  2.940(4)  6.024  Hydrogenated vegetable oil, Vegetable oil, hydrogenated (NF)
D07703  2.940(4)  5.891  Hydroxyethyl cellulose, boric acid, dibasic sodium phosphate, potassium chloride, sodium chloride and dried sodium carbonate, Scopisol (TN)
D08810  2.940(4)  6.822  Quinine hydrochloride, sodium chloride, tartaric acid and sucrose
D08812  2.940(4)  6.773  Beriplast P combi-set (TN), Aprotinin, thrombin, human blood-coagulation factor XIII fraction, calcium chloride hydrate and freeze-dried human fibrinogen, Factor XIII with fibrinogen
D08814  2.940(4)  6.822  Potassium chloride, calcium chloride hydrate, sodium chloride, magnesium chloride and Sodium bicarbonate, Miotecter (TN)
D08816  2.940(4)  4.581  Niflec (TN), Potassium chloride, sodium chloride, sodium bicarbonate and sodium sulfate, dried
D09004  2.940(4)  6.024  Soybean oil, hydrogenated (NF)
D11174  2.940(4)  6.822  Macrogol 4000, sodium chloride, sodium bicarbonate and potassium chloride, Movicol (TN)
D11552  2.940(4)  6.822  Polyethylene Glycol 3350, sodium sulfate, sodium chloride, potassium chloride, sodium ascorbate and ascorbic acid, Plenvu (TN), Moviprep (TN)
D01167  2.940(4)  5.010  Magnesium oxide (JP17/USP), Magmitt (TN)
D01679  2.940(4)  5.010  Lime (USP), Calcium oxide (JP17)
D03436  2.940(4)  5.010  Surgicel (TN), Cellulose, oxidized (JAN/USP)
D04364  2.940(4)  4.581  Kolantyl (TN), Dicyclomine hydrochloride, aluminium hydroxide, dried and magnesium oxide
D08696  2.940(4)  5.010  Asgen (TN), Acetaminophen, ephedra herb, scopolia extract, caffeine and sodium benzoate and magnesium oxide
D10793  2.940(4)  5.010  Clenpiq (TN), Sodium picosulfate hydrate, magnesium oxide and citric acid, anhydrous, Prepopik (TN), Picoprep (TN)
D02549  2.953(3)    -    Rifamycin (USAN/INN), Rifamycin SV
D08480  2.953(3)    -    Rifamycin SV sodium salt, Rifamycin sodium (USAN), Rifocina (TN), Otofa (TN), Aemcolo (TN)
D00217  2.989(3)  7.333  Tylenol (TN), Paracetamol (INN), Acetaminophen (JP17/USP)
D00846  2.989(3)  7.333  Hydrocodone bitartrate and acetaminophen, Hydrocodone bitartrate and paracetamol, Vicodin (TN)
D02146  2.989(3)  7.333  Tylenol w/codeine (TN), Acetaminophen and codeine phosphate
D02153  2.989(3)  7.333  Tylox (TN), Percocet (TN), Roxicet 5/500 (TN), Acetaminophen and oxycodone hydrochloride
D03976  2.989(3)  7.333  SG (TN), Isopropylantipyrine, allyl isopropyl acetyl urea, acetaminophen and anhydrous caffeine
D04044  2.989(3)  7.333  Chlorpheniramine maleate, acetaminophen, salicylamide and anhydrous caffeine, LL (TN)
>>> for item in merged_list_formatted[0:500]: print(item)
... 
D05864  2.711(2)  1.641  Sodium monofluorophosphate (USP), Aim (TN)
D03043  2.206(2)  1.725  Air, medical (USP), Air (TN)
D08266  2.132(2)  1.856  Lactitol (NF/INN), Pizensy (TN), Importal (TN)
D03045  2.179(2)  2.033  Attapulgite, activated (USP), Parepectolin (TN)
D03251  2.179(2)  2.033  Medicinal carbon (TN), Medicinal carbon (JP17), Charcoal, activated (USP)
D07422  2.132(2)  2.321  Fibrinolysin, human, Fibrinolysin (human) (INN), Fibrogammin (TN)
D00028  2.206(2)  3.839  Glycerol (INN), Glycerin, concentrated (JAN), Concentrated glycerin (JP17), Glycerin (JP17/USP)
D04380  2.206(2)  4.581  Dried yeast (JP17), Ebios (TN), Yeast, dried
D05439  2.206(2)  2.929  Imagent (TN), Perflubron (USP/INN)
D08780  2.206(2)  3.839  Human C1 inactivator, freeze-dried concentrated, Berinert P (TN)
D08795  2.206(2)  3.839  Human anti-thrombin III, freeze-dried concentrated, Neuart (TN)
D08796  2.206(2)  3.839  Human activated protein C, freeze-dried concentrated, Anact C (TN)
D10884  2.206(2)  3.839  Human prothrombin complex, freeze-dried concentrated
D11965  2.206(2)  3.839  alpha 1-Antitrypsin, Lynspad (TN), Human alpha 1-Proteinase inibitor, freeze-dried concentrated
D00074  2.250(3)  6.560  INOmax (TN), Nitric oxide (JAN/USAN)
D09578  2.339(3)  5.047  Albumin human, recombinant (NF)
D06458  2.856(2)  2.364  Human normal immunoglobulin (JP17), Bay gam (TN), Hyqvia (TN), Gamma globulin (TN), Globulin, immune (USP)
D05258  2.569(4)  3.396  Serum gonadotrophin (JAN), Gonadotrophin, serum (JAN/INN), Anteron (TN)
D03143  2.604(3)  2.755  Blood, whole (USP)
D03237  2.604(3)  3.285  Adsorbin (TN), Aluminum silicate, natural (JAN), Natural aluminum silicate (JP17)
D05043  2.604(3)  3.940  Light liquid paraffin (JP17), Mineral oil, light (NF)
D03344  3.519(2)  2.644  Silver protein (TN), Silver protein, mild, Silver protein (JP17)
D07943  2.683(3)  2.973  Sensit (TN), Fendiline hydrochloride
D00001  2.717(3)  4.829  Sterile water (TN), Sterile purified water in containers (JP17), Water for injection (JP17), Purified water in containers (JP17), Water (JP17/USP), Water for injection in containers (JP17), Purified water (JP17), Water, purified (USP)
D00009  2.717(3)  4.935  D-Glucose, Purified glucose (JP17), Glucose (JP17)
D00025  2.717(3)  6.150  Sugar, compressible (NF), White soft sugar (JP17), Sucrose, purified, Sugar, confectioner's (NF), Sucrose (JP17/NF), Sucrose (TN), Sugar spheres (NF)
D02019  2.717(3)  4.935  Magnesium sulfate hydrate and glucose, Magnesol (TN)
D02325  2.717(3)  4.935  Glucose hydrate (JP17), Dextrose monohydrate, alpha-D-Glucose monohydrate, Cartose (TN), Dextrose (USP)
D03597  2.717(3)  6.150  Cotton, purified (USP)
D04109  2.717(3)  4.935  Opeguard MA (TN), Glucose and inorganic salt
D04337  2.717(3)  4.935  Glucose, liquid (JAN/NF), Glucose-40 (TN), Liquid glucose
D04963  2.717(3)  4.935  Dextran 40 and glucose, Dextron (TN)
D04978  2.717(3)  4.935  Acid -Citrate -Dextrose solution, ACD-A (TN), ACD-A solution, Sodium citrate hydrate, citric acid hydrate and glucose
D05707  2.717(3)  6.150  Rayon, purified (USP), Rayon
D05839  2.717(3)  6.150  Siliceous earth, purified (NF)
D06459  2.717(3)  6.150  Purified human menopausal gonadotrophin (JAN), Human menopausal gonadotrophin, purified
D06776  2.717(3)  6.150  Honey, purified (NF), Honey (JP17), Honey (TN)
D08741  2.717(3)  4.935  Karmi CA (TN), Adenine, sodium citrate hydrate, citric acid hydrate, glucose and potassium phosphate, monobasic, CPDA solution
D08742  2.717(3)  4.935  Sodium citrate hydrate, citric acid hydrate, glucose and potassium phosphate, monobasic, Karmi C (TN), CPD solution
D08743  2.717(3)  4.935  MAP solution, D-Mannitol, adenine, potassium phosphate, monobasic, sodium citrate hydrate, citric acid hydrate, glucose and sodium chloride
D03931  2.717(3)    -    Ecallantide (USAN/INN), Ecallantide (genetical recombination) (JAN), Kalbitor (TN)
D07508  2.759(3)  3.880  Mediator (TN), Benfluorex hydrochloride
D04133  2.766(3)  5.294  Fat, hard (NF)
D04845  2.766(3)    -    Maltitol (NF)
D00022  2.774(3)  7.062  L-Tyrosine (JP17), Tyrosine (USP/INN)
D08170  2.774(3)  7.135  Melatobel (TN), Melatonin (JAN), Melatonina (TN)
D01505  2.780(2)  4.891  Talipexole hydrochloride (JAN), Domin (TN)
D08446  2.780(2)  4.891  Prothipendyl hydrochloride, Dominal (TN)
D04969  2.806(2)  3.785  White beeswax (JP17), White wax (TN), Wax, white (NF)
D05239  2.806(2)  3.785  White ointment (JP17), Ointment, white (USP)
D05304  2.806(2)  3.785  White petrolatum (JP17), Moroline (TN), Petrolatum, white (USP)
D01032  2.821(4)  9.496  Agar (TN), Agar (JP17/NF), Powdered agar (JP17)
D10211  3.054(4)  2.828  Rotavirus vaccine, live, oral, pentavalent, Rotateq (TN)
D05318  2.852(4)  9.719  Potato starch (JP17), Starch, potato (NF), Potato starch (TN)
D10297  2.852(4)  9.496  Theophylline in dextrose (TN), Theophylline and dextrose
D08802  2.856(3)  4.581  Fibrogammin P (TN), Human blood-coagulation factor XIII fraction, dried
D10156  3.536(4)  2.887  Romosozumab (USAN), Romosozumab-aqqg, Evenity (TN), Romosozumab (genetical recombination) (JAN)
D00548  2.892(2)  5.124  Etomidate (USP/INN), Amidate (TN)
D02916  2.892(3)  3.225  Ammonia, Ammonia solution, strong (NF)
D05765  2.892(3)  3.225  Rose water , stronger (NF), Rose water, strong (NF)
D00056  2.899(3)  6.414  Parenzyme (TN), Trypsin (JAN), Trypsin, crystallized (USP)
D04664  2.899(3)  3.807  Wool wax, Lanolin, modified (USP), Hydrous lanolin (JP17), Lanolin (TN)
D04756  2.899(3)  6.414  Francetin T (TN), Fradiomycin sulfate and trypsin, crystallized
D07747  2.899(3)  5.274  alpha, beta-Pinene, borneol, anetholtrithion, d-camphene, cineole and fenchone, Rowatin (TN)
D08547  2.899(3)  4.800  Vessel (TN), Sulodexide (INN)
D08753  2.899(3)  6.414  Bromelains and trypsin, crystallized, Kimotab (TN)
D00943  2.912(3)  9.314  Fluorinse (TN), Sodium fluoride (JAN/USP)
D08736  2.912(3)  9.314  Fluoden A (TN), Sodium fluoride and phosphoric acid
D00003  2.917(3)  3.074  Oxygen (JP17/USP)
D03841  2.917(3)  3.074  Nitrous oxide and oxygen, Anesoxyn (TN)
D02056  2.940(4)  6.822  Sodium chloride (JP17/USP), Adsorbanac (TN)
D02060  2.940(4)  6.822  Klor-con (TN), Potassium chloride (JP17/USP), KCL (TN), Klotrix (TN), K-dur (TN), Kaon-Cl (TN)
D02256  2.940(4)  6.822  Calcium chloride (USP), Calcium chloride dihydrate, Calcium chloride hydrate (JP17), Conclyte-Ca (TN)
D04424  2.940(4)  6.822  Potassium phosphate, dibasic, potassium chloride, calcium chloride hydrate, sodium chloride and magnesium chloride, Saliveht (TN)
D04834  2.940(4)  6.822  Magnesium chloride (JAN/USP)
D04981  2.940(4)  6.822  Tromethamol, sodium chloride and potassium chloride, Tham Set (TN)
D06249  2.940(4)  4.829  Tritiotope (TN), Tritiated water, Water, tritiated (USAN)
D06286  2.940(4)  6.024  Hydrogenated vegetable oil, Vegetable oil, hydrogenated (NF)
D07703  2.940(4)  5.891  Hydroxyethyl cellulose, boric acid, dibasic sodium phosphate, potassium chloride, sodium chloride and dried sodium carbonate, Scopisol (TN)
D08810  2.940(4)  6.822  Quinine hydrochloride, sodium chloride, tartaric acid and sucrose
D08812  2.940(4)  6.773  Beriplast P combi-set (TN), Aprotinin, thrombin, human blood-coagulation factor XIII fraction, calcium chloride hydrate and freeze-dried human fibrinogen, Factor XIII with fibrinogen
D08814  2.940(4)  6.822  Potassium chloride, calcium chloride hydrate, sodium chloride, magnesium chloride and Sodium bicarbonate, Miotecter (TN)
D08816  2.940(4)  4.581  Niflec (TN), Potassium chloride, sodium chloride, sodium bicarbonate and sodium sulfate, dried
D09004  2.940(4)  6.024  Soybean oil, hydrogenated (NF)
D11174  2.940(4)  6.822  Macrogol 4000, sodium chloride, sodium bicarbonate and potassium chloride, Movicol (TN)
D11552  2.940(4)  6.822  Polyethylene Glycol 3350, sodium sulfate, sodium chloride, potassium chloride, sodium ascorbate and ascorbic acid, Plenvu (TN), Moviprep (TN)
D01167  2.940(4)  5.010  Magnesium oxide (JP17/USP), Magmitt (TN)
D01679  2.940(4)  5.010  Lime (USP), Calcium oxide (JP17)
D03436  2.940(4)  5.010  Surgicel (TN), Cellulose, oxidized (JAN/USP)
D04364  2.940(4)  4.581  Kolantyl (TN), Dicyclomine hydrochloride, aluminium hydroxide, dried and magnesium oxide
D08696  2.940(4)  5.010  Asgen (TN), Acetaminophen, ephedra herb, scopolia extract, caffeine and sodium benzoate and magnesium oxide
D10793  2.940(4)  5.010  Clenpiq (TN), Sodium picosulfate hydrate, magnesium oxide and citric acid, anhydrous, Prepopik (TN), Picoprep (TN)
D02549  2.953(3)    -    Rifamycin (USAN/INN), Rifamycin SV
D08480  2.953(3)    -    Rifamycin SV sodium salt, Rifamycin sodium (USAN), Rifocina (TN), Otofa (TN), Aemcolo (TN)
D00217  2.989(3)  7.333  Tylenol (TN), Paracetamol (INN), Acetaminophen (JP17/USP)
D00846  2.989(3)  7.333  Hydrocodone bitartrate and acetaminophen, Hydrocodone bitartrate and paracetamol, Vicodin (TN)
D02146  2.989(3)  7.333  Tylenol w/codeine (TN), Acetaminophen and codeine phosphate
D02153  2.989(3)  7.333  Tylox (TN), Percocet (TN), Roxicet 5/500 (TN), Acetaminophen and oxycodone hydrochloride
D03976  2.989(3)  7.333  SG (TN), Isopropylantipyrine, allyl isopropyl acetyl urea, acetaminophen and anhydrous caffeine
D04044  2.989(3)  7.333  Chlorpheniramine maleate, acetaminophen, salicylamide and anhydrous caffeine, LL (TN)
D04046  2.989(3)  7.333  Salicylamide, acetaminophen, anhydrous caffeine and promethazine methylenedisalicylate, PL (TN)
D08695  2.989(3)  7.333  Coughcode N (TN), dl-Methylephedrine hydrochloride, dihydrocodeine phosphate, diprophylline, diphenhydramine salicylate, acetaminophen and bromovalerylurea
D09999  2.989(3)  7.333  Tramcet (TN), Tramadol hydrochloride and acetaminophen
D11561  2.989(3)  7.333  Tencon (TN), Allzital (TN), Butalbital and acetaminophen, Butapap (TN), Bupap (TN)
D11628  2.989(3)  7.333  Apadaz (TN), Benzhydrocodone and acetaminophen
D11850  2.989(3)  7.333  Butalbital, acetaminophen and caffeine
D11851  2.989(3)  7.333  Butalbital, acetaminophen, caffeine and codeine phosphate
D11897  2.989(3)  7.214  Acetaminophen and ibuprofen
D04543  2.993(3)  4.175  Insulin human zinc, extended (USP)
D04549  2.993(3)  4.175  Insulin zinc, extended (USP), Ultralente iletin (TN)
D08711  3.030(5)  5.010  Coptis rhizome, senna leaf, rhubarb, magnesium oxide and magnesium sulfate hydrate, Cetilo (TN)
D00032  3.032(3)  8.719  L-Histidine (JP17), Histidine (USP/INN)
D01126  3.053(3)  7.620  R-gene (TN), L-Arginine hydrochloride (JP17), Arginine hydrochloride (USP)
D02300  3.053(4)  7.442  Adenosine 5'-triphosphate disodium, ATP (TN), Adenosine triphosphate disodium hydrate (JAN), Adenosine triphosphate disodium trihydrate
D02982  3.053(3)  7.620  L-Arginine (JP17), Arginine (USP/INN)
D06483  3.053(3)  7.620  R-gene (TN), Arginine hydrochloride
D08646  3.053(4)  7.442  ATP, Triphosadenine (DCF)
D10193  3.054(4)  4.114  Live attenuated human rota virus vaccine, oral, Rotarix (TN)
D00059  3.068(3)  9.314  Levodopa (JP17/USP/INN), Dopar (TN)
D00253  3.068(3)  9.314  Sinemet (TN), Parcopa (TN), Carbidopa hydrate and levodopa
D02135  3.068(3)  9.314  Madopar (TN), Levodopa and benserazide hydrochloride
D10293  3.068(3)  9.314  Carbidopa, levodopa and entacapone, Stalevo (TN)
D05401  3.081(3)  7.045  Octocog alfa (INN), Octocog alfa (genetical recombination) (JAN), Factor VIII (rDNA), Kogenate FS (TN)
D05873  3.084(3) 11.105  Sodium pyrophosphate (USAN)
D08765  3.084(3) 11.105  Sodium pyrophosphate hydrate and technetium (99mTc) pyrophosphate, Techne pyrophosphate (TN)
D09025  3.093(3)  6.927  Triglycerides, medium-chain (NF)
D02112  3.095(3)  4.973  Liquemin sodium (TN), Parnaparin sodium (INN), Heparin sodium (JP17/USP/INN)
D04427  3.095(3)  4.973  Calciparin, Calciparine (TN), Nadroparin calcium (INN), Heparin calcium (JP17)
D07510  3.095(3)  4.973  Enoxaparin, Semuloparin (USAN), Heparin (TN), Bemiparin, Nadroparin, Heparin (BAN), Parnaparin, Adomiparin (USAN)
D00024  3.122(3)  5.555  Bensulfoid (TN), Sulfur, precipitated (USP), Sastid (TN), Sulfur, sublimed (USP), Sulfur (JP17)
D04319  3.122(3)  4.554  Glaze, pharmaceutical (NF)
D04677  3.122(3)  8.272  Circanetten (TN), Paraphlebon, senna powder, sulfur and potassium hydrogen tartrate
D04811  3.122(3)  8.272  Sulfur and camphor (TN), Sulfur and camphor (JP17)
D05574  3.122(3)  8.272  Sulfurated potash, Potash, sulfurated (USP)
D08710  3.122(3)  7.872  Sulfur, fennel, glycyrrhiza and senna leaf, Compound glycyrrhiza (TN)
D00633  3.142(3)  8.708  Dopamine hydrochloride (JP17/USP), Actopamin (TN), Intropin (TN)
D07870  3.142(3)  8.708  Medopa (TN), Dopamine (INN)
D00040  3.149(3)  6.180  Cholesterol (TN), Cholesterol (JP17/NF)
D04961  3.169(4)  9.314  Wine (JP17), Wine (TN)
D06773  3.171(3)  4.694  Lonicera leaf and stem (JP17)
D01732  3.187(4)  4.581  Sodium sulfate, dried (TN), Anhydous sodium sulfate (JP17), Sodium sulfate, dried (JAN)
D02416  3.187(4)  4.581  ALterna GEL (TN), Aluminum hydroxide (USP), Dialume (TN), Dried aluminum hydroxide gel (JP17), Aluminum hydroxide, dried (USP)
D04172  3.187(4)  4.581  Tetucur (TN), Ferrous sulfate, dried (USP), Ferrous sulfate hydrate, Fero-gradumet (TN)
D05283  3.187(4)  4.581  Dried sodium carbonate (JP17), Sodium carbonate, dried, Sodium carbonate (NF)
D08708  3.187(4)  4.581  AM (TN), Licase (alpha Amylase), aluminum hydroxide, dried, magnesium carbonate, sodium bicarbonate and precipitated calcium carbonate, NS (TN)
D08793  3.187(4)  4.581  Feiba (TN), Human blood-coagulation factor eight inhibitor bypassing activity complex, dried
D08794  3.187(4)  4.581  Human blood-coagulation factor IX complex, dried, PPSB-HT (TN)
D08834  3.193(4)  5.363  Stomachic and digestive, Ohara (TN)
D08754  3.196(3)    -    L-Arginine and L-arginine hydrochloride, Argi-U (TN)
D02279  3.204(4)  8.208  L-Lysine hydrochloride (JP17), Lysine hydrochloride (USP), L-Lysine monohydrochloride
D02304  3.204(4)  8.208  L-Lysine, Lysine (USAN/INN)
D06469  3.204(4)  8.208  Lysine hydrochloride
D00019  3.214(4)  8.333  Methionine (USP), L-Methionine Z (TN), L-Methionine (JP17)
D04946  3.217(3)  5.707  Incremin (TN), Ferric pyrophosphate, soluble (JAN)
D08150  3.304(2)  3.237  Lufenuron (USP/INN), Program [veterinary] (TN)
D00085  3.237(3)  5.514  Insulin (JAN/USP)
D04546  3.237(3)  5.514  Insulin, dalanated (USAN)
D04547  3.237(3)  5.514  Humalog PEN (TN), Insulin, isophane (USP), Isophane insulin (aqueous suspension) (JAN), Isophane insulin human (genetical recombination) injectable aqueous suspension (JP17)
D04548  3.237(3)  4.158  Neutral insulin injection (INN), Insulin, neutral (USAN)
D00124  3.264(5) 11.105  Ephedrine (USP), Ephedrine (TN)
D01386  3.264(5) 11.105  Ephedrine hydrochloride (JP17/USP), Ephedrine hydrochloride (TN)
D04299  3.264(5)  9.125  Asthpyllin (TN), Diprophylline, ephedrine hydrochloride, papaverine hydrochloride, noscapine and diphenhydramine hydrochloride
D04307  3.264(5) 11.105  Asthmolysin D (TN), Proxyphylline, ephedrine hydrochloride and phenobarbital
D04338  3.264(5) 11.105  Dihydrocodeine phosphate, ephedrine hydrochloride and ammmonium chloride, Sekicode (TN)
D00075  3.271(3)  6.927  Androgel (TN), Axiron (TN), Androderm (TN), Striant (TN), Testosterone (JAN/USP), Testim (TN)
D01921  3.271(3)  6.927  Estradiol and testosterone, Bothermon (TN)
D04472  3.271(3)  6.129  Metharmon-F (TN), Pregnenolone, androstenedione, testosterone, estrone and dried thyroide
D06482  3.273(3)  6.129  Thyroid (USP), Thyradin (TN)
D11075  3.277(4)  9.818  Tussinonex (TN), Tussicaps (TN), Chlorpheniramine and hydrocodone
D11087  3.277(4)  9.818  Hydrocodone and guaifenesin, Flowtuss (TN)
D08045  3.277(4)    -    Hydrocodone (INN), Dihydrocodeinone
D08046  3.277(4)    -    Dicodid (TN), Hydrocodone hydrochloride, Dihydrocodeinone hydrochloride
D11076  3.277(4)    -    Tussigon (TN), Homatropine meylbromide and hydrocodone
D00093  3.277(3)  8.466  Powdered cellulose (JP17), Avicel PH (TN), Microcrystalline cellulose (JP17), Cellulose, microcrystalline (NF)
D03444  3.277(3)  7.887  Diluted opium powder (JP17), Opium (USP), Opium, powdered (JP17)
D06456  3.277(3)  8.466  Cellulose, oxidized regenerated (USP), Surgicel absorbable hemostat (TN)
D07622  3.277(3)  8.719  Carboxymethylcellulose, Croscarmellose (INN), Carmellose (JP17/NF/INN), Intrasite gel (TN), CMC
D03053  3.286(4)  6.699  Adhesive bandage, Bandage, adhesive (USP)
D06006  3.286(4)  6.699  Tape, adhesive (USP)
D00140  3.298(3)  8.719  Eryc (TN), Staticin (TN), Erythromycin (JP17/USP/INN), Erygel (TN), Akne-mycin (TN), Pce (TN), T-stat (TN)
D00423  3.298(4)  5.810  Copegus (TN), Rebetol (TN), Ribavirin (JP17/USP/INN), Ribasphere (TN), Virazole (TN)
D00933  3.298(3)  8.026  Calcium citrate tetrahydrate, Citracal (TN), Calcium citrate (USP)
D01781  3.298(3)  8.026  Trisodium citrate dihydrate, Sodium citrate (TN), Sodium citrate hydrate (JP17), Tricitrasol (TN)
D03265  3.298(3)  8.026  Tectlol (TN), Magnesium citrate (JAN/USP)
D03923  3.298(4)  6.180  Dusting powder, absorbable (USP)
D04305  3.298(4)  6.180  Gauze, absorbent
D05254  3.298(3)  8.541  Rice starch (TN), Starch, rice (NF), Rice starch (JP17)
D05578  3.298(3)  8.026  Potassium citrate (USP), Urocit-K (TN)
D05624  3.298(3)  8.026  Potassium citrate and sodium citrate hydrate, Uralyt (TN)
D05855  3.298(3)  8.026  Sodium citrate (USP)
D05932  3.298(4)  9.818  Zanosar (TN), Streptozocin (JAN/USAN/INN), Streptozotocin
D06428  3.298(4)  6.180  Gelatin film, absorbable, Gelfilm (TN)
D06429  3.298(4)  6.180  Gelfoam (TN), Gelatin sponge, absorbable
D11646  3.298(3)  8.719  Aktipak (TN), Benzoyl peroxide and erythromycin
D12020  3.298(4)  7.872  Garlic
D03263  3.298(4)    -    Sodium polystyrene sulfonate (JP17/USP), Kayexalate (TN), Kionex (TN)
D00034  3.300(2)  8.803  Adenine (JAN/USP), Leucon (TN)
D00352  3.303(3)    -    Lactulose (JP17/USP/INN), Chronulac (TN), Cephulac (TN)
D00016  3.304(2)  5.399  L-Serine (JP17), Serine (USP)
D00413  3.304(3)  8.803  Zidovudine (JP17/USP/INN), Retrovir (TN)
D00937  3.304(2)  6.984  Dibasic calcium phosphate hydrate (JP17), Dibasic calcium phosphate (TN), Calster (TN), Calcium phosphate, dihydrate, dibasic (USP)
D02050  3.304(2)  6.984  Potassium phosphate, monobasic (JAN/NF)
D02403  3.304(2)  6.984  Potassium phosphate, dibasic (JAN/USP), Mediject P (TN)
D03302  3.304(2)  6.984  CalStar (TN), Calcium phosphate, dibasic, Dibasic calcium phosphate, anhydrous (JAN)
D03303  3.304(2)  6.984  Calcium phosphate, tribasic (NF)
D04070  3.304(3)  6.901  Conjugated estrogens, Premarin (TN), Estrogens, conjugated (JAN/USP)
D04071  3.304(3)  7.062  Amnestrogen (TN), Menest (TN), Estrogens, esterified (USP)
D04400  3.304(2)  6.984  Anhydrous monobasic sodium phosphate, Sodium phosphate, monobasic (USP)
D04836  3.304(2)  6.984  Magnesium phosphate (USP), Magnesium phosphate pentahydrate
D05869  3.304(2)  6.984  Sodium phosphate, dibasic (USP)
D06389  3.304(2)  6.984  Sodium phosphate, dibasic, dodecahydrate, Phosphoric acid, disodiumsalt, dodecahydrate, Dibasic sodium phosphate hydrate (JP17)
D09000  3.304(2)  6.984  Sodium phosphate, tribasic (NF)
D00041  3.304(4)  9.026  Threonine (USP), L-Threonine (JP17)
D00045  3.304(4)  6.727  Adenocard (TN), Adenosine (JAN/USP), Adenoscan (TN)
D02326  3.304(4)  6.762  Elcys (TN), Cysteine hydrochloride (USP), L-Cysteine hydrochloride hydrate (JP17)
D08748  3.304(4)  6.762  Liver hydrolysate, cysteine hydrochloride, choline bitartrate, inositol and cyanocobalamin, Proheparum (TN)
D08530  3.304(4)    -    Streptokinase - streptodornase, Varidase (JAN), Varidase (TN)
D06333  3.316(5)    -    Xanthan gum (NF), Rhodigel (TN)
D01772  3.334(4)  9.496  Ether (JP17/USP), Diethyl ether
D06440  3.334(4)  6.291  Ether, anesthetic (JP17)
D05519  3.340(2)  5.436  Pituitary, posterior, Pituitrin (TN)
D05794  3.341(5)  7.045  Salts, rehydration (USP)
D07554  3.341(5)  7.045  Acetylcysteine Sodium (TN), Acetylcysteine sodium salt, L-Cysteine, N-acetyl-, sodium salt
D10802  3.341(5)  5.891  Carbonic-13C acid, calcium salt, Calcium carbonate (13C) (JAN)
D06457  3.343(5)  8.208  HCG (TN), A.P.L. (TN), Human chorionic gonadotrophin (JP17), Gonadotropin, chorionic (USP)
D00021  3.384(4)  8.719  L-Phenylalanine (JP17), Phenylalanine (USP/INN)
D00195  3.395(4)  8.719  Codeine hydrate, Codeine (USP)
D02101  3.395(4)  8.719  Codeine phosphate hydrate (JP17), Codeine (TN), Codeine phosphate (USP)
D07740  3.395(4)  8.719  Codeine hydrochloride, Bisoltus (TN)
D11852  3.395(4)  8.719  Codeine and promethazine
D11860  3.395(4)  6.727  Fiorinal with codeine (TN), Butalbital, aspirin, caffeine and codeine
D00090  3.410(3)  6.773  Thrombin (JP17/USP/INN), Factor IIa, Thrombostat (TN)
D00517  3.410(4)  5.847  Vaporole (TN), Aspiral (TN), Amyl nitrite (JP17/USP)
D02842  3.410(4)  7.872  Alum, ammonium (USP)
D08813  3.410(3)  6.773  Tachocomb (TN), Aprotinin, thrombin and human fibrinogen
D00012  3.431(4)  4.989  Alanine (USP), L-Alanine (JP17)
D00011  3.438(4)  8.466  Glycine (JP17/USP)
D00932  3.438(4)  5.555  Calcium carbonate, precipitated (JAN), Calcium carbonate (USP), Cal-sup (TN), Precipitated calcium carbonate (JP17)
D01446  3.438(4)  5.891  Magnesium carbonate (TN), Magnesium carbonate (JP17/USP)
D01780  3.438(4)  5.891  Sodium carbonate hydrate (JP17)
D01928  3.438(4)  9.125  Calcium linoleate (JAN)
D02038  3.438(4)  5.891  Potassium carbonate (JP17/USP), Racol (TN)
D02315  3.438(4)  9.818  Oleic acid (NF)
D03960  3.438(4)  5.891  Aspirin and dialuminate, Aspirin, aluminum glycinate and magnesium carbonate, Bufferin (TN)
D04480  3.438(4)  8.466  L-Glutamic acid, L-alanine and glycine, Paraprost (TN)
D04990  3.438(4)  8.466  Stronger neo minophagen C (TN), Monoammonium glycyrrhizinate, glycine and L-cysteine hydrochloride
D04993  3.438(4)  8.466  Monoammonium glycyrrhizinate, glycine and DL-methionine, Glycyron (TN)
D05424  3.438(4)  4.950  Albumin (TN), Human serum albumin (genetical recombination) (JAN), Human serum albumin
D07631  3.438(4)  5.891  Sulcain (TN), Ethyl piperidinoacetylaminobenzoate, magnesia alumina hydrate and precipitated calcium carbonate
D08061  3.438(4)  8.431  Eicosapentaenoic acid, Icosapent (INN)
D08704  3.438(4)  5.891  Cabagin-U (TN), Magnesium aluminometasilicate, methylmethionine sulfonium chloride, magnesium carbonate and precipitated calcium carbonate
D10259  3.438(4)  5.891  Famotidine, calcium carbonate and magnesium hydroxide, Pepcid complete (TN)
D00071  3.452(4)  9.125  Fibronectin (human plasma) (JAN)
D05527  3.452(4)  7.174  Platelet concentrate (USP), PC
D00931  3.452(4)  7.773  Phoslo (TN), Calcium acetate (USP)
D01154  3.452(4)  7.773  Potassium acetate (JAN/USP), Potassium acetate (TN)
D01779  3.452(4)  7.773  Sodium acetate (TN), Sodium acetate trihydrate, Sodium acetate hydrate (JP17), Sodium acetate (USP)
D02257  3.452(4)  7.773  Calcium acetate monohydrate, Calcium acetate (JAN)
D01892  3.458(5)  9.125  Vascepa (TN), Icosapent ethyl (USAN), Ethyl icosapentate (JP17), Epadel S (TN)
D00564  3.459(3)  7.045  Coumadin (TN), Jantoven (TN), Warfarin sodium (USP)
D01280  3.459(3)  7.045  Warfarin potassium (JP17), Athrombin-K (TN)
D06571  3.459(3)  8.719  Kybernin (TN), Antithrombin III human (USP), Antithrombin III (INN)
D08682  3.459(3)  4.496  Warfarin (INN), Warfarine, Choice (TN)
D02917  3.467(3)  8.272  Ammonia spirit, aromatic (USP)
D00060  3.470(5)  9.719  Dextran 1 (USP), Dextran (INN)
D00039  3.474(4)  9.818  Valine (USP), L-Valine (JP17)
D00083  3.474(3)  6.033  Nitrogen (TN), Nitrogen (JP17/NF)
D00111  3.474(4)  4.272  Lactic acid (JP17/USP), Lactate (TN)
D00936  3.474(4)  4.272  Prequist powder (TN), Calcium lactate (USP)
D02017  3.474(4)  9.026  Choline theophylline (JAN), Choline theophyllinate (INN), Oxtriphylline (USP), Theocolin (TN), Theophyline and choline
D02051  3.474(3)  8.621  Sensodyne (TN), Potassium nitrate (JAN/USP)
D02183  3.474(4)  4.272  Sodium lactate (JAN/USP), Mediject L (TN)
D02254  3.474(4)  4.272  Calcium lactate hydrate (JP17), Calcium lactate (TN), Calcium lactate pentahydrate
D02313  3.474(3)  8.621  Nitrate, Nitric acid (NF)
D07690  3.474(4)  9.026  Choline (DCF), Choline ion, Choline cation
D00014  3.487(5)  7.468  Glutathione (reduced type), Tathion (TN), Glutathione (JP17)
D00023  3.487(5)  5.707  Pastaron (TN), Carbamide, Urea (JP17/USP)
D00030  3.487(5)  8.719  L-Leucine (JP17), Leucine (USP)
D00031  3.487(5)  7.468  Glutathione (TN), Oxiglutatione (JAN/INN)
D01749  3.487(5)  5.707  Urea (13C) (JAN), Helicosol (TN), Urea C13 (USP)
D00107  3.499(3)  6.773  Protopic (TN), Tacrolimus (USP/INN), Tacrolimus hydrate (JP17), Prograf (TN)
D00371  3.499(3)  9.719  Theodur G (TN), Theophylline (JP17), Theo-24 (TN), Elixophyllin (TN), Quibron-t (TN), Theolair (TN), Uniphyl (TN)
D03336  3.499(3) 10.412  Sodium alginate (JAN/NF), Arcrane (TN), Ascophyllum
D05236  3.499(3)  9.125  Ointment, bland lubricating (USP)
D05237  3.499(3)  8.208  Hydrophilic cream (JP17), Hydrophilic ointment (TN), Hydrophilic ointment, Ointment, hydrophilic (USP)
D05238  3.499(3)  9.125  Ointment, rose water (USP)
D05240  3.499(3)  6.873  Ointment, yellow (USP)
D06103  3.499(3)  9.719  Theophylline (USP), Theophylline monohydrate, Accurbron (TN)
D06516  3.499(3)  6.873  Feen-a-mint gum (TN), Phenolphthalein, yellow
D08556  3.499(3)  6.773  Tacrolimus (INN), Prograf (TN)
D08977  3.499(3) 10.412  Potassium alginate (NF)
D05633  3.499(3)    -    Propylene carbonate (NF)
D08888  3.499(3)    -    Cetyl palmitate (NF)
D00384  3.499(3)    -    Triacetin (USP/INN), Enzactin (TN)
D07985  3.506(3)  8.621  Folate sodium, Folina (TN)
D08807  3.511(4)  7.515  Anti-human T-lymphocyte immunoglobulin, rabbit (JAN), Zetbulin (TN)
D09190  3.511(4)  7.515  Thymoglobuline (TN), Anti-human thymocyte immunoglobulin, rabbit (JAN)
D00068  3.518(4)  4.819  Ethyl alcohol, Dehydrated ethanol (TN), Alcohol (USP), Anhydrous ethanol (JP17), Dehydrated ethanol
D02799  3.518(4)  4.819  Alcohol, rubbing (USP), Alcolo (TN)
D04732  3.518(4)  4.819  Isopropanol and methylated alcohol, Alcohol (TN)
D10301  3.518(4)  4.819  Chlorhexidine gluconate and alcohol, Prevantics (TN)
D00150  3.519(2)  5.264  Angiotensin II (INN)
D02014  3.519(2)  5.264  Angiotensin II (human type) (JAN), Angiotensin II (USAN), Delivert (TN)
D04482  3.519(2)  6.384  Trivora (TN), Triphasil-21 (TN), Lo/ovral (TN), Ange (TN), Ethinylestradiol and levonorgestrel, Levora (TN), Seasonique (TN)
D06785  3.519(3)  9.125  Fangji (TN), Powdered sinomenium stem and rhizome (Non-JPS), Sinomenium stem (JP17)
D08729  3.519(3)  6.180  Salicylic acid and zinc oxide, Zinc and salicylic acid (TN)
D09138  3.519(3)  9.125  Asteris radix, Aster root (Non-JPS), Aster root and rhizome
D09297  3.519(3)  9.125  Ligusticum sinese rhizome (Non-JPS), Ligustici rhizoma, Ligusticum root and rhizome
D00192  3.519(3)    -    Phenylethyl alcohol (USP)
D06505  3.521(4)  4.154  Starch, topical (USP)
D03918  3.521(3)    -    Xigris (TN), Drotrecogin alfa (activated) (USAN), Drotrecogin alfa
D05865  3.536(4)  9.125  Sodium nitrite (USP), Sodium nitrite (TN)
D06177  3.545(4)  6.012  Tolbutamide sodium, sterile, Orinase diagnostic (TN)
D05253  3.568(4)  8.541  Wheat starch (JP17), Starch, wheat (NF)
D05297  3.568(4)  9.818  Starch, corn (NF), Corn starch (TN), Corn starch (JP17)
D06506  3.568(4)  9.818  Starch, pregelatinized (NF)
D06507  3.568(4)  9.818  Starch
D05872  3.572(4)  9.818  Sodium propionate hydrate, Sodium propionate (NF)
D08440  3.572(4)  9.818  Sodium propionate, Natriumpropionat [veterinary] (TN)
D09875  3.572(4)  9.818  Calcium propionate (NF)
D05286  3.577(5)  8.719  Simple syrup (JP17), Syrup (NF), Simple syrup (TN)
D00013  3.585(4)  9.125  L-Aspartic acid (JP17), Aspartic acid (USP/INN)
D00046  3.585(3)  8.621  Lactose, anhydrous (JAN/NF), Lactose (TN), Anhydrous lactose (JP17)
D00076  3.585(5)  8.026  Noradrenaline (JP17), Nor adrenalin (TN), Norepinephrine (INN)
D00753  3.585(2)  7.179  Rapamycin (TN), Sirolimus (JAN/USAN/INN), Rapamune (TN)
D01225  3.585(2)  9.125  Thiamine pyrophosphate, Cocarboxylase (JAN/INN), Coenzymate (TN)
D02026  3.585(4)  4.883  Magnesium L-aspartate (JAN), Magnesium aspartate
D03226  3.585(3)  8.621  Lactose (JP17), Lactose monohydrate (NF), Lactose (TN)
D04334  3.585(4) 10.007  Glucosamine (USAN/INN), D-Glucosamine
D04948  3.585(4)  4.883  Aspara K (TN), Potassium aspartate, L-Aspartate potassium, Potassium L-aspartate (JAN)
D04952  3.585(4)  4.883  Potassium aspartate and magnesium aspartate (JAN/USAN), Aspara (TN)
D08022  3.585(4) 10.007  Artrox (TN), Glucosamine hydrochloride
D09794  3.585(5)  8.026  (+/-)-Noradrenaline hydrochloride, Norepinephrine hydrochloride (JAN)
D00050  3.588(5)  8.719  Riboflavin (JP17/USP/INN), Bisulase (TN)
D04915  3.588(5)  8.719  Calcium pantothenate, riboflavin, pyridoxine hydrochloride and nicotinamide, Pancal (TN)
D04917  3.588(5)  8.719  Neurovitan (TN), Octotiamine, riboflavin, pyridoxine hydrochloride and cyanocobalamin
D04919  3.588(5)  8.719  Pyridoxal phosphate, fursultiamine hydrochloride, riboflavin and hydroxocobalamin acetate, Vitadan (TN)
D04937  3.588(5)  8.719  Bifuroxin (TN), Riboflavin and pyridoxine hydrochloride
D07859  3.588(5)  8.026  Ascorbic acid, thiamine nitrate, nicotinamide, calcium pantothenate, pyridoxine hydrochloride and riboflavin, Wasser-V (TN)
D08829  3.588(5)  8.719  Vitaneurin-capsule (TN), Hydroxocobalamin acetate, pyridoxal phosphate hydrate, fursultiamine hydrochloride and riboflavin
D08830  3.588(5)  8.026  Panvitan (TN), Retinol palmitate, thiamine nitrate, riboflavin, pyridoxine hydrochloride, cyanocobalamin, ascorbic acid, ergocalciferol, tocopherol acetate, calcium pantothenate, nicotinamide and folic acid
D08445  3.590(4) 11.105  Protamine (TN), Protamine hydrochloride (BAN)
D00114  3.592(3)  9.125  Fructose (JP17/USP), D-Fructose, Fructon (TN)
D00127  3.592(3) 11.105  Feldene (TN), Piroxicam (JP17/USP/INN)
D00401  3.592(3)  9.125  Tapazole (TN), Methimazole (USP), Thiamazole (JP17/INN)
D00483  3.592(3)  9.818  Propranolol hydrochloride (JP17/USP), Innopran XL (TN), Inderal (TN)
D00731  3.592(3)  8.466  Mint-o-mag (TN), Magnesia, (Milk of) (USP), Magnesium hydroxide (JAN/USP), Milmag (TN)
D03131  3.592(3)  9.125  Bismuth, [milk of] (USP)
D03138  3.592(3)  4.419  Blood cells, red (USP)
D03345  3.592(3) 11.105  beta-Galactosidase (Aspergillus) (JP17), Oryzatym (TN)
D03346  3.592(3) 11.105  beta-Galactosidase (Penicillium) (JP17)
D03935  3.592(3)  9.125  Galactose and palmitic acid, Levovist (TN)
D04291  3.592(3)  9.125  Galactose (NF), alpha-D-Galactose
D08443  3.592(3)  9.818  Propranolol (INN), Propranolol (TN)
D10274  3.592(3)  9.818  Propranolol hydrochloride and hydrochlorothiazide (TN), Inderide (TN), Propranolol hydrochloride and hydrochlorothiazide
D01817  3.592(3)    -    Iohexol (JP17/USP/INN), Omnipaque (TN)
D03395  3.592(3)    -    Carbomer 940 (NF), Carbopol 940 (TN), Polyacrylic acid
D00358  3.592(4)  8.908  Xylocaine (TN), Lidocaine (JP17/USP/INN), Dentipatch (TN)
D00433  3.592(4)  6.995  Sulfadiazine, silver (USP), Silvadene (TN), Sulfadiazine silver (JP17)
D00525  3.592(4) 10.007  Pilocarpine (JAN/USP), Ocusert pilo-20 (TN)
D01108  3.592(4)  6.857  Conclyte-Mg (TN), Magnesium sulfate (USP), Magnesium sulfate heptahydrate, Magnesium sulfate hydrate (JP17)
D01275  3.592(4)  9.125  Adriamycin (TN), Doxorubicin hydrochloride (JP17/USP), Doxil (TN), Adriacin (TN), Rubex (TN)
D01726  3.592(4)  6.857  Potassium sulfate (JP17/USAN)
D02086  3.592(4)  8.908  Xylocaine (TN), Dalcaine (TN), Lidocaine hydrochloride (JAN/USP)
D02200  3.592(4) 10.007  Salagen (TN), Pilopine HS (TN), Isopto carpine (TN), Pilocarpine hydrochloride (JP17/USP), Sanpilo (TN)
D03236  3.592(4)  5.541  Synthetic aluminum silicate (JP17), Aluminum silicate, synthetic (JAN), Silicamin (TN)
D03899  3.592(4)  7.872  Doxorubicin (USAN/INN), Adriblastina (TN), ADR
D03925  3.592(4)  8.397  Freeze-dried diphtheria antitoxin, equine (JP17), Freeze-dried diphtheria antitoxin (TN)
D04052  3.592(4)  7.620  Xylocaine (TN), Epinephrine and lidocaine hydrochloride, Adrenaline and lidocaine hydrochloride
D05187  3.592(4)  8.397  Freeze-dried gas gangrene antitoxin, equine (TN), Gas gangrene antitoxin, pentavalent, Gas gangrene antitoxin, equine (JP17)
D05317  3.592(4)  8.397  Freeze-dried habu antivenom, equine (JP17)
D05358  3.592(4)  5.541  Paraffin, synthetic (NF)
D05368  3.592(4)  8.397  Freeze-dried botulism antitoxin, equine (JP17)
D05372  3.592(4)  8.397  Freeze-dried mamushi antivenom, equine (JP17)
D05877  3.592(4)  6.857  Sodium sulfate (TN), Natrii Sulfus, Sodium sulfate hydrate (JP17), Sodium sulfate decahydrate, Natrium Sulfuricum, Sodium sulfate (USP)
D05963  3.592(4)  6.857  Sulfate, Sulfuric acid (NF)
D06513  3.592(4)  8.397  Freeze-dried tetanus antitoxin, equine (JP17)
D08681  3.592(4)  6.682  Wilfactin (TN), Vonvendi (TN), Von willebrand factor, Vonicog alfa (USAN), Vonicog alfa (genetical recombination) (JAN)
D08808  3.592(4)  8.397  Lymphoglobuline (TN), Anti-human thymocyte immunoglobulin, equine (JAN)
D09201  3.592(4)  6.857  Calcium sulfate (NF)
D11985  3.592(4)  6.857  Magnesium sulfate, anhydous sodium sulfate and potassium sulfate, Sulprep (TN)
D04843  3.592(4)    -    Malic acid (NF), Malate
D06298  3.599(2)  4.968  Vidarabine (JAN), Vidarabine anhydrous, ARA-A, Armes (TN)
D06532  3.602(3)  6.886  Travert 10% in plastic container (TN), Sugar, invert (USP)
D04361  3.603(6)  8.431  Gonadorelin acetate (USP), Luteinizing (TN)
D02176  3.608(4) 11.105  Levocarnitine (JAN/USP/INN), L-Carnitine, Carnitor (TN)
D00105  3.608(4)  7.872  Innofem (TN), Vivelle (TN), Estraderm (TN), Estring (TN), Divigel (TN), Estradiol (JAN/USP/INN), Estrace (TN), Vagifem (TN), Estrogel (TN), Estrasorb (TN), Climara (TN)
D04459  3.608(4)  7.872  Lutes (TN), Progesterone and estradiol
D07918  3.608(4)  7.872  Vagifem (TN), Estrasorb (TN), Estradiol hemihydrate
D09187  3.608(4)  7.872  Climara pro (TN), Estradiol and levonorgestrel, Wellnara (TN)
D09188  3.608(4)  7.872  Estradiol and norethindrone, Menoaid (TN), Activella (TN), Estradiol and norethindrone acetate (TN), Combipatch (TN)
D10856  3.608(4)  7.872  Nomegestrol acetate and estradiol, Zoely (TN)
D04958  3.609(5)    -    Livact (TN), L-Isoleucine, L-leucine and L-valine, L-Isoleucine, L-leucine and L-valine (JP17), Branched chain amino acids
D04744  3.610(4)  6.962  Hypoethanol (TN), Sodium thiosulfate and ethanol
D04761  3.610(4)  6.962  Camphor (TN), d-Camphor and ethanol
D06542  3.610(4)  6.962  Ethanol (JP17), Ethanol (TN)
D06620  3.610(4)  6.962  dl-Camphor and ethanol, Camphor (TN)
D04336  3.618(5)    -    Brocin codeine (TN), Cherry bark extract and codeine phosphate hydrate
D04371  3.623(3)  6.129  Soap, green (USP), Green soap
D00020  3.624(5)  8.272  L-Tryptophan (JP17), Tryptophan (USP/INN)
D00208  3.633(5)  9.719  MMC, Mitomycin C (JP17), Mitomycin (USP/INN), Mitomycin (TN), Muamycin (TN), Jelmyto (TN)
D02059  3.633(5) 11.105  Sodium bisulfite (TN), Sodium bisulfite (JP17)
D02309  3.633(4)  7.773  Methanol, Methyl alcohol (NF)
D03370  3.633(5)  7.515  Macrogol, Polyethylene glycol (NF), Sentry polyox WSR (TN), Polyethylene glycol 3350 (USP), Lutrol E (TN), Polyethylene oxide (NF), PEG
D05364  3.633(5)  8.719  Parathyroid hormone (human) (USAN), ALX 1-11, Parathyroid hormone (INN), Natpara (TN), PTH, Parathormone
D09153  3.633(5)  3.842  Elder
D06717  3.633(5)    -    Safflower (JP17), Safflower (TN)
D01612  3.633(5)    -    Laxoberal (TN), Laxoberon (TN), Sodium picosulfate hydrate (JP17)
D01994  3.633(5)    -    Mosapride citrate dihydrate, Gasmotin (TN), Mosapride citrate hydrate (JP17)
D00018  3.633(5)  8.026  Ascorbicap (TN), Ascorbic acid (JP17/USP/INN), ASCOR (TN), Ascoltin (TN)
D02293  3.633(5)  7.872  Calcium ascorbate (USP)
D02331  3.633(4)  8.026  Eprolin (TN), Vitamin E (USP)
D03969  3.633(5)  6.727  E.A.C (TN), Aspirin and ascorbic acid
D04899  3.633(5)  8.026  Thiamine chloride hydrochloride, riboflavin sodium phosphate and ascorbic acid
D04909  3.633(5)  8.026  Cinal (TN), Ascorbic acid and calcium pantothenate (JP17)
D04967  3.633(5)  8.026  Ophthalm K (TN), Carbazochrome, phytonadione and ascorbic acid
D05853  3.633(5)  7.872  Cevalin (TN), Sodium ascorbate (USP/INN)
D07575  3.633(5)  7.872  Calcium Ascorbate (TN), Ascorbic acid calcium salt
D07851  3.633(5)  8.026  C para (TN), Ascorbic acid, thiamine chloride hydrochloride, pyridoxine hydrochloride, riboflavin sodium phosphate, nicotinamide and panthenol
D08739  3.633(5)  8.026  Crystfan (TN), L-Cysteine and ascorbic acid
D08853  3.633(4)  9.818  Ammonium sulfate (NF)
D00619  3.637(4)  9.719  Vasolan (TN), Covera-hs (TN), Verapamil hydrochloride (JP17/USP), Calan (TN), Verelan (TN)
D02356  3.637(4)  9.719  Verapamil (USAN/INN)
D10282  3.637(4)  9.719  Tarka (TN), Trandolapril and verapamil hydrochloride
D03636  3.640(5)  9.818  L-Cystine (JP17), Cystine (USAN/INN)
D01598  3.640(5)    -    Calcium L-aspartate hydrate (JAN), Calcium L-aspartate, Aspara-CA (TN)
D06500  3.641(4)  7.620  Orange peel tincture (JP17), Osbeck (TN), Dried bitter orange peel (TN), Orange peel tincture, sweet (NF)
D00109  3.643(4)  6.727  Aspalon (JAN), Durlaza (TN), Easprin (TN), Acetylsalicylic acid, Aspirin (JP17/USP)
D02079  3.643(4)  6.727  Empirin compound (TN), Codein phosphate and aspirin
D02154  3.643(4)  6.727  Percodan-demi (TN), Codoxy (TN), Aspirin, oxycodone hydrochloride and oxycodone terephthalate
D02155  3.643(4)  6.727  Darvon compound-65 (TN), Aspirin, caffeine and propoxyphene hydrochloride
D07582  3.643(4)  6.727  Sodium acetylsalicylate, Aspirin sodium, Catalgine (TN)
D10513  3.643(4)  6.727  Complavin (TN), Clopidogrel and acetylsalicyclic acid, Clopidogrel sulfate and aspirin
D11176  3.643(4)  6.727  Percodan (TN), Oxycodone hydrochloride and aspirin
D11586  3.643(4)  6.727  Carisoprodol and aspirin
D11587  3.643(4)  6.727  Carisoprodol, aspirin and codeine phosphate
D11615  3.643(4)  6.727  Aggrenox (TN), Aspirin and dipyridamole
D11616  3.643(4)  6.727  Yosprala (TN), Aspirin and omeprazole
D11804  3.643(4)  6.727  Aspirin and vonoprazan, Cabpirin (TN)
D11849  3.643(4)  6.727  Norgesic forte (TN), Orphenadrine citrate, aspirin and caffeine
D11859  3.643(4)  6.727  Fiorinal (TN), Butalbital, aspirin and caffeine, Lanorinal (TN)
D00072  3.649(4) 11.105  Cytochrome c (JAN), Cytorest (TN)
D07749  3.656(4)  9.496  Cortisone (TN), Cortisone (INN)
D03031  3.661(4)  7.810  1-(4-Methylphenyl)ethylnicotinate, 1-(4-Methylphenyl)ethyl nicotinate (JAN)
D08050  4.166(2)  3.669  Polirreumin (TN), Hydroxychloroquine (INN)
D08079  3.672(4) 10.412  Inosital (TN), Inositol (NF)
D00035  3.683(4)  8.621  Proline (USP), L-Proline (JP17)
D00665  3.683(4)  9.818  Teldrin (TN), Chlorpheniramine maleate (JP17/USP), Chlor-trimeton (TN)
D01904  3.683(4)  8.208  Cefpiramide sodium (JP17/USAN), CPM, Suncefal (TN)
D02152  3.683(4)  7.214  Hydrocodone bitartrate and ibuprofen, Vicoprofen (TN)
D03411  3.683(4)  9.719  Marine colloids (TN), Carrageenan (NF)
D03428  3.683(4)  8.208  CPM, Cefpiramide (USP/INN)
D03742  3.683(4)  9.818  Dextromethorphan (USP)
D07398  3.683(4)  9.818  Clofeniramina (TN), Chlorpheniramine, Chlorphenamine (INN)
D11088  3.683(4)  9.818  Chlorpheniramine maleate, hydrocodone bitartrate and pseudoephedrine, Zutripro (TN)
D11647  3.683(4)  9.818  Brompheniramine, pseudoephedrine and dextromethorphan, Bromfed (TN)
D11854  3.683(4)  9.818  Promethazine dm (TN), Promethazine hydrochloride and dextromethorphan hydrobromide
D01561  3.683(4)    -    Potassium bitartrate (USP), Potassium hydrogen tartrate (JAN)
D03725  3.683(4)    -    Hydrocodone bitartrate hemipentahydrate, Dicodid (TN), Hydrocodone bitartrate (USP)
D10208  3.683(4)    -    Dextromethorphan hydrobromide and quinidine sulfate, Nuedexta (TN)
D00387  3.683(4)    -    Triazolam (JAN/USP/INN), Halcion (TN)
D00848  3.683(4)    -    Benylin DM (TN), Dextromethorphan hydrobromide hydrate (JP17), Dextromethorphan hydrobromide (USP)
D04340  3.683(4)    -    Dextromethorphan hydrobromide and potassium cresolsulfonate, Medicon (TN)
D04478  3.683(3)    -    Low substituted hydroxypropylcellulose (JP17), Lacrisert (TN), Hydroxypropylcellulose (JP17), Hydroxypropyl cellulose (USP)
D00096  3.683(3)    -    Sorbitol (NF), D-Sorbitol (JP17), Sorbitol solution (USP), Sorbitol 3% in plastic container (TN)
D02271  3.683(4)  8.110  Morphine hydrochloride trihydrate, Morphine hydrochloride hydrate (JP17), Anpec (TN)
D03876  3.683(4)  8.110  Morphine hydrochloride hydrate and atropine sulfate hydrate, Morphine and atropine (JP17), Morphine and atropine (TN)
D08233  3.683(4)  8.110  Morphine (BAN), Substitol (TN), Morfina Dosa (TN)
D03383  3.683(6)    -    Murine ear drops (TN), Carbamide peroxide (USP)
D01711  3.688(5)  8.014  Sodium hypochlorite (JAN/USP), Texant (TN)
D01727  3.688(5)  8.014  Calcium hypochlorite, Chlorinated lime (JP17)
D02493  3.688(5)  8.208  Tilmicosin phosphate (USAN), TP
D08651  3.688(5)    -    Sodium dichloroisocyanurate, Klorsept (TN), Troclosene sodium
D10527  3.700(5)  8.803  Acetylsalicylic acid and lansoprazole, Takelda (TN)
D00037  3.703(4)  9.719  Anhydrous citric acid (JP17), Citric acid anhydrous (JAN), Citric acid, anhydrous (USP)
D01222  3.703(4) 10.007  Citric acid (TN), Citric acid hydrate (JP17), Citric acid monohydrate (USP)
D02120  3.703(4)  8.026  Citric acid monohydrate and potassium bicarbonate, K-lyte (TN)
D05453  3.703(4)  8.431  PCP, Phencyclidine hydrochloride (USAN)
D05229  3.703(4)    -    Polyoxysthylene mono(octylphenyl) ether, Octoxinol (INN), Octoxynol 9 (NF), Triton X-100 (TN)
D02296  3.703(4)    -    Isopropyl myristate (NF), Estergel (TN)
D02305  3.710(4)  7.045  Rosoxacin (USAN/INN), Roxadyl (TN), ROS
D02490  3.710(4)  7.277  Tylan (TN), Tylosin (USP/INN), TS
D00015  3.723(4)  8.026  Endari (TN), Nutrestore (TN), Glutamine (USP), Levoglutamide, L-Glutamine (JP17)
D05983  3.725(4)  8.208  Suture, adsorbable surgical (USP)
D06518  3.725(4)  8.208  Suture, nonabsorbable surgical (USP), Ethibond (TN)
D02275  3.736(3)  9.818  Succin (TN), Suxamethonium chloride hydrate (JP17), Suxamethonium chloride dihydrate
D10914  3.736(3)  9.818  Calcium succinate monohydrate, Calcium succinate (USP)
D04182  3.739(4)  5.030  Ibrin (TN), Fibrinogen I 125 (USAN), Fibrinogen (125I) (INN)
D00065  3.742(3)  9.818  L-Isoleucine (JP17), Isoleucine (USP)
D03928  3.746(5)    -    Resovist (TN), Ferucarbotran (JAN/USAN)
D03365  3.747(4)  6.901  Habitrol (TN), Nicotine (USP)
D00033  3.752(5)  8.014  Phenol, liquefied, Phenol (TN), Phenol for disinfection (JP17), Liquefied phenol (TN), Phenol for disinfection (TN), Paoscle (TN), Liquefied phenol (JP17), Phenol (JP17/USP)
D03391  3.752(5)  8.014  Carbol and fuchsin, Phenol and fuchsin
D04802  3.752(5)  8.014  Phenol and zinc oxide liniment (TN), Phenol and zinc oxide liniment (JP17)
D04840  3.752(5)  8.014  Dental phenol with camphor (JP17), Phenol and camphor, Camphenic (TN)
D04850  3.752(5)  8.014  Phenol, thymol and dl-menthol, Phenol with thymol (TN)
D05455  3.752(5)  8.014  Phenolate sodium (USAN)
D06534  3.752(5)  8.014  Phenolated water for disinfection (TN), Phenolated water for disinfection (JP17), Phenol water (TN), Phenolated water (JP17), Phenol hydrate
D00584  3.757(4)  9.818  Adrucil (TN), Carac (TN), Fluoroplex (TN), Fluorouracil (JP17/USP/INN), 5-FU (TN)
D00146  3.758(3)  9.496  Acthar (TN), ACTH (TN), Corticotropin (USP/INN)
D07561  3.759(5)  9.818  beta-Alanine, Abufene (TN)
D02747  3.768(5)    -    Peginterferon alfa-2a (USAN/INN), Peginterferon alfa-2a (genetical recombination) (JAN), Pegasys (TN)
D03341  3.769(4)  9.026  Urokinase (JP17/USAN/INN), Abbokinase (TN)
D04812  3.772(5)  8.466  Sucrose and povidone iodine, Sorenurse (TN)
D04968  3.775(5)  6.873  Yellow wax (TN), Yellow beewax (JP17), Wax, Yellow (NF)
D02171  3.783(6)  9.818  Pursennid (TN), Sennosides (USP)
D10248  3.783(6)  9.818  Docusate sodium and sennosides
D03658  3.787(6)  8.908  Dasatinib (JAN/INN)
D06414  3.787(6)  8.908  Dasatinib hydrate (JAN), Dasatinib (USAN), Sprycel (TN)
D03213  3.788(4)  8.208  Eliquis (TN), Apixaban (JAN/USAN/INN)
D03353  3.788(4)  9.160  Dalteparin sodium (JAN/USP/INN), Fragmin (TN), Ardeparin sodium
D07086  3.788(4)  8.161  Rivaroxaban (JAN/USAN/INN), Xarelto (TN)
D08873  3.788(4) 11.105  Betrixaban (USAN), Bevyxxa (TN)
D09707  3.788(4)  9.496  Dabigatran (USAN/INN)
D09710  3.788(4) 10.412  Edoxaban (USAN/INN)
D09676  3.788(4)    -    Tecarfarin (USAN/INN)
D09677  3.788(4)    -    Tecarfarin sodium (USAN)
D06142  3.788(4)    -    Tifacogin (USAN/INN)
D03200  3.788(3)    -    1-Butanol, Butyl alcohol (NF)
D00554  3.791(5) 11.105  Ethinylestradiol (JP17/INN), Estinyl (TN), Ethinyl estradiol (USP)
D04483  3.791(5) 11.105  Norethindrone and ethinyl estradiol, Ethinylestradiol and norethisterone, Synphase T28 (TN)
D10590  3.791(5) 11.105  Trinessa (TN), Mononessa (TN), Norgestimate and ethinyl estradiol
D10839  3.791(5) 11.105  Xulane (TN), Norelgestromin and ethinyl estradiol, Evra (TN)
D01091  3.808(4) 11.105  Sulfactol (TN), Sodium thiosulfate (USP), Detoxol (TN), Sodium thiosulfate hydrate (JP17)
D04351  3.808(3)  9.818  Biolactis (TN), Lactobacillus casei
D09001  3.808(4)  9.160  Sodium sulfide (USP), Sodium sulfide nonahydrate
D09002  3.808(4)    -    Sodium sulfite (NF), Dried sodium sulfite (JP17)
D04271  3.808(3)    -    D-Mannitol and D-sorbitol, Mannitol S (TN)
D03484  3.809(4)  6.984  Chymotrypsin (JAN/USP/INN), Catarase (TN)
D00938  3.810(3)  7.522  Tricalcium phosphate, Posture (TN)
D05332  3.810(3)  8.208  Petrolatum, hydrophilic, Hydrophilic petrolatum (JP17)
D00251  3.815(4)  9.026  Capoten (TN), Captopril (JP17/USP/INN), Apopril (TN)
D06544  3.815(3)  5.885  Corticotropin, repository (USP), Cortigel (TN)
D08820  3.815(3)  8.333  Fresh frozen plasma -leukocytes reduced (FFP-LR), Fresh frozen plasma (FFP) (TN), Fresh-frozen human plasma
D10276  3.815(4)  9.026  Capozide (TN), Captopril and hydrochlorothiazide
D10845  3.815(3)  9.818  Ruconest (TN), Conestat alfa (INN)
D02436  3.815(4)    -    Sulfadimidine (INN), Sulfamezathine (TN), Sulfamethazine (USP)
D02456  3.815(4)    -    RVPaba lipstick (TN), p-Aminobenzoic acid, Aminobenzoic acid (USP)
D02906  3.815(4)    -    Potaba (TN), Aminobenzoate potassium (USP)
D02907  3.815(4)    -    Sodium aminobenzoate, Aminobenzoate sodium (USP)
D00160  3.815(3)    -    Amicar (TN), Epsilon-Aminocaproic acid (JAN), Aminocaproic acid (USP/INN)
D00289  3.815(3)    -    Danazol (JP17/USP/INN), Danocrine (TN)
D00156  3.815(4)  9.719  Enoxolone (INN), Hidermart (TN), Glycyrrhetinic acid (JAN)
D00244  3.815(4)  9.125  Celestone (TN), Rinderon (TN), Betamethasone (JP17/USP/INN)
D00857  3.815(4)  9.818  Benzalkonium chloride (JP17/NF/INN), Zephiran chloride (TN)
D02769  3.815(4) 10.412  Adenyl (TN), Adenosine phosphate (USAN/INN)
D04425  3.815(4)  7.620  Despa (TN), Diphenhydramine salicylate, hydrocortisone acetate, benzalkonium chloride and chlorhexidine hydrochloride
D04447  3.815(4)  9.125  Celestamine (TN), Betamethasone and d-chlorpheniramine maleate
D08040  3.815(4)  8.110  Histamine (DCF), Histaminum (TN)
D08092  3.815(4)  5.775  Isoxsuprine (INN), Dilator (TN)
D08265  3.815(4) 11.105  Hydroquinine hydrobromide, inhibin (TN)
D08716  3.815(4)  9.719  Stick zenol A (TN), dl-Camphor, l-menthol, glycyrrhetinic acid and methyl salicylate
D08811  3.815(4)  9.818  Lades A (TN), Dicyclohexyl ammonium nitrite and benzalkonium chloride
D10300  3.815(4)  9.125  Lotrisone (TN), Clotrimazole and betamethasone
D06987  3.815(4)    -    Shoseiryuto extract (JP17), Shoseiryuto
D00972  3.815(4)    -    Celestone (TN), Betamethasone sodium phosphate (JP17/USP)
D04446  3.815(4)    -    Celestone soluspan (TN), Rinderon (TN), Betamethasone acetate and betamethasone sodium phosphate
D00004  3.823(5)  7.045  Carbon dioxide (TN), Carbon dioxide (JP17/USP)
D00566  3.825(5) 10.007  Sodium salicylate (JP17/USP), Salsonin (TN)
D00827  3.825(5) 10.007  Magnesium salicylate (USP), Magnesium salicylate tetrahydrate, Magan (TN)
D04011  3.825(5)  9.818  Chondroitin sulfate sodium and sodium salicylate, Kashowadol (TN)
D04016  3.825(5) 10.007  Camphor and sodium salicylate, Camphorin (TN)
D04022  3.825(5)  9.496  Sodium salicylate, dibucaine hydrocholoride and calcium bromide, Neo vitacain (TN)
D01203  3.826(6)  8.026  Meylon (TN), Sodium bicarbonate (JP17/USP), Neut (TN)
D02077  3.826(6)  8.026  Potassium bicarbonate (USP), K-vescent (TN), Potassium hydrogencarbonate
D03901  3.826(6)  8.026  Gastrast (TN), Sodium bicarbonate and tartaric acid
D04344  3.826(6)  8.026  Azulene sulfonate sodium and sodium bicarbonate, Hachiazule (TN)
D04391  3.826(6)  8.026  Swertia and sodium bicarbonate (JP17), Swertia sodium bicarbonate (TN)
D04402  3.826(6)  8.026  New Lecicarbon (TN), Sodium bicarbonate and anhydrous monobasic sodium phosphate
D06814  3.826(6)  8.026  Sodium bicarbonate and bitter tincture (JP17)
D08706  3.826(6)  8.026  Biasan (TN), l-Menthol, cinnamon bark, gentian, zanthoxylum fruit, ginger and sodium bicarbonate
D08707  3.826(6)  8.026  Bellsan (TN), l-Menthol, gentian, scopolia extract powder and sodium bicarbonate
D08709  3.826(6)  8.026  Picrasma wood and sodium bicarbonate
D11706  3.826(6)  8.026  Zegerid (TN), Omeprazole and sodium bicarbonate
D00098  3.828(4) 11.105  Camphor (USP), dl-Camphor (JP17)
D05357  3.828(4) 11.105  Parachlorophenol, camphorated
D00027  3.843(2)  9.160  Uracil (JAN/USAN)
D02131  3.843(2)  9.160  Uftoral (TN), Tegafur and uracil
D09776  3.843(2)  9.160  Uracil (2-13C) (JAN)
D00089  3.843(5)  9.026  Syntocinon (TN), Oxytocin (TN), Pitocin (TN), Oxytocin (JP17/USP/INN)
D00485  3.845(5) 11.105  Sudafed (TN), Pseudoephedrine hydrochloride (USP)
D08449  3.845(5) 11.105  Pseudoephedrine (INN), Acunaso (TN), Neodurasina (TN)
D10252  3.845(5) 11.105  Fexofenadine hydrochloride and pseudoephedrine hydrochloride, Allegra-d (TN)
D00437  3.849(4)  9.719  Nifedipine (JP17/USP/INN), Afeditab CR (TN), Procardia (TN), Adalat (TN)
D04962  3.856(5)    -    Intralipid (TN), Soybean oil (JP17/USP), Intrafat (TN)
D02343  3.856(5)    -    Carboprost (USAN/INN)
D00113  3.856(5)  9.818  Atropine (USP), Atropen (TN)
D00147  3.856(5) 11.105  Hyoscyamine (USP)
D00301  3.856(5)  9.818  Lomotil (TN), Atropine sulfate and diphenoxylate hydrochloride, Diphenoxylate and atropine
D03863  3.856(5)  9.818  Opium alkaloids and atropine (JP17), Opiato (TN), Opium alkaloids hydrochloride and atropine sulfate hydrate
D03872  3.856(5)  9.818  Compound oxycodone and atropine (JP17), Pavinal atropine (TN)
D00138  3.856(5)    -    Transderm scop (TN), Scopolamine (INN)
D03866  3.856(5)    -    Opisco (TN), Opium alkaloids hydrochloride and scopolamine hydrobromide hydrate, Weak opium alkaloids and scopolamine (JP17), Opium alkaloids and scopolamine (JP17)
D02844  3.857(4)  3.964  Aluminum carbonate, basic (USAN), Basaljel (TN)
D03300  3.857(4)  9.719  Calcium pantothenate, racemic (USP)
D03678  3.857(4)  3.964  Fuchsin, basic (USP)
D04519  3.857(5)  6.291  Incyclinide (USAN), Metastat (TN)
D00189  3.858(4) 11.105  Diol (TN), Pregnanediol (JAN)
D00194  3.862(4)  9.719  Limonene, d-Limonene (JAN)
D00116  3.873(5)  8.708  Glucagon (TN), Glucagon (JAN/USP/INN), Glucagon (genetical recombination) (JAN)
D00336  3.873(5)  9.818  Micronase (TN), Glyburide (USP), Glibenclamide (JP17/INN), Glynase (TN), Diabeta (TN)
D00443  3.873(5)  8.719  Spironolactone (JP17/USP/INN), Aldactone (TN)
D02118  3.873(5)  8.708  Glucagon hydrochloride, Glucagon (TN), Glucagon monohydrochloride, Glucagen (TN)
D07431  3.873(5) 11.105  Somatostatin (INN)
D10266  3.873(5)  6.843  Glyburide and metformin hydrochloride, Glibenclamide and metformin hydrochloride, Glucovance (TN)
D10270  3.873(5)  8.719  Aldactazide (TN), Spironolactone and hydrochlorothiazide
D10528  3.873(5)  5.307  Aldosterone (INN)
D09596  3.873(5)    -    Collagenase santyl (TN), Xiaflex (TN), Collagenase clostridium histolyticum (USAN), Collagenase (Clostridium histolyticum) (JAN)
D01067  3.873(4)  9.719  Polysorbate 80 (JP17/NF/INN), Tween 80 (TN)
D01968  3.873(4)  9.818  Zoledronic acid (USAN), Reclast (TN), Zometa (TN), Zoledronic acid hydrate (JAN)
D06378  3.873(4)  9.719  Zoledronate disodium (USAN), Zoledronate disodium hydrate
D08689  3.873(4)  9.818  Reclast (TN), Zoledronic acid (INN), Zometa (TN)
D08752  3.873(4)  8.431  Elase (TN), DNase and fibrinolysin
D05363  3.876(6)    -    Pullulan (JP17)
D00108  3.880(5)  7.011  Iodine (JP17/USP), Cadex (TN)
D00563  3.880(5)  7.045  Reflex (TN), Mirtazapine (JAN/USP/INN), Remeron (TN)
D00752  3.880(5)  7.179  Mycophenolate mofetil (JAN/USP), Cellcept (TN)
D01016  3.880(5) 11.105  Potassium iodide (JP17/USP), Thyroblock (TN)
D02265  3.880(5) 10.412  Protoporphyrin disodium (JAN), Prolmon (TN)
D03283  3.880(5)  7.011  Cadexomer iodine (USAN), Iodine and cadexomer, Iodosorb (TN)
D03670  3.880(5) 11.105  Deferoxamine (USAN)
D04838  3.880(5)  7.011  Iodine zinc iodide for Kantop (TN), Zinc iodide and iodine
D04874  3.880(5) 11.105  Sodium iodide (JP17/USP)
D04875  3.880(5)  7.011  Zinc sulfate, iodine, sodium iodine and glycerin, Neoglycerol (TN)
D04956  3.880(5)  9.818  Ferric chloride, manganese chloride, zinc sulfate, cupric sulfate and potassium iodide, Elemenmic (TN)
D04957  3.880(5)  9.818  Ferric chloride, zinc sulfate, cupric sulfate and potassium iodide, Elemate (TN)
D05094  3.880(5)  7.179  Mycophenolate mofetil hydrochloride (USAN), Cellcept (TN)
D05095  3.880(5)  6.773  Mycophenolate sodium (USP), Myfortic (TN)
D05096  3.880(5)  8.621  Mycophenolic acid (TN), Mycophenolic acid (USAN/INN)
D05328  3.880(5) 11.105  Iodocapsule 123 (TN), Sodium iodide (123I) (JP17), Sodium iodide I 123 (USP)
D05863  3.880(5) 11.105  Sodium iodide (125I) (INN), Sodium iodide I 125 (USAN), Iodotope I-125 (TN)
D07709  3.880(5)  7.011  Polyvinyl alcohol and iodine, PA iodo (TN)
D07780  3.880(5) 11.105  Desferal (TN), Deferoxamine hydrochloride (USAN)
D08403  3.880(5)  6.682  Nabumeton A (TN), Potassium
D05581  3.880(5)    -    Potassium metabisulfite (NF)
D02054  3.880(5)    -    Sodium metabisulfite (NF), Sodium pyrosulfite (JP17)
D00062  3.897(5) 10.007  Osmitrol (TN), Tobrex (TN), D-Mannitol (JP17), Mannitol (USP), Bronchitol (TN)
D06902  3.897(5)  8.541  Tritici fructus, Wheat (Non-JPS), Shobaku
D00006  3.897(5)    -    Hipyridoxin (TN), Pyridoxal phosphate hydrate (JP17), Pyridoxal phosphate monohydrate
D03281  3.897(5)    -    Pyridoxal calcium phosphate (JAN), Aderoxal (TN)
D04902  3.897(5)    -    Flavin adenine dinucleotide sodium and pyridoxal phosphate, Ribomin S (TN)
D04922  3.897(5)    -    Vitaneurin (TN), Fursultiamine, pyridoxal phosphate and hydroxocobalamin acetate
D08336  3.905(5)  8.026  Peplomycin (INN), PEP
D08133  3.917(4)  8.541  Lithium, Normothymin-E (TN)
D00212  3.923(4)  8.208  Vancomycin (USP), Vancocin (TN)
D00926  3.923(4)  8.208  Vancomycin hydrochloride (JP17/USP), Vancoled (TN), Vancocin (TN)
D06543  3.931(3)  7.620  Vitamin A (USP), Retinol, Aquasol A (TN), Vitamin A1
D02301  3.940(5)  8.110  Aqualinic (TN), Oxolinic acid (USAN/INN), OA
D00047  3.941(5) 10.412  Taurine (JP17/USP/INN), Taurine (TN), Aminoethylsulfonic acid (JAN)
D02011  3.942(5)  9.818  FAD sodium, Flavin adenine dinucleotide sodium (JP17), FAD (TN)
D07633  3.942(5)  9.818  Mucofadin (TN), Chondroitin sulfate sodium and FAD sodium, Mucotear (TN)
D10530  3.942(5)  9.818  Sodium selenite (JAN), Aselend (TN)
D01136  3.946(4)  7.515  Transamin (TN), Tranexamic acid (JP17/USP/INN), Rikavarin (TN), Cyklokapron (TN)
D04296  3.951(4)  4.841  Asthma (TN), Diprophylline, methoxyphenamine hydrochloride, noscapine and chlorpheniramine maleate
D01390  3.955(4)    -    Proternol-S (TN), dl-Isoprenaline hydrochloride (JAN), Isoproterenol hydrochloride (USP), Isuprel (TN)
D00186  3.955(5)  8.431  Cipro (TN), Ciprofloxacin (JP17/USP/INN), Otiprio (TN)
D02216  3.955(5)  8.431  Cipro (TN), Ciloxan (TN), Ciprofloxacin hydrochloride (USP), Ciprofloxacin hydrochloride hydrate (JP17), Proquin XR (TN)
D10296  3.955(5)  5.154  Ciprodex (TN), Ciprofloxacin hydrochloride and dexamethasone
D10822  3.955(5)  8.431  Ciprofloxacin hydrate (JAN), Ciprofloxacin hemiheptahydrate
D11089  3.955(5)  8.431  Ciprofloxacin hydrochloride and fluocinolone acetonide, Otovel (TN)
D11582  3.955(5)  7.515  Cipro hc (TN), Ciprofloxacin hydrochloride and hydrocortisone
D02908  3.956(3)    -    Ameluz (TN), Levulan (TN), Gleolan (TN), Aminolevulinic acid hydrochloride (JAN/USAN), 5-Aminolevulinic acid hydrochloride
D07567  3.956(3)    -    Aminolevulinic acid
D02332  3.959(5) 10.412  dl-alpha-Tocopherol, Tocopherol (JP17)
D04805  3.959(5)  7.620  Tocopherol and vitamin A, Juvela (TN)
D00361  3.966(4)  9.125  Liotrix (USP), Levothyroxine sodium and liothyronine sodium, Thyrolar (TN)
D01010  3.966(4)  9.125  Levothroid (TN), Levothyroxine sodium (USP), Levoxyl (TN), Synthroid (TN), Levothyroxine sodium hydrate (JP17)
D01011  3.966(4)  9.818  Liothyronine sodium (JP17/USP), Triostat (TN), Cytomel (TN)
D06118  3.966(4)  9.496  Thyroglobulin (USAN/INN), Proloid (TN)
D08125  3.966(4)  9.125  Forthyron (TN), Levothyroxine (BAN)
D08128  3.966(4)  9.818  Liothyronine (INN), Thyrolar (TN)
D08129  3.966(4)  9.818  Liothyronine hydrochloride, Thyrotradin (TN)
D11113  3.966(4)  9.125  Levothyroxine sodium (INN), Levothyroxine sodium anhydrous
D03311  3.968(4)  9.496  Olive oil (JP17/NF), Olive oil (TN)
D08778  3.968(4)  9.125  Freeze-dried inactivated tissue cluture hepatitis A vaccine, Aimmugen (TN), Freeze-dried, inactivated hepatitis A vaccine (JAN)
D00094  3.969(5)  9.818  Tretinoin (JAN/USP/INN), Retin A (TN), Renova (TN), Tretinoin (TN), Avita (TN)
D11084  3.969(5)  9.818  Ziana (TN), Clindamycin phosphate and tretinoin, Veltin (TN)
D04492  3.969(4)    -    Icatibant acetate (JAN/USAN), Firazyr (TN)
D00777  3.974(5)  8.272  Symmetrel (TN), Amantadine hydrochloride (JP17/USP), Osmolex er (TN), Gocovri (TN)
D01559  3.974(5)  7.253  L-Cysteine, ethyl ester, hydrochloride, Ethyl L-cysteine hydrochloride (JP17), Daiace (TN)
D07441  3.974(5)  8.272  Amantadine (INN)
D07769  3.974(5)  9.125  Cytidine, Posilent (TN)
D11472  3.974(5)  4.121  Veklury (TN), Remdesivir (JAN/USAN)
D04348  3.974(4)  8.708  Organidin (TN), Glycerol, iodinated (USAN)
D04800  3.979(6)  9.125  Heparinoid, adrenal extract and salicylic acid, Amel S (TN)
D08740  3.979(6)    -    Yellow beewax, almond oil and salicylic acid, Bone wax (TN)
D00097  3.979(6)    -    Salicylic acid (JP17/USP), Salicylic acid (TN)
D07726  3.989(4) 11.105  Clomiphene, Clomifene (INN), Clomifene (TN)
D00078  3.991(4)  9.818  Propylene glycol (JP17/USP), Propylene glycol (TN)
D00088  3.991(4)  7.515  Anusol HC (TN), Acticort (TN), Cortef (TN), HC, Plenadren (TN), Colocort (TN), Hydrocortisone (JP17/USP/INN), Hytone (TN)
D00201  3.991(4)  9.026  Sumycin (TN), Tetracycline (JAN/USP/INN)
D00235  3.991(4) 11.105  Tenormin (TN), Atenolol (JP17/USP/INN)
D00726  3.991(4) 10.412  Elieten (TN), Terperan (TN), Metoclopramide (JP17/INN)
D01471  3.991(4) 11.105  (S)-Atenolol, Esatenolol (JAN/INN)
D01724  3.991(4)  6.682  Alum, potassium (USP), Aluminum potassium sulfate (TN), Aluminum potassium sulfate hydrate (JP17)
D02122  3.991(4)  9.026  Bristacycline (TN), Tetracycline hydrochloride (JP17/USP)
D02213  3.991(4) 10.412  Reglan (TN), Primperan (TN), Metoclopramide hydrochloride (JAN), Metoclopramide dihydrochloride monohydrate
D02311  3.991(4)  9.719  2-Propanone, Acetone (TN), Acetone (NF)
D04662  3.991(4) 11.105  Lanolin (USP), Lanolin (TN), Purified lanolin (JP17)
D04775  3.991(4)  9.026  Tetra cortisone (TN), Tetracycline hydrochloride and hydrocortisone acetate
D04865  3.991(4)  9.026  Tetracycline presteron (TN), Tetracycline hydrochloride and Epidihydrocholesterin
D05008  3.991(4) 10.412  Clopra (TN), Reglan (TN), Metoclopramide hydrochloride (USP), Metoclopramide hydrochloride monohydrate
D05838  3.991(4)  8.621  Silica, dental-type (NF)
D06521  3.991(4) 11.105  Silicon dioxide (NF), Silica gel, Light anhydrous silicic acid (JP17)
D06522  3.991(4)  7.420  Silica, Silicon dioxide, colloidal (NF)
D08398  3.991(4) 11.105  Policosanol, Ateromixol (TN), PPG (TN)
D10245  3.991(4)  9.026  Bismuth subcitrate, tetracycline and metronidazole, Pylera (TN), Helidac (TN)
D10592  3.991(4) 11.105  Tenoretic (TN), Atenolol and chlorthalidone
D08368  3.991(4)    -    Fansia (TN), Phenylpropanolamine (INN)
D00245  3.991(4)    -    Dulcolax (TN), Bisacodyl (JP17/USP/INN)
D01224  3.991(4)    -    Phenylpropanolamine hydrochloride (JAN/USP)
D00542  3.991(4)    -    Halothane (JP17/USP/INN), Fluothane (TN)
D08518  3.991(4)    -    beta-Sitosterol, Harzol (TN)
D02189  3.991(4)    -    Magnesium stearate (JP17/NF)
D03093  3.991(4)    -    Bepio (TN), Benzoyl peroxide (JAN/USP)
D03310  3.991(4)    -    Calcium stearate (JP17/NF)
D03573  3.991(4)    -    Coal tar (USP), AquaTar (TN)
D04632  3.991(4)    -    Isopropyl palmitate (NF)
D05875  3.991(4)    -    Sodium stearate (NF)
D10602  3.991(4)    -    Duac (TN), Onexton (TN), Clindamycin phosphate and benzoyl peroxide
D10681  3.991(4)    -    Butyl stearate (NF), N-Butyl stearate
D10792  3.991(4)    -    Epiduo (TN), Adapalene and benzoyl peroxide
D05170  3.991(4)    -    Niridazole (USAN/INN)
D00029  3.998(4)  8.719  Bioepiderm (TN), Biotin (JP17/USP/INN)
D00067  3.998(4) 11.105  Estrone (TN), Estrone (JAN/USP/INN)
D00095  3.998(4)  7.620  Epinephrine (USP/INN), Epipen (TN), Adrenaline (JP17), Adrenalin (TN), Auvi-q (TN)
D00945  3.998(4)  8.431  Pioglitazone hydrochloride (JP17/USP), Actos (TN)
D01261  3.998(4)  7.847  Fluorescite (TN), Fluorescein (JAN/USP)
D01481  3.998(4)  9.818  Dihydrocodeine phosphate (JP17), Dihydro (TN)
D02024  3.998(4)  7.847  Fluorescein sodium (JP17/USP), Floures (TN)
D02740  3.998(4)  8.908  Lidocaine and propitocaine, Emla (TN), Lidocaine and prilocaine, Lidocaine and prilocaine (TN), Oraqix (TN)
D03139  3.998(3)  7.098  Blood group specific substances A, B, and AB
D04688  3.998(4)  8.908  Borraza-G (TN), Tribenoside and lidocaine
D04692  3.998(4)  8.908  Neriproct (TN), Diflucortolone valerate and lidocaine
D04813  3.998(4)  8.908  Lidocaine hydrochloride and epinephrine bitartrate, Ora (TN), Lidosite (TN)
D04881  3.998(4)  8.803  Aluminum chloride, cetylpyridinium chloride and Lidocaine, Dental TDZ (TN)
D05070  3.998(4) 10.412  Mono- and di-glycerides (NF), Myverol (TN)
D05071  3.998(4) 10.412  Mono- and di-acetylated monoglycerides, Myvacet (TN)
D07742  3.998(4)  8.908  Ethyl aminobenzoate, lidocaine and bismuth subgallate, Helmitin S (TN)
D08127  3.998(4)  8.908  Lidocaine hydrochloride monohydrate, Zingo (TN), Lidocaine (TN)
D08378  3.998(4)  8.431  Pioglitazone (INN), Actos (TN)
D08458  3.998(4)  9.818  Kinidin (TN), Quinidine (BAN)
D08761  3.998(3)  9.818  Tin colloid Tc-99m (TN), Sodium pertechnetate (99mTc) and stannous chloride, anhydrous
D09855  3.998(4)  9.818  Sodium aceneuramate (JAN), Sodium N-acetylneuraminate
D10579  3.998(4) 11.105  Conjugated estrogens and bazedoxifene acetate, Oestrogens conjugated with bazedoxifene acetate, Duavive (TN), Duavee (TN)
D11553  3.998(4)  8.908  Pliaglis (TN), Lidocaine and tetracaine, Synera (TN)
D11645  3.998(4)  7.847  Altafluor benox (TN), Fluorescein sodium and benoxinate hydrochloride
D00182  3.998(4)    -    Norethindrone (USP), Micronor (TN), Camila (TN), Primolut-N (TN), Norethisterone (JP17)
D00185  3.998(4)    -    Estriel (TN), Estriol (JP17/USP)
D01639  3.998(4)    -    Tibolone (JAN/USAN/INN)
D01986  3.998(4)    -    Estriol (TN), Estriol tripropionate (JAN)
D00631  3.998(4)    -    Bepridil hydrochloride (USAN), Bepridil hydrochloride hydrate (JAN), Vascor (TN)
D03991  3.998(4)    -    Encainide hydrochloride (USAN)
D07520  3.998(4)    -    Bepadin (TN), Bepridil (INN)
D07894  3.998(4)    -    Encainide (INN)
D00026  3.998(5) 10.007  Ecolan (TN), L-Cysteine (JP17)
D00073  3.998(5) 11.105  Hydroquinone (USP), Eldoquin (TN)
D00104  3.998(5) 11.105  Amphicol (TN), Econochlor (TN), Chloromycetin (TN), Chloramphenicol (JP17/USP/INN)
D00157  3.998(5)  7.620  Glycyrrhizin (JAN), Glycyrrhizic acid
D00291  3.998(5)  9.818  Desmopressin (INN)
D00472  3.998(5)  7.110  Delta-cortef (TN), Prednisolone (JP17/USP/INN)
D00734  3.998(5)  9.818  Actigall (TN), Urso (TN), Ursodeoxycholic acid (JP17/INN), Ursodiol (USP)
D04106  3.998(5) 11.105  Colimy C (TN), Chloramphenicol and colistin sodium methanesulfonate (JP17)
D04251  3.998(5)  7.110  Cor tyzine (TN), Tetrahydrozoline hydrochloride and prednisolone
D04753  3.998(5)  7.110  Chloramphenicol, fradiomycin sulfate and prednisolone, Chlomy-P (TN)
D04769  3.998(5)  7.110  Aersolin D (TN), Fradiomycin sulfate and prednisolone
D11699  3.998(5)  7.110  Sulfacetamide sodium and prednisolone
D08600  3.998(5)    -    Betimol (TN), Timolol (TN), Timolol (INN)
D10840  3.998(5)    -    Bimatoprost and timolol, Ganfort (TN)
D00378  3.998(5)    -    Betimol (TN), Timolol hemihydrate, Timolol (USAN)
D09007  3.998(5)    -    Tagatose (NF)
D04987  3.998(5)    -    Monoammonium glycyrrhizinate (JAN), Ammonium glycyrrhizate (NF), Glycyron No.1 (TN)
D06917  3.998(5)    -    Inchinkoto
D07688  4.001(6)    -    Zodiac (TN), Chlorpyrifos (BAN)
D01842  4.007(6)    -    Fidarestat (JAN/INN), Aldos (TN), SNK 860
D08066  4.010(4)  8.466  Glamox (TN), Imatinib (INN)
D00341  4.011(5)  9.314  Hydrea (TN), Hydroxyurea (USP), Hydroxycarbamide (JAN/INN), Droxia (TN)
D08470  4.011(5)  9.818  Ajmalicine, Raubasine (DCF), Lamuran (TN)
D04194  4.011(5)    -    Florfenicol (USAN/INN), Nuflor (TN)
D00556  4.011(4)    -    Aminopyrine (JAN), Aminophenazone (INN)
D00066  4.015(5)  8.431  Progesterone (JP17/USP/INN), Crinone (TN), Prometrium (TN)
D08302  4.019(5) 11.105  Ornithine (INN)
D04699  4.019(3)  7.515  Hydrocortisone, fradiomycin sulfate, dibucaine hydrochloride and esculoside, Proctosedyl (TN)
D04776  4.019(3)  7.515  Terra Cortril (TN), Oxytetracycline hydrochloride and hydrocortisone
D04801  4.019(3)  7.515  Eurax H (TN), Hydrocortisone and crotamiton
D08827  4.019(3)  7.515  Posterisan forte (TN), Hydrocortisone and killed escherichia coli suspension
D11855  4.019(3)  7.515  Casporyn hc (TN), Neomycin, polymyxin B sulfate and hydrocortisone, Cortisporin (TN)
D00847  4.026(5)  9.818  Oxycontin (TN), Roxicodone (TN), Oxycodone hydrochloride (USP), Oxecta (TN)
D05312  4.026(5)  9.818  Oxycodone (USAN/INN)
D05462  4.026(5)  9.818  Oxycodone hydrochloride hydrate (JP17)
D11573  4.026(5)  7.214  Oxycodone and ibuprofen
D01625  4.029(5) 11.105  Sodium glucuronate hydrate (JAN), Guronsan (TN), Sodium glucuronate
D00054  4.029(4)  9.496  Inosine (JAN/INN), Inotin (TN)
D04518  4.029(3)  8.803  Inalimarev (CEA, MUC-1, vaccinia virus) (USAN), PANVAC-V, Panvac (TN)
D06067  4.029(3)  9.818  Temodar (TN), Temozolomide (JAN/USAN/INN), Temodal (TN)
D07367  4.032(5) 11.105  Clofenotane (INN), Dichlorojiphinyltrichloroethane, DDT
D00036  4.034(4)  8.908  Niacinamide (USP), Nicotinamide (JP17/INN)
D04252  4.034(4)  8.908  Nicotinamide and papaverine hydrochloride, Stomin A (TN)
D03250  4.036(4)  9.818  Insulin glargine (genetical recombination) (JP17), Insulin glargine (genetical recombination) [Insulin glargin biosimilar 2] (JAN), Insulin glargine (genetical recombination) [Insulin glargin biosimilar 1] (JAN), Insulin glargine (USAN/INN), Lantus (TN)
D11034  4.036(4)  9.818  Insulin glargine and lixisenatide, Insulin glargine (genetical recombination) and lixisenatide, Soliqua (TN)
D03600  4.038(5)  4.942  Creatinine (NF)
D00061  4.043(4) 10.412  Klinit (TN), Xylitol (JP17/NF)
D02072  4.046(5) 11.105  L-Histidine hydrochloride hydrate (JP17), L-Histidine monohydrochloride
D08998  4.046(5)  9.719  Sodium butyrate (USP)
D08999  4.046(5)    -    Sodium caprylate (NF)
D04154  4.050(5)  7.872  Fennel oil (JP17/NF), Fennel oil, bitter
D04388  4.050(5)  7.872  Dried bitter orange peel (TN), Orange peel, bitter, Bitter orange peel (JP17), Powdered bitter orange peel (Non-JPS)
D07974  4.060(4)  9.818  Fluorouracil (TN), Fluorouracil sodium salt
D04550  4.370(3)  4.062  Insulin zinc, prompt (USP), Semilente iletin (TN)
D00346  4.062(4) 10.412  Laniazid (TN), Isoniazid (JP17/USP/INN)
D10210  4.062(4) 10.007  Rifampin, isoniazid and pyrazinamide, Rifater (TN)
D11578  4.062(4) 10.412  Rifampin and isoniazid
D00136  4.068(3)  8.431  Haldol (TN), Haloperidol (JP17/USP/INN)
D00283  4.068(3)  8.061  Clozapine (JAN/USP/INN), Clozaril (TN)
D01340  4.072(5)  7.927  Evzio (TN), Narcan (TN), Naloxone hydrochloride (JP17/USP)
D01723  4.072(5)  9.496  Brocal (TN), Calcium bromide (JAN)
D01731  4.072(5)  9.496  Potassium bromide (JP17/USP), Potassium bromide (TN)
D02055  4.072(5)  9.496  Sodium bromide (JP17), Sodium bromide (TN)
D08249  4.072(5)  7.927  DBL Naloxone (TN), Naloxone (INN)
D08769  4.072(5)  9.125  Rescalmin (TN), Diphenhydramine hydrochloride and calcium bromide
D10250  4.072(5)  7.234  Buprenorphine and naloxone, Bunavail (TN), Suboxone (TN)
D11620  4.072(5)  7.927  Naloxone hydrochloride dihydrate
D05783  4.073(6)    -    Safflower oil (USP), Liposyn (TN)
D00603  4.078(6)    -    Blocadren (TN), Timolol maleate (JP17/USP), Timoptic (TN), Istalol (TN)
D07505  4.078(6)    -    Latanoprost and timolol maleate, Xalacom (TN)
D08493  4.078(6)    -    Cosopt (TN), Dorzolamide hydrochloride and timolol maleate
D09815  4.078(6)    -    Duotrav (TN), Travoprost and timolol maleate
D10298  4.078(6)    -    Brimonidine tartrate and timolol maleate, Aibeta (TN), Combigan (TN)
D10512  4.078(6)    -    Azorga (TN), Brinzolamide and timolol maleate
D10569  4.078(6)    -    Tapcom (TN), Tafluprost and timolol maleate
D03142  4.081(4)  8.333  Blood grouping serums anti-D, anti-C, anti-E, anti-c, anti-e (USP)
D00482  4.088(4)    -    Propoxyphene hydrochloride (USAN), Darvon (TN), Dextropropoxyphene hydrochloride
D04208  4.088(4)    -    Flumetasone (INN), Flumethasone (USAN)
D03165  4.091(3)  9.160  Bromocriptine (USAN/INN)
D00211  4.096(3)  9.719  Rifampin (USP), Rifampicin (JP17/INN), Rifadin (TN), Rimactane (TN)
D00491  4.096(3)  9.125  Abraxane (TN), Taxol (TN), Paclitaxel (JAN/USP/INN)
D02094  4.099(6)  8.719  Thiamine HCL (TN), Thiamine hydrochloride (USP), Thiamine chloride hydrochloride (JP17)
D08580  4.099(6)  8.719  Thiamine (INN), Vitamin B1 (TN)
D00263  4.103(5) 11.105  Keflex (TN), Cefalexin (JP17), Cephalexin
D00906  4.103(5) 11.105  Keftab (TN), Panixine disperdose (TN), Cephalexin (USP), Cefalexin, Cephalexin monohydrate
D01053  4.103(5) 11.105  Benzylpenicillin potassium (JP17), Pfizerpen (TN), Penicillin G potassium (USP)
D02336  4.103(5) 11.105  Benzylpenicillin (INN), Penicillin G
D03438  4.103(5) 11.105  Keftab (TN), Cephalexin hydrochloride (USP)
D05408  4.103(5) 11.105  Benzylpenicillin sodium, Penicillin G sodium (USP), Penicillin G sodium (TN)
D02046  4.103(5)    -    Neocarzinostatin (JAN), Zinostatin (USAN/INN)
D05329  4.103(4)    -    Ringer's injection (USP), Ringer's solution (TN), Ringer's solution (JP17)
D07232  4.106(4)    -    Perchloracap (TN), Potassium perchlorate (USP)
D00750  4.112(6)  9.818  Levamisole hydrochloride (USP), Ergamisol (TN)
D05355  4.112(6)  6.829  Caroid (TN), Papain (USP)
D08114  4.112(6)  9.818  Ketrax (TN), Levamisole (INN)
D04682  4.112(4)    -    Lecithin (NF)
D05625  4.118(5) 10.412  Propane (NF)
D00002  4.119(4)  9.719  Nicotinamide adenine dinucleotide, Nadide (JAN/USAN/INN)
D00616  4.119(4)  9.496  Cardizem (TN), Dilt-CD (TN), Diltiazem hydrochloride (JP17/USP), Dilacor XR (TN), Cartia XT (TN)
D07845  4.119(4)  9.496  Surazem (TN), Diltiazem (INN)
D02329  4.123(5) 11.105  Maltrin (TN), Maltodextrin (NF)
D00307  4.129(4)  7.468  Monodox (TN), Doxycycline (USP), Doxycycline hydrate, Oracea (TN), Vibramycin (TN)
D01738  4.129(4)  5.749  Definity (TN), Perflutren (JAN/USAN/INN)
D02129  4.129(4)  7.468  Periostat (TN), Lymepak (TN), Doxycycline hydrochloride hydrate (JP17), Doxycycline hyclate (USP), Vibra-tabs (TN), Doryx (TN)
D03903  4.129(4)  7.468  Doxycycline calcium (USP)
D04285  4.129(5)  9.125  Glyceol (TN), Concentrated glycerin and fructose
D07776  4.129(4)  5.926  Daunorubicin (INN), DaunoXome (TN), DM
D07876  4.129(4)  7.468  Doxycycline (TN), Doxychel (TN), Doxycycline (INN)
D07877  4.129(4)  7.468  Doxycycline hydrochloride, Vibramycin (TN)
D08918  4.129(4)  9.125  CEA, MUC-1, fowlpox virus, Falimarev (USAN)
D09738  4.129(4)  9.719  Asparagine (NF), L-Asparagine monohydrate
D01642  4.129(4)    -    Bismuth subnitrate (JP17/USP), Mammol (TN)
D00008  4.131(5)  6.931  Oxyfull (TN), Hydrogen peroxide (USP), Oxydol (JP17)
D00768  4.131(4) 10.007  Carisoprodol (JAN/USP/INN), Soma (TN)
D00190  4.131(5)  7.420  Rutin hydrate (JAN), Rutin trihydrate
D03309  4.131(5) 11.105  Calcium silicate (NF)
D03846  4.131(5)  8.803  Dimethyl fumarate (JAN/USAN), Tecfidera (TN)
D04379  4.131(5) 11.105  Methaphyllin (TN), Propantheline bromide, sodium copper chlorophyllin and magnesium silicate
D04675  4.131(5)  7.420  Melilot extract and rutin hydrate, Esberiven (TN)
D04839  4.131(5) 11.105  Magnesium silicate (JP17/NF), Florisil
D05341  4.131(5) 11.105  Palmitic acid (NF)
D05848  4.131(5)  9.818  beta-Sitosterol glucoside, Sitogluside (USAN/INN), Eleutheroside A, Daucosterol
D07751  4.131(5)  9.496  Venalot mono (TN), Coumarin (DCF)
D08499  4.131(5)  7.420  Rutoside (INN), Rutin, Venoruton (TN)
D10238  4.131(5)    -    Coicis fructus cum involucris, Coix fruit with involucre (Non-JPS), Adlay
D00152  4.131(5)    -    Phloroglucinol (JAN), Dilospan S (TN)
D00549  4.138(3)  8.026  Diprivan (TN), Propofol (JAN/USP/INN)
D02316  4.144(6) 10.412  Pectin (USP)
D11178  4.148(6)  5.154  Tobramycin and dexamethasone, Tobradex (TN)
D02971  4.151(3)  9.719  Aprotinin (USP/INN), Trasylol (TN)
D00287  4.152(3)  8.161  Cyclophosphamide hydrate (JP17), Cyclophosphamide (USP), Neosar (TN), Cytoxan (TN)
D00473  4.152(3)  6.927  Prednisone monohydrate, Meticorten (TN), Prednisone (USP), Rayos (TN), Deltasone (TN)
D07760  4.152(3)  8.161  Cytoxan (TN), Cyclophosphamide (TN), Cyclophosphamide (INN)
D01953  4.152(5) 10.412  Ovahormon (TN), Estradiol benzoate (JP17/USP)
D04465  4.152(5) 10.412  Hydroxyprogesterone caproate and estradiol benzoate, Lutes (TN)
D00106  4.155(3)  8.719  Epoprostenol (TN), Prostacyclin, Prostaglandin I2, Epoprostenol (USAN/INN)
D03634  4.155(4)  9.818  Mercaptamine (INN), Cysteamine (USAN)
D03635  4.155(4)  9.818  Cystaran (TN), Cysteamine hydrochloride (USAN), Systadrops (TN)
D01989  4.156(4)    -    Estriol benzoate diacetate, Estriol diacetate benzoate (JAN), Holin (TN)
D00145  4.160(3)  8.719  Trimpex (TN), Proloprim (TN), Trimethoprim (JAN/USP/INN)
D00285  4.160(3)  8.719  Bactrim (TN), Sulfamethoxazole and trimethoprim, Septra (TN), Co-Trimoxazole (BAN)
D11085  4.160(3)  8.719  Trimethoprim hydrochloride, Trimpex (TN), Primsol (TN)
D11086  4.160(3)  8.208  Polymyxin B and trimethoprim, Polytrim (TN)
D00007  4.164(4) 11.105  L-Glutamic acid (JP17), Glutamic acid (USP)
D00058  4.164(4) 11.105  Gammalon (TN), gamma-Aminobutyric acid (JAN)
D04341  4.164(4) 11.105  DL-Glutamic acid, Glutamic acid (USAN)
D07539  4.164(4) 11.105  Glutamic acid hydrochloride, Hypochylin (TN)
D04365  4.165(6)  7.872  Glycyrrhiza (JP17), Glycyrrhizae radix praeparata, Glycyrrhizae radix, Powdered glycyrrhiza (JP17), Prepared glycyrrhiza (JP17), Liquorice, Licorice (NF), Glycyrrhiza (TN)
D00487  4.167(3)  9.818  Pyridostigmine bromide (JP17/USP/INN), Mestinon (TN)
D00558  4.167(3) 10.007  Carbidopa (USP), Lodosyn (TN), Carbidopa hydrate (JP17)
D01211  4.167(4) 11.105  Wellcovorin (TN), Uzel (TN), Leucovorin calcium (USP), Calcium folinate (JP17)
D01653  4.167(3) 11.105  Benserazide hydrochloride (JP17)
D02280  4.167(4) 11.105  Monosodium glutamate (NF), Sodium L-glutamate hydrate (JAN), Ancoma (TN), Monosodium L-glutamate monohydrate
D02365  4.167(4) 11.105  Oxipurinol (INN), Oxypurinol (USAN)
D03082  4.167(3) 11.105  Benserazide (USAN/INN)
D04842  4.167(4)  7.872  Formalin and cresol, Formalin cresol (TN)
D04861  4.167(4)  7.872  Palpack V (TN), Cresol, formalin, clove oil and zinc oxide
D05200  4.167(3)  6.056  Merital (TN), Nomifensine maleate (USAN)
D08703  4.167(4) 11.105  Compound epithanate G (TN), L-Glutamic acid, pipethanate hydrochloride and aluminum hydroxide-sodium bicarbonate co-precipitate
D01960  4.167(4)    -    Cresol (JP17/NF)
D00780  4.167(3)    -    Bromocriptine mesylate (USP), Bromocriptine mesilate (JP17), Parlodel (TN)
D01043  4.167(3)    -    Dimethyl sulfoxide (JAN/USP/INN), Rimso-50 (TN), Zymso (TN)
D08261  4.167(3)    -    Prostigmin (TN), Neostigmine (BAN)
D00017  4.180(6)  7.872  Formaldehyde (USP), Formalin (JP17)
D03313  4.180(6)  7.738  Paraffin (TN), Paraffin (JP17/NF)
D04846  4.180(6)  7.872  Formalin and Guaiacol, Formalin-guaiacol FG (TN)
D06772  4.180(6)  9.314  Ginseng (TN), Powdered ginseng (JP17), Ginseng (JP17)
D04306  4.192(3)  9.719  Gauze, petrolatum
D04934  4.192(4)  8.719  Mercuric amidochloride, Mercury amide chloride, Mercury, ammoniated (USP)
D05331  4.192(3) 11.105  Petrolatum (USP), Yellow petrolatum (JP17)
D06323  4.192(3)  8.621  Wax, emulsifying (NF)
D05287  4.192(3)    -    Simple ointment (JP17), Simple ointment (TN)
D04474  4.192(3)    -    Hydroxyethyl cellulose (JP17/NF), Cellosize (TN)
D00748  4.193(6)  7.620  Aldesleukin (USAN/INN), Proleukin (TN), Interleukin-2
D00052  4.194(6)  8.466  Versene acid (TN), EDTA, Edetic acid (NF/INN)
D08514  4.196(4)  9.160  Sildenafil (INN), Aphrodil (TN)
D00528  4.200(4)  9.314  Caffeine (USP), Respia (TN), Anhydrous caffeine (TN), Anhydrous caffeine (JP17)
D01453  4.200(4)  9.314  Caffeine monohydrate, Caffeine (TN), Caffeine hydrate (JP17)
D02409  4.200(4)  9.314  Sodium caffeine benzoate, Annaca (TN), Caffeine and sodium benzoate (JP17)
D03844  4.200(4)  9.314  Hydantol F (TN), Phenytoin, phenobarbital and caffeine and sodium benzoate
D03980  4.200(4)  9.314  Isopropylantipyrine, allyl isopropyl acetyl urea, phenacetin and caffeine, Saridon (TN)
D04019  4.200(4)  9.314  Isopropylantipyrine, allyl isopropyl acetyl urea, phenacetin and anhydrous caffeine, Sedes G (TN)
D07585  4.200(4)  9.314  Cleamine A (TN), Isopropylantipyrine, ergotamine tartrate and anhydrous caffeine
D07600  4.200(4)  9.314  Cafergot (TN), Migergot (TN), Ergotamine tartrate and anhydrous caffeine
D07611  4.200(4)  9.314  Simetride and anhydrous caffeine, Kyorin AP2 (TN)
D08454  4.200(4) 10.412  Pyridoxine (INN), Vitamin B6 (TN)
D01046  4.201(7) 10.412  Genapax (TN), Methylrosanilinium chloride (JP17/INN), Methylrosaniline chloride, Crystal violet, Gentian violet (USP)
D00070  4.204(5)  8.466  Folicet (TN), Folic acid (JP17/USP/INN)
D03332  4.208(5)  9.125  Lysozyme hydrochloride (JP17), Mucozome (TN)
D08152  4.208(5)  9.125  Muramidase, Lysozyme (DCF)
D08800  4.212(3)  8.719  Human haptoglobin, Haptoglobin (TN)
D06687  4.212(4)  9.125  Astragalus root (JP17), Astragali radix (TN)
D09526  4.212(3)  8.208  Margarita, Pearl
D06701  4.212(4)    -    Trichosanthes root (JP17), Trichosanthes root (TN)
D06728  4.212(4)    -    Asiasarum root (TN), Asiasarum root (JP17)
D06731  4.212(4)    -    Powdered gardenia fruit (JP17), Gardenia fruit (JP17), Gerenia fruit (TN)
D06737  4.212(4)    -    Lycium bark (JP17)
D11546  4.212(4)    -    Amylopectin (USAN)
D05276  4.213(5)    -    Gastrozepin (TN), Pirenzepine hydrochloride hydrate (JP17)
D08389  4.213(5)    -    Pirenzepine (INN)
D01297  4.213(5)    -    Pirenzepine hydrochloride (USAN)
D00010  4.213(4)  9.719  Acetic acid (JP17/NF), Acetic acid, glacial (USP), Glacial acetic acid (JP17), Acetasol (TN)
D01083  4.213(4)  8.466  Calcium hydroxide (JP17/USP), Calkyl (TN)
D01168  4.213(4)  8.466  Potassium hydroxide (TN), Potassium hydroxide (JP17/NF)
D01169  4.213(4)  8.466  Sodium hydroxide (JP17/NF), Sodium hydroxide (TN)
D04393  4.213(4)  8.466  Maalox (TN), Sakloft (TN), Aluminum hydroxide and magnesium hydroxide
D04856  4.213(4)  8.466  Sulfathiazole, diethylaminoethyl p-butylaminobenzoate hydrochloride, iodoform and calcium hydroxide, Calvital (TN)
D08647  4.218(5)  8.026  Atenen (TN), Triphosadenine disodium salt, Adenosine triphosphate disodium
D02261  4.221(5)  8.708  Quinine hydrochloride dihydrate, Quinine hydrochloride hydrate (JP17), Quinine hydrochloride (TN)
D02310  4.221(3)  9.818  Propionic acid (NF)
D06346  4.221(5) 11.105  Xylose (USP), Xylo-Pfan (TN), D-Xylose
D07507  4.221(3)  9.026  Combivir (TN), Lamivudine zidovudine (TN), Zidovudine and lamivudine
D08460  4.221(5)  8.708  Quinine (BAN), Kinder Quinina (TN)
D08461  4.221(5)  8.708  Quinine dihydrochloride, Quinine (TN)
D10534  4.221(5)  9.818  Enstilar (TN), Taclonex (TN), Calcipotriene and betamethasone dipropionate, Calcipotriol and betamethasone dipropionate, Dovobet (TN)
D10838  4.221(3)  9.026  Trizivir (TN), Abacavir, lamivudine and zidovudine
D11521  4.221(3)  9.026  Lamivudine, nevirapine and zidovudine
D01637  4.221(5)    -    Diprolene (TN), Betamethasone dipropionate (JP17/USP), Sernivo (TN), Rinderon-DP (TN)
D06887  4.224(7) 11.105  Lotusate (TN), Talbutal (INN)
D00559  4.225(3) 11.105  Pramipexole dihydrochloride monohydrate, Pramipexole dihydrochloride (USP), Mirapex (TN), Pramipexole hydrochloride hydrate (JAN)
D05575  4.225(3) 11.105  Pramipexole (USAN/INN)
D02058  4.229(5)    -    Zinc chloride (TN), Zinc chloride (JP17/USP)
D00205  4.230(5) 11.105  Terramycin (TN), Oxytetracycline dihydrate, Oxytetracycline (JAN/USP/INN)
D01264  4.230(5)  9.818  Daunorubicin hydrochloride (JP17/USP), Cerubidine (TN)
D01596  4.230(5) 11.105  Terramycin (TN), Oxytetracycline hydrochloride (JP17/USP)
D02144  4.230(5) 11.105  Oxytetracycline hydrochloride and polymixin B sulfate, Terramycin ophthalmic (TN)
D04249  4.230(5) 11.105  Oxytetracycline hydrochloride and hydrocortisone acetate, Terra-cortril (TN), Tetrazol (TN)
D05322  4.230(5) 11.105  Terramycin (TN), Oxytetracycline calcium (USP)
D11390  4.230(5)  9.818  Daunorubicin and cytarabine, Vyxeos (TN)
D05373  4.231(4)    -    Maltose hydrate (JP17), Maltose 10 (TN)
D00044  4.231(4)    -    Madoros (TN), Maltose (NF)
D01082  4.234(5)    -    Calpan (TN), Calcium pantothenate (JP17/USP/INN)
D11519  4.234(5)    -    Sodium pantothenate
D00293  4.239(5)  8.908  Valium (TN), Diazepam (JP17/USP/INN), Diastat (TN)
D00550  4.239(5)  8.431  Midazolam (JAN/USP/INN), Dormicum (TN), Nayzilam (TN), Buccolam (TN)
D00696  4.239(5)  8.026  Versed (TN), Midazolam hydrochloride (USAN)
D00710  4.239(5) 11.105  Depakene (TN), Selenica (TN), Valproate sodium (USAN), Sodium valproate (JP17)
D08351  4.239(5)  9.818  Phenformin (BAN)
D08352  4.239(5)  9.818  Phenformin hydrochloride, Debei (TN)
D08667  4.239(5) 11.105  Calcium valproate, Valproic acid calcium salt, Convulsofin (TN)
D08074  4.239(5)    -    Penbutolol (INN)
D08363  4.239(5)    -    Phenylbutazone calcium, Peralgin (TN)
D08364  4.239(5)    -    Ambene (TN), Phenylbutazone sodium
D00450  4.239(5)    -    Sulfisoxazole (JP17/USP), Sulfafurazole (INN), Gantrisin (TN)
D00510  4.239(5)    -    Phenylbutazone (JP17/USP/INN), Azolid (TN)
